# BODIPY as electron withdrawing group for the activation of double bonds in asymmetric cycloaddition reactions†
†Electronic supplementary information (ESI) available. CCDC 1880124. For ESI and crystallographic data in CIF or other electronic format see DOI: 10.1039/c9sc00959k


**DOI:** 10.1039/c9sc00959k

**Published:** 2019-03-20

**Authors:** Andrea Guerrero-Corella, Juan Asenjo-Pascual, Tushar Janardan Pawar, Sergio Díaz-Tendero, Ana Martín-Sómer, Clarisa Villegas Gómez, José L. Belmonte-Vázquez, Diana E. Ramírez-Ornelas, Eduardo Peña-Cabrera, Alberto Fraile, David Cruz Cruz, José Alemán

**Affiliations:** a Organic Chemistry Department, Módulo 1 , Universidad Autónoma de Madrid , Madrid-28049 , Spain . Email: jose.aleman@uam.es ; http://www.uam.es/jose.aleman; b Chemistry Department , Universidad Autónoma de Madrid , Madrid-28049 , Spain; c Institute for Advanced Research in Chemical Sciences (IAdChem) , Universidad Autónoma de Madrid , Madrid-28049 , Spain; d Condensed Matter Physics Center , IFIMAC , Universidad Autónoma de Madrid , 28049 Madrid , Spain; e Chemistry Department , División de Ciencias Naturales y Exactas , Universidad de Guanajuato , Noria Alta S/N , 36050 Guanajuato , Gto , Mexico

## Abstract

In this work we have found that a BODIPY can be used as an electron withdrawing group for the activation of double bonds in asymmetric catalysis. The synthesis of cyclohexyl derivatives containing a BODIPY unit can easily be achieved *via* trienamine catalysis. This allows a new different asymmetric synthesis of BODIPY derivatives and opens the door to future transformation of this useful fluorophore. In addition, the Quantum Chemistry calculations and mechanistic studies provide insights into the role of BODIPY as an EWG.

## Abstract 2

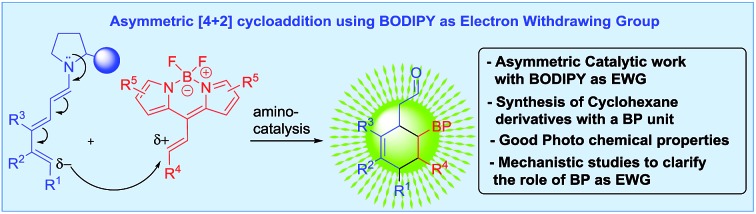
BODIPY as an EWG in asymmetric catalysis is presented.

## Introduction

**Scheme 1:**
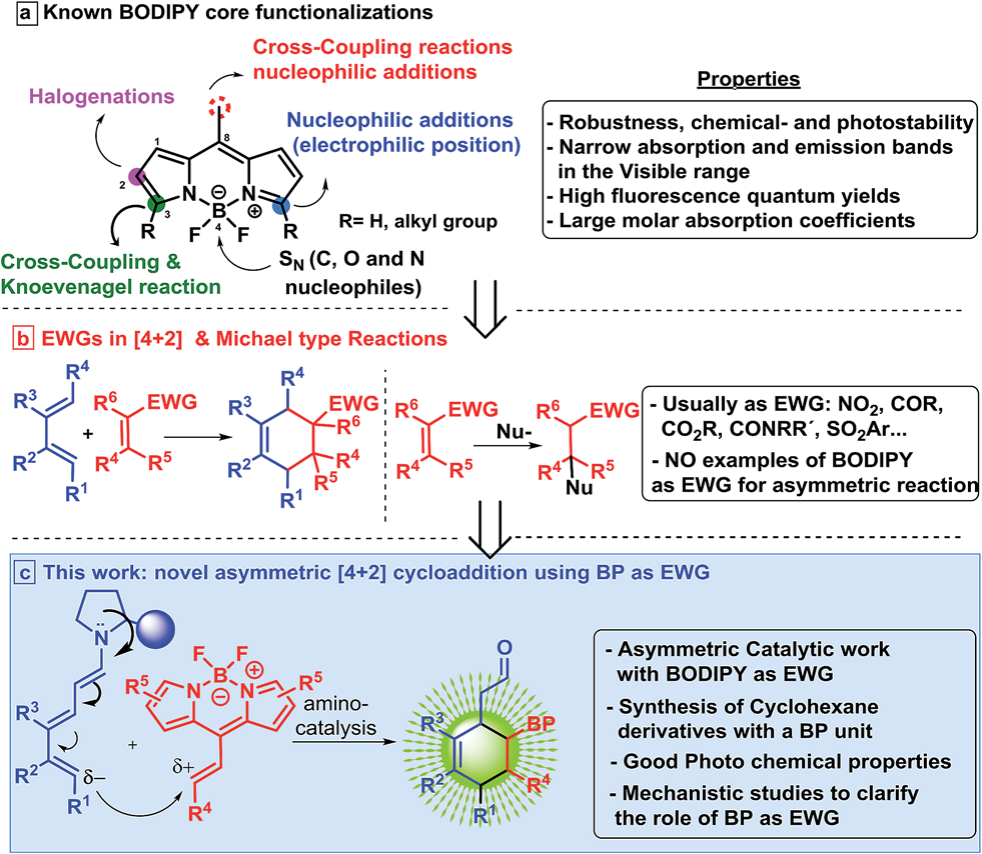
Background and present work in the [4 + 2] cycloaddition reaction *via* trienamine with alkenyl BODIPY derivatives (BP = BODIPY).

BODIPY dyes (BOron DIPYrromethene) are a remarkable family of fluorophores that have been studied in recent years due to their excellent robustness, and chemical- and photo-stability.^1^ The structure of the BODIPY derivatives is formed by two pyrrole units linked by a carbon and complexed with a di-substituted boron atom, mainly a BF_2_ motif, which forms the core scaffold (see top, Scheme 1). They show impressive spectroscopic properties such as narrow absorption and emission bands in the visible wavelength range, high fluorescence quantum yields and large molar absorption coefficients among others.1b,^2^ As a result of these interesting characteristics, this class of fluorophores has attracted a lot of attention due to their numerous applications, for instance, as labelling reagents, in the bioimaging of living cells,^3^ as radiotracers for positron emission tomography,^4^ photocatalysts^5^ or photodynamic therapy (PDT).^6^ In addition, the introduction of stereogenic centres in these type of structures is of great importance as it is possible to modulate the BODIPY photophysics. Therefore, chiroptical applications based on circular dichroism (CD) and circularly polarized luminescence (CPL) can be used in devices for optical storage and enantioselective CPL sensors, among others.7a,b

Different modes of functionalization of BODIPY dyes have been described in the literature. They present eight different positions that can be modulated, causing changes and modifications of the spectral and photochemical properties.^1^,7c Initial studies into the reactivity and derivatization of these important building blocks have been carried out by Werz,8a,b Ziessel,8*c* Shinokubo,8*d* Burgess,8*e* Liras,8*f* Bröring,8*g* de la Moya,8h,i and us.8*j*

However, in spite of these efforts, very little is known about the catalytic asymmetric synthesis of BODIPY derivatives. Two main reactivities can be found: aromatic type reactivities (see top Scheme 1 coloured green, pink and blue), which are related to the direct regioselective halogenations that can be performed at different positions,^9^ aromatic substitutions,^10^ as well as cross-coupling reactions;^11^ and reactivity at the methyl of the methylene bridge, the most acidic position (see top Scheme 1 coloured red), although the number of these examples is scarce.1a,^11^ This latter position can be deprotonated and can react with diethyl ketomalonate,8*a* or aldehydes.^12^ Moreover, de la Moya group have shown that boron functionalization can be easily achieved as well, introducing different alcohol or amine derivatives.8h,i

One of the most used strategies to polarize double bonds in asymmetric catalysis is the employment of Electron Withdrawing Groups (EWGs, middle Scheme 1), which decrease the energy of the LUMO, thus favouring the interaction with the HOMO of the nucleophile. This strategy has been widely used for Michael-type nucleophilic additions or stepwise [4 + 2] cycloadditions. For this latter reaction, trienamine catalysis^13^ has shown to be one of the most prominent strategies,^14^ using double bonds activated with nitro,14c,d azlactones14*a* or cyanoacetate groups14*b* as dienophiles (middle Scheme 1). These authors have described this [4 + 2] reaction as an asynchronous cycloaddition,^15^*via* a Michael addition followed by an intramolecular iminium ion reaction. In all these examples, very strong EWGs, *e.g.* nitro group, or two nitriles, at the double bond were used in order to achieve the desired reactivity. Therefore, based on electron-withdrawing character of the BODIPY core,^16^ we wondered if it would be possible to use this interesting fluorescent moiety as an EWG of a double bond located at the 8-position to perform an asymmetric [4 + 2] cycloaddition (bottom Scheme 1). In this work, we describe the catalytic asymmetric synthesis of chiral BODIPY cyclohexane derivatives, using trienamine aminocatalysis *via* a Diels–Alder reaction (Scheme 1c). In addition, the optical properties of the adducts and DFT calculations, which explain the mechanism and the role of the BODIPY as an EWG have been performed.

## Results and discussion

We started the present study with the reaction between the dienal **1a** and the BODIPY **2a** in the presence of the Jørgensen–Hayashi catalyst **3a** in chloroform at room temperature. We found that the reaction gave the desired product **5a** with a very low conversion (entry 1, Table 1). In order to improve this preliminary result, we tested the addition of benzoic acid as an additive (entry 2), increasing the conversion to 32%. Latterly, when the temperature was increased to 45 °C, full conversion was achieved (entry 3). Following this, different aminocatalysts **3b–d** were tried (entries 4–6). Interestingly, the bulkiest catalyst **3b** or the hydrogen bond type catalysts **3c** and **3d** did not provide any conversion to the product **5a**. Different solvents under **3a** catalysis were then examined (entries 7–10). Chlorinated solvents and THF gave only modest results, but very apolar solvents such as toluene and *p*-xylene provided full conversions and very high enantioselectivities (94 and 96% ee). The catalytic loading was decreased to 10 mol%, achieving the product **5a** with 96% ee, full conversion, and 82% isolated yield (entry 11). However, when the catalyst loading was 5 mol%, only 10% conversion was found (entry 12). Once the best conditions had been determined, we carried out the scope of the reaction using different aldehydes **1** and BODIPYs **2** (Table 2).

**Table 1:** Screening of reaction conditions for the synthesis of **5a**^*a*^

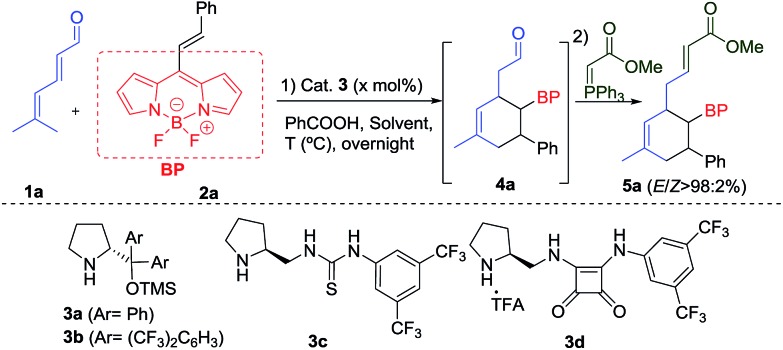					
Entry	Cat. [mol%]	Solvent	Temp (°C)	Conv^*b*^ (%)	ee^*c*^ (%)
1^*d*^	**3a** (20)	CHCl_3_	rt	9	—
2	**3a** (20)	CHCl_3_	rt	32	92
3	**3a** (20)	CHCl_3_	45	100	84
4	**3b** (20)	CHCl_3_	45	n.r.	—
5	**3c** (20)	CHCl_3_	45	n.r.	—
6	**3d** (20)	CHCl_3_	45	n.r.	—
7	**3a** (20)	CH_2_Cl_2_	45	15	n.d.^*f*^
8	**3a** (20)	THF	45	c.m.^*e*^	—
9	**3a** (20)	Toluene	45	100	94
10	**3a** (20)	*p*-Xylene	45	100	96
**11**	**3a** (10)	*p*-Xylene	**45**	**100 (82)** ^*g*^	**96**
12	**3a** (5)	*p*-Xylene	45	10	n.d.^*f*^

^*a*^0.05 mmol of **2a**, 0.13 mmol of **1a** in 0.5 mL of the indicated solvent and the same amount of PhCO_2_H as catalyst loading.

^*b*^Conversion and *Z*/*E* ratio determined by ^1^H NMR analysis of the crude mixture.

^*c*^Determined by SFC.

^*d*^Without PhCO_2_H.

^*e*^Complex mixture.

^*f*^Not determined.

^*g*^Isolated yield after FC in brackets.

**Table 2:** Scope of reaction with different aldehydes **1** and BODIPYs **2**^*a*^

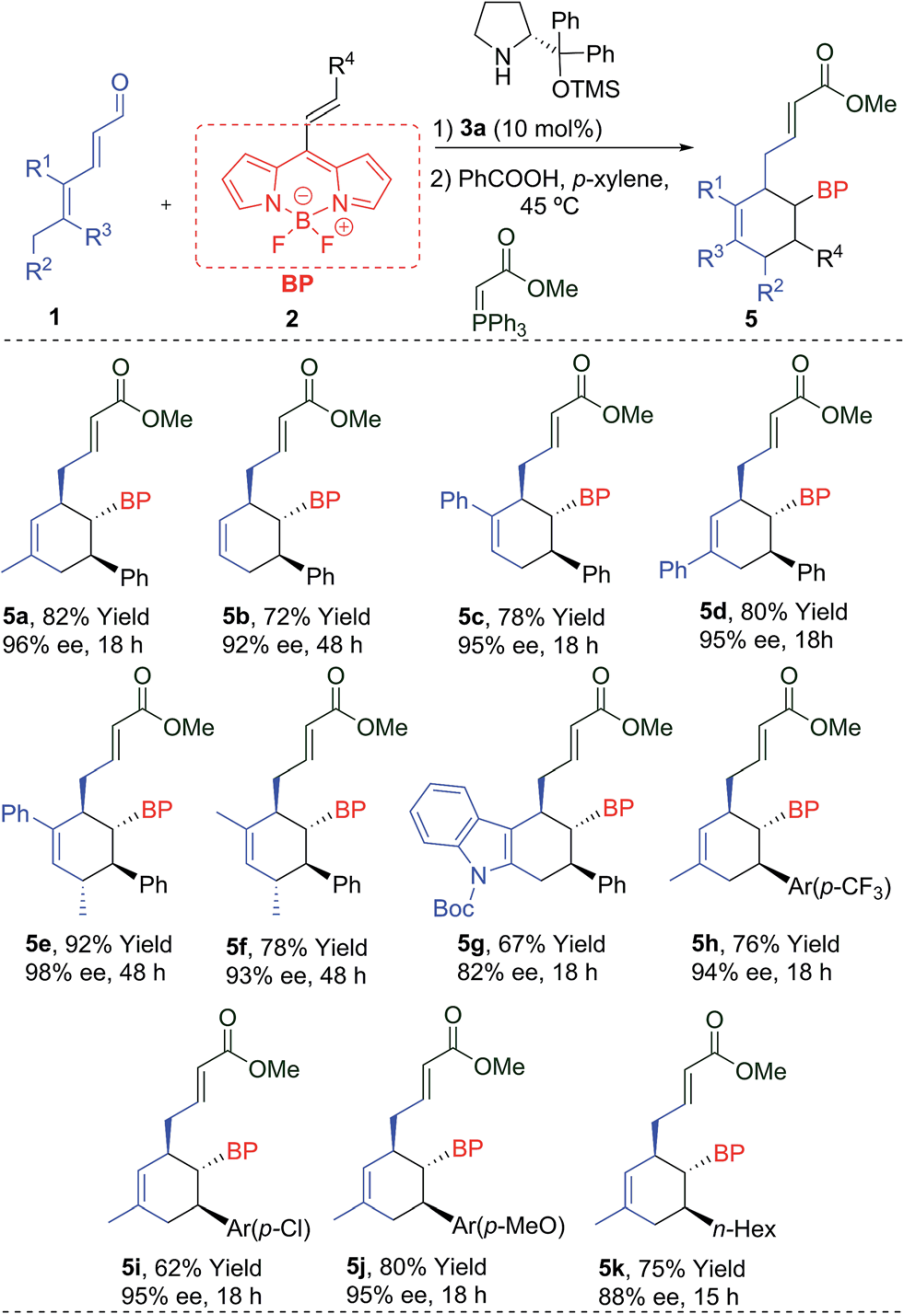

^*a*^Conditions: 0.1 mmol of **2**, 0.25 mmol of **1**, 10 mol% of **3a** and 10 mol% of PhCO_2_H in 1.0 mL of *p*-xylene. Enantiomeric excess determined by SFC.

**Fig. 1:**
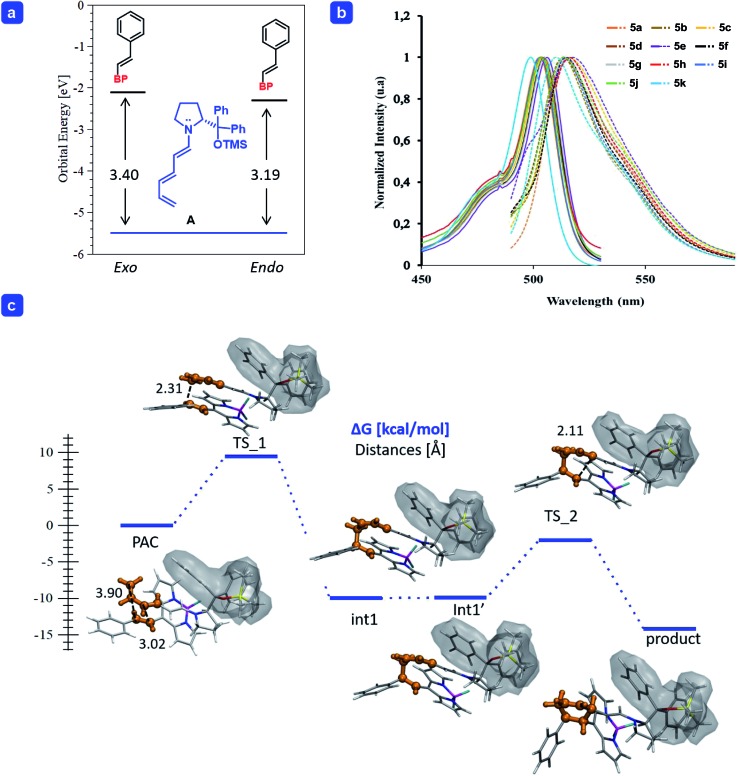
(a) Energy (in eV) of the frontier molecular orbitals calculated for trienamine **A**, and the BODIPY **2a** for the *endo* and *exo* approaches. (b) Absorption and emission (dash-line) spectra of BODIPYs **5a–k** (see ESI for details†). (c) Gibbs free energy profile of the *endo*-[4 + 2] cycloaddition of the trienamine formed from **1b** and catalyst **3a** to the double bond **2a**. The reactive part is highlighted in orange and the shadow wraps the catalyst. Energies in kcal mol^–1^. Geometry optimization was carried out at the M06-2X/6-31G(d,p) level of theory and single point energies including solvent at the SMD_(*p*-xylene)_/M06-2X/6-31+G(d,p) level of theory.

The reaction worked even when all substituents were hydrogen (R^1^ = R^2^ = R^3^ = H), giving **5b** with a 92% ee and a 72% yield. The cyclohexanes **5c** and **5d**, with different substitutions at R^1^ or R^3^ (Ph), were also obtained with excellent yields and enantioselectivities after 18 h. An additional stereogenic centre can be also obtained using **1e** (R^1^ = Ph, R^2^ = R^3^ = Me) and **1f** (R^1^ = R^2^ = R^3^ = Me), that allows access to the products **5e** and **5f** with complete stereocontrol at the four stereogenic centres. An interesting indole derivative **5g** was obtained with a very good ee and a good yield. The use of different EDGs (*p*-MeO) and EWGs (*p*-CF_3_ or *p*-Cl) at the aromatic ring of the double bond, gave the final products **5h–j** with good results. Aliphatic derivatives (**5k**) were also tolerated. We also measured the absorption and emission spectra of these new BODIPYs (**5a–k**), which are comparable with other previously related derivatives,1b,^7^ described in the literature (top-right, Fig. 1b).

**Scheme 2:**
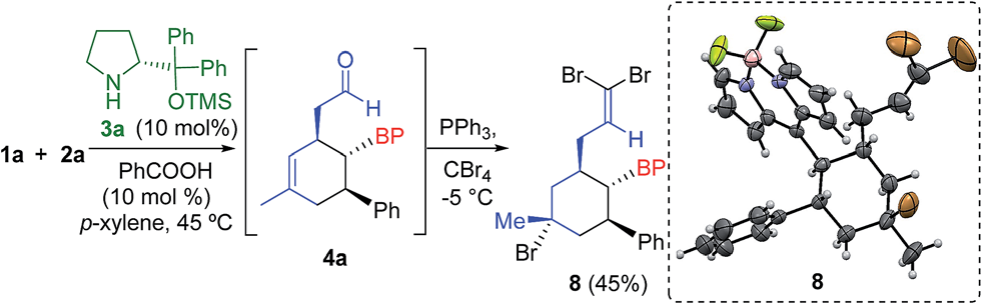
Derivatization of compound **4a** and X-ray analysis of compound **8**.

The absolute configuration was determined by derivatization of the intermediate **4a**, yielding the olefin **8** with concomitant bromination of the cyclohexene double bond (Scheme 2). Therefore, we assigned the configuration of compounds **5** as 1′*S*, 2′*S*, 3′*R* using X-ray analysis.^17^

In order to shed light onto the reactivity, we performed a Frontier Molecular Orbital (FMO) analysis, using the density functional theory (DFT),^18^ frequently employed to explain the reactivity in pericyclic reactions^19^ as in the one presented here (for more details see ESI†). The orbitals of the reagents in the ground state are used to predict the way the reaction proceeds (both orientation and reaction rate). The addition of trienamine **A** (**1b**) to the double bond of BODIPY **2a** is governed mainly by the overlap between the Highest Occupied Molecular Orbital of the nucleophile (HOMO_trienamine_) and the Lowest Unoccupied Molecular Orbital of the electrophile (LUMO_dienophile_), leading to the correct orientation. In addition, the HOMO–LUMO energy gap is related to the reaction rate (*k*), which is enhanced when the gap decreases.^20^ Therefore, if we compare the reaction of two different electrophiles with the same nucleophile, the lowest HOMO–LUMO gap will explain the highest catalytic efficiency. The energy difference between the frontier orbitals in the reaction of **A** with **2a**, the energy gap (Δ*E* = LUMO–HOMO) was higher, and too large, for the *exo*- than for the *endo*-approach (3.40 and 3.19 eV respectively, top-left, Fig. 1a).^21^ Therefore, we only considered the reaction energy profile for the *endo*-approach (bottom, Fig. 1c).

We found that the reaction takes place in a stepwise fashion (bottom, Fig. 1c) as reported in previous examples in the literature.^15^ Once the pre-association complex (PAC) is formed,^22^ the first C–C bond between the terminal carbon of the trienamine **A** and the β-carbon of **2a** is formed with a barrier of 9.5 kcal mol^–1^ (**TS_1**), which is the stereoselective limiting step barrier. Then, after a series of rotations with negligible energy cost (from the intermediate **Int-1** to **Int-1′**) it forms the second C–C bond with a barrier of 7.9 kcal mol^–1^ (**TS_2**) to yield the final adduct which is easily cleaved, *via* hydrolysis, releasing the catalyst **3a** and the desired product **4a**.

**Scheme 3:**
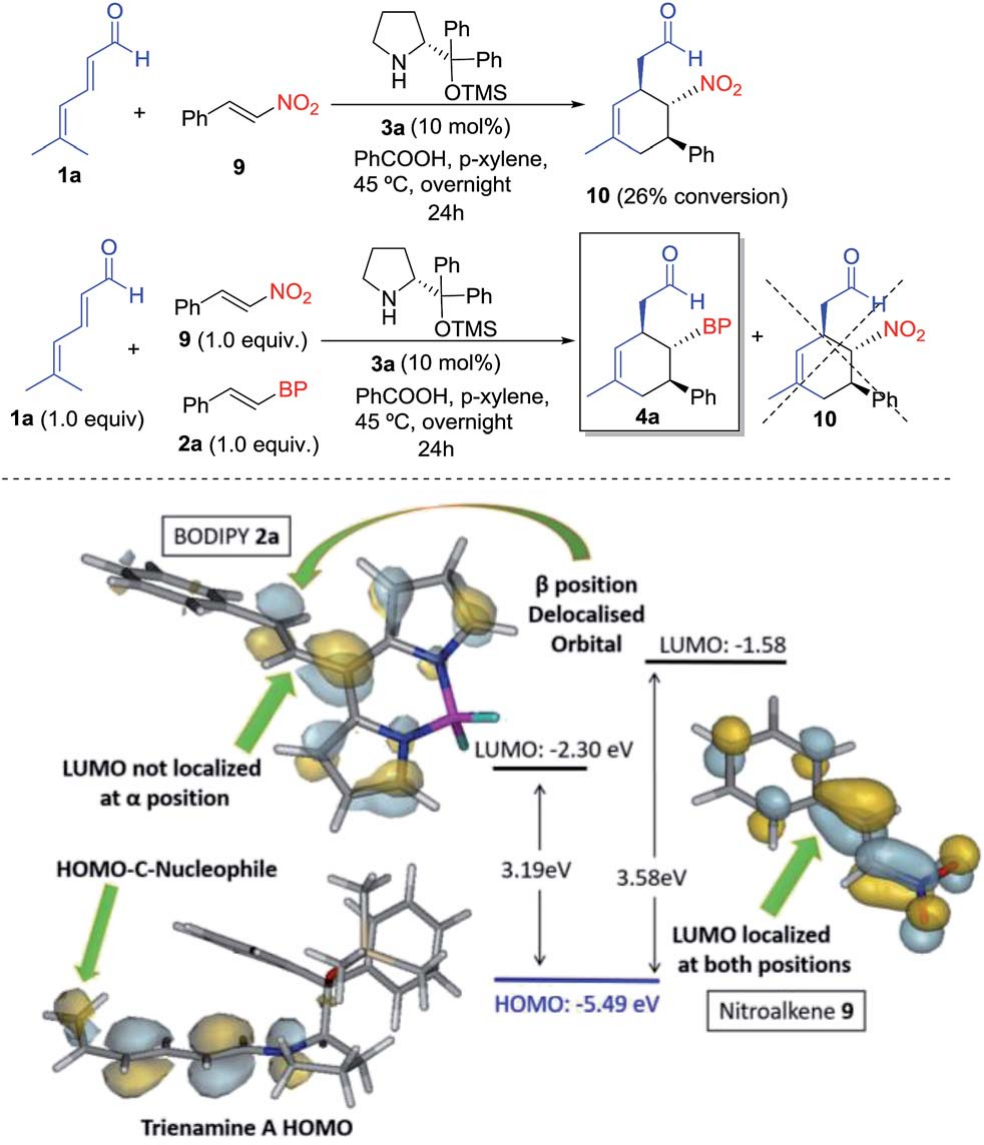
Top: reaction and reactivity comparison of BODIPY **2a** with nitroalkene **9**. Bottom: orbital analysis of **2a**, **9** and trienamine **A**.

Finally, to study the relative reactivity of BODIPY **2a**, we compared its reaction with other known dienophiles in trienamine chemistry such as the nitrostyrene.14*d* The reaction of dienal **1a** with nitroalkene **9** yielded **10** with a 26% conversion after 24 h under the same reaction conditions (top, Scheme 3). We then carried out a competitive reaction between **2a** and **9**, and found that only the BODIPY derivative reacted, without any traces of product **10**, thus highlighting the higher reactivity of **2a**. The origin of this notable difference in the reactivity was analyzed with the frontier orbitals of trienamine **A**, and dienophiles **2a** and **9** (bottom, Scheme 3). The HOMO orbital of trienamine **A** is delocalized over the two central double bonds, between the nitrogen and the terminal nucleophilic carbon atom that will attack the β position of the BODIPY double bond. The LUMO orbital of **2a** is delocalized over the BODIPY with an important contribution at the β position of the double bond and without any contribution at the α position. This explains the regioselectivity, as the β carbon of **2a** is the first to react. However, in the case of nitroalkene **9**, the LUMO orbital is fully delocalized through the molecule with contributions from α and β carbon atoms. In addition, the HOMO–LUMO gap is much lower for **2a** (3.19 eV) than for **9** (3.58 eV). This means that the BODIPY is a better EWG than the NO_2_ for this reaction and explains the higher reactivity of the BODIPY derivatives **2** when compared with nitrostyrene **9**.

## Conclusions

In conclusion, we have shown that the BODIPY can be used as an electron withdrawing group for the activation of double bonds in asymmetric catalysis. Indeed, the BODIPY acts as a stronger EWG than the nitro group. In this work, we have applied this characteristic for the synthesis of asymmetric cyclohexyl derivatives *via* trienamine catalysis, that contain a BODIPY unit in their structure, allowing a new functionalization of these fluorophores. In addition, we have been able to explain the observed reactivity with Quantum Chemistry calculations, confirming the role of the BODIPY as an EWG in the double bond. The new reactivity here presented can be used in the future for further asymmetric transformations.

## Conflicts of interest

There are no conflicts to declare.

## Supplementary Material

Supplementary information

Crystal structure data
