# Supplementary material for: BODIPY as electron withdrawing group for the activation of double bonds in asymmetric cycloaddition reactions
Source: Chem Sci. 2019 Mar 20;10(15):4346–51. doi: 10.1039/c9sc00959k (PMC6472058; doi:10.1039/c9sc00959k)
Supplement: Supplementary file 1 [file SC-010-C9SC00959K-s001.pdf]

Electronic Supplementary Material (ESI) for Chemical Science.
This journal is © The Royal Society of Chemistry 2019

BODIPY as Electron Withdrawing Group for the Activation of Double Bonds in Asymmetric Cycloaddition Reactions

Andrea Guerrero-Corella,[a] Juan Asenjo-Pascual,[a] Tushar Janardan Pawar,[e] Sergio Díaz-Tendero,[b],[c],[d] Ana Martín-Sómer,[b] Clarisa Villegas Gómez,[e] José L. Belmonte-Vázquez,[e] Diana E. Ramírez-Ornelas,[e] Eduardo Peña-Cabrera,[e] Alberto Fraile,[a],[c] David Cruz Cruz,[e]* José Alemán[a],[c]*

[a]Organic Chemistry Department (Módulo 1), Universidad Autónoma de Madrid, 28049-Madrid, Spain.

[b] Chemistry Department (Módulo 13), Universidad Autónoma de Madrid, 28049-Madrid, Spain.

[c] Institute for Advanced Research in Chemical Sciences (IAdChem), Universidad Autónoma de Madrid, 28049-Madrid, Spain.

[d] Condensed Matter Physics Center (IFIMAC), Universidad Autónoma de Madrid, 28049-Madrid, Spain.

[e] Chemistry Department, División de Ciencias Naturales y Exactas Universidad de Guanajuato, Noria Alta S/N 36050 Guanajuato, Gto. México

Supporting Information

Table of Contents

1. General Experimental Details S2
2. Synthesis of BODIPY derivatives **2a** and **2h-i** S3
3. Synthesis of BODIPY derivatives **2j-2k**. S6
4. General procedure C for the organocatalytic [4+2] cycloaddition reaction. S8
5. Synthesis of compound **8** S15
6. X-Ray Structure of compound **8** S16
7. UV-Vis absorption and fluorescence emission spectra of products **5** S16
8. NMR Spectra S17
9. SFC Cromatographs S43
10. Computational Details S54
11. Intrinsic Reaction Coordinate Plots S55
12. Frontier Molecular Orbitals S56
13. References S58

1. General Experimental Details

The solvents employed in the reactions were used without any further purification. The model [4+2] cycloaddition reaction was carried out in vials and stirred with a magnetic bar without inert atmosphere.

NMR spectra were acquired on a Bruker 300 spectrometer, running at 300, 75 and 282 MHz for ^{1}H, ^{13}C and ^{19}F, respectively. Chemical shifts (δ) are reported in ppm relative to residual solvent signals ($CDCl_3$, 7.26 ppm for ^{1}H NMR and 77.00 ppm for ^{13}C NMR). ^{13}C NMR spectra were acquired on a broadband decoupled mode. The following abbreviations are used to describe peak patterns when appropriate: s (singlet), d (doublet), t (triplet), q (quartet), quint (quintet), sept (septuplet), m (multiplet), br (broad).

Analytical thin layer chromatography (TLC) was performed using pre-coated aluminium-backed plates, with fluorescence indicator to 254 nm, and visualized by ultraviolet irradiation and/or by treatment with potassium permanganate. Purification of reaction products was carried out by flash chromatography (FC) using Iatrobeads or silica gel (6RS-8060), indicated each case.

Optical rotations were measured on a Perkin-Elmer 241 polarimeter at room temperature and $[\alpha]^{20}_{D}$ values are given in deg•cm•g^{-1}•dm^{-1}; concentration *c* is listed in g•(100 $mL^{)-1}$. The enantiomeric excess (ee) of the products were determined by SFC using mixtures of supercritical CO_2 and methanol and Chiralpak IA, IB-3, IC, ID, IG-3, OJ-H columns as chiral stationary phases.

High Resolution Mass Spectra (HRMS) were acquired on an Agilent Technologies 5977B MSD using electrospray (ESI) making use of the MassWorks software ver. 4.0.0.0. (Cerno Bioscience) for the formula identification. MassWorks is a MS calibration software, which calibrates for isotope profile as well as for mass accuracy allowing highly accurate comparisons between calibrated and theoretical spectra.[1] Obtained data are expressed in mass/charge (m/z) units.

Commercially available reagents and catalysts were used without further purification. Racemic samples were prepared from a 1:1 mixture of compounds obtained using catalyst (*S*) or (*R*), respectively. Dienals **1a,**[2] **1c**,[3] **1d**,[4] **1e**,[3] **1f**,[3] **1g**,[5] were synthesized following procedures described in the literature.
BP abbreviation in the manuscript means the BODIPY core.
The UV-vis absorption and fluorescence emission spectra of final products **5** dissolved in acetonitrile are shown in Figure 1 (Concentration: from $1{\cdot}10^{-5}$ M to $2{\cdot}10^{-5}$ M).

2. Synthesis of BODIPY derivatives 2a and 2h-i.

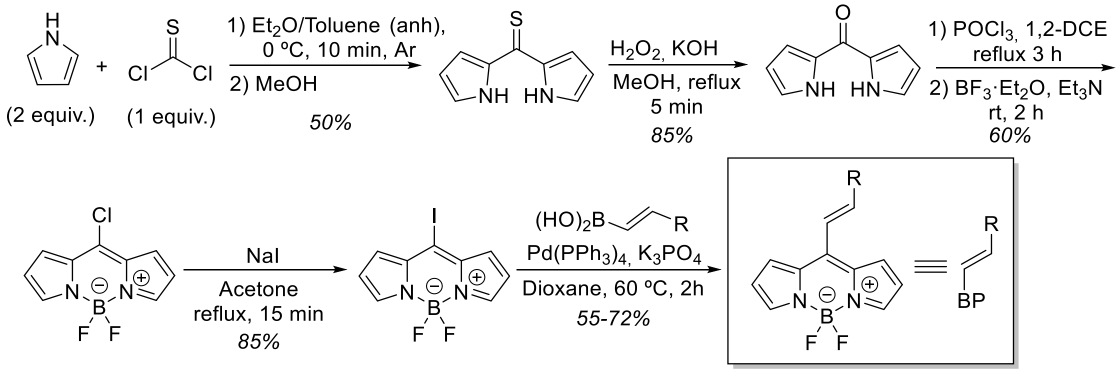

di(1*H*-pyrrol-2-yl)methanethione.[6]

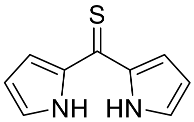

To a stirred solution under argon atmosphere of pyrrole (4.14 mL, 59 mmol, 2 equiv.) in anhydrous diethyl ether (90 mL) at 0 ºC, a solution of thiophosgene (2.25 mL, 29.5 mmol, 1 equiv.) in anhydrous toluene (78 mL) was added dropwise. The mixture was stirred at 0 ºC for 10 minutes. After completion, the reaction mixture reached rt, MeOH was added and the reaction mixture was stirred for 30 min. Then, the solvents were evaporated under reduced pressure and the crude was purified by flash chromatography (eluent: Cy:AcOEt 7:1). The thioketone was obtained as a red solid with a 50% yield. Spectroscopic data are in agreement with the published data.[6]

^{1}H NMR (300 MHz, CDCl$_3$) δ 9.78 (brs, 2H), 7.25 – 7.16 (m, 2H), 7.10 – 7.01 (m, 2H), 6.46 – 6.37 (m, 2H).

di(1*H*-pyrrol-2-yl)methanone.[6]

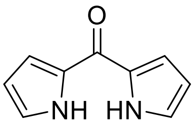

To a stirred solution of the thioketone (2.6 g, 15 mmol, 1 equiv.) in 82 mL of MeOH, KOH (3.25 g, 58 mmol, 4 equiv.) was added and the mixture was stirred for 5 min at 0 °C. Then, H_2O_2 (30%, 11 mL, 67 mmol, 4.5 equiv.) was added dropwise and the reaction crude was reflux. After 5 minutes, the reaction is cooled to room temperature and water (130 mL) is added. Finally, the crude is again cooled to 0 ºC. The solid obtained was filtered obtaining the ketone as a white solid in 85% yield. Spectroscopic data are in agreement with the published data.[6]

^{1}H NMR (300 MHz, CDCl$_3$) δ 9.79 (s, 2H), 7.17 – 7.16 (m, 2H), 7.10 – 7.08 (m, 2H), 6.36 – 6.35 (m, 2H).

10-chloro-5,5-difluoro-5*H*-4λ^4,5λ^4-dipyrrolo[1,2-*c*:2',1'-*f*][1,3,2]diazaborinine.[7]

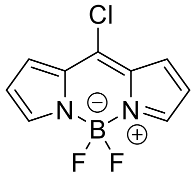

To a solution of the ketone (1.2 g, 7.5 mmol, 1 equiv.) in DCE (40 mL), phosphorus(V) oxychloride (1.5 mL, 15 mmol, 2 equiv.) was added and the reaction mixture was reflux (85 °C) for 3 hours. Then, the crude was cooled to 0 °C and triethylamine (12.5 mL, 75 mmol, 10 equiv.) was added dropwise. After stirring for 5 minutes, boron trifluoride diethyl eterate (12.5 mL, 82.5 mmol, 11 equiv.) was added and the mixture was stirred at rt for 2 hours. After completion, the crude was dissolved in water and extracted with Et_2O. The combined organic layers were dried over magnesium sulfate and concentrated *in vacuum*. The residue was purified by column chromatography on silica gel, eluting with Cy/DCM (1:1) obtaining the final chloride product as a red solid in 60% yield. Spectroscopic data are in agreement with the published data.[7]

^{1}H NMR (300 MHz, $CDCl_3$) δ 7.88 (brs, 2H), 7.41 (d, 2H, *J* = 3.8 Hz), 6.58 (brs, 2H).

5,5-difluoro-10-iodo-5*H*-4λ^4,5λ^4-dipyrrolo[1,2-*c*:2',1'-*f*][1,3,2]diazaborinine.[7]

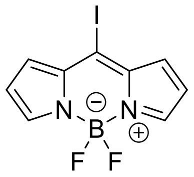

A solution of the chloride product (1.15 g, 3.62 mmol, 1 equiv.) and sodium iodide (3.05 g, 14.5 mmol, 4 equiv.) in acetone (36 mL) under argon atmosphere was refluxed (65 °C) for 15 min. Then, the reaction mixture was left to rise rt and was dissolved in water and extracted with Et_2O. The combined organic layers were dried over magnesium sulfate and concentrated *in vacuum*. The residue was purified by column chromatography on silica gel, eluting with Cy/DCM (1/1) obtaining the final iodide product as a red solid in 85% yield. Spectroscopic data are in agreement with the published data.[7]

^{1}H NMR (300 MHz, $CDCl_3$) δ 7.98 (brs, 2H), 7.29 (d, 2H, *J* = 3.8 Hz), 6.53 (brs, 2H).

General procedure A for the synthesis of ***2a*** *and* ***2h-l*** *by a Suzuki coupling reaction.*[7]

A two-neck round bottom flask equipped with a magnetic stir bar and a reflux condenser, under argon atmosphere, was charged with the corresponding boronic acid (2 equiv.), the iodide compound (1 equiv.) and anhydrous dioxane (24 mL). To this solution, tetrakis(triphenylphosphine)palladium(0) (0.05 equiv.) and K_3PO_4 (3 equiv.) were added, and the mixture was heated at 60 °C for 2 hours. After completion, the solvent was concentrated in vacuum and the residue was purified by column chromatography on silica gel, eluting with Cy/AcOEt (9:1) obtaining the final products indicated each case.

(*E*)-5,5-difluoro-10-styryl-5*H*-4λ^4,5λ^4-dipyrrolo[1,2-*c*:2',1'-*f*][1,3,2]diazaborinine (2a)

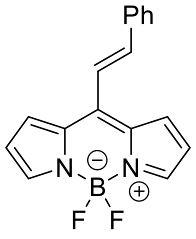

2a

From *trans*-2-phenylvinylboronic acid (493 mg, 3.34 mmol, 2 equiv.), iodide compound (530 mg, 1.67 mmol, 1 equiv.), tetrakis(triphenylphosphine)palladium(0) (96.3 mg, 0.08 mmol, 0.05 equiv.) and K_3PO_4 (1.061 g, 5.00 mmol, 3 equiv.), following *general procedure A*, compound **2a** was obtained in 60% yield as a purple solid. Spectroscopic data are in agreement with the published data.[8]

^{1}H NMR (300 MHz, $CDCl_3$) δ 7.89 (brs, 2H), 7.65 – 6.59 (m, 2H), 7.50 – 7.32 (m, 7H), 6.56 (brs, 2H).

(*E*)-5,5-difluoro-10-(4-(trifluoromethyl)styryl)-5H-4λ^4,5λ^4-dipyrrolo[1,2-*c*:2',1'-*f*] [1,3,2]diazaborinine (2h)

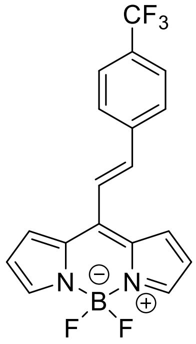

2h

From *trans*-2-[4-(Trifluoromethyl)phenyl]vinylboronic acid (271.7 mg, 1.26 mmol, 2 equiv.), iodide compound (200.0 mg, 0.63 mmol, 1 equiv.), tetrakis(triphenylphosphine)palladium(0) (36.4 mg, 0.03 mmol, 0.05 equiv.) and K_3PO_4 (400.6 mg, 1.89 mmol, 3 equiv.), following *general procedure A*, compound **2h** was obtained in 72% yield as a red solid. Spectroscopic data are in agreement with the published.[9]

^{1}H NMR (300 MHz, $CDCl_3$) δ 7.92 (brs, 2H), 7.72 (brs, 4H), 7.48 (brs, 2H), 7.35 (d, *J* = 4.3 Hz, 2H) 6.58 (d, *J* = 3.7 Hz, 2H).

(*E*)-10-(4-chlorostyryl)-5,5-difluoro-5H-4λ^4,5λ^4-dipyrrolo[1,2-*c*:2',1'-*f*][1,3,2] diazaborinine (2i)

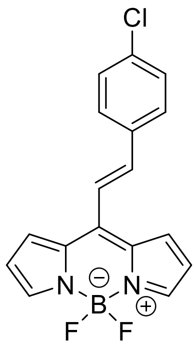

2i

From *trans*-2-(4-Chlorophenyl)vinylboronic acid (229.8 mg, 1.26 mmol, 2 equiv.), iodide compound (200.0 mg, 0.63 mmol, 1 equiv.), tetrakis(triphenylphosphine)palladium(0) (36.4 mg, 0.03 mmol, 0.05 equiv.) and K_3PO_4 (400.6 mg, 1.89 mmol, 3 equiv.) following *general procedure A*, compound **2i** was obtained in 55% yield as a red solid. Spectroscopic data are in agreement with the published.[9]

^{1}H NMR (300 MHz, $CDCl_3$) δ 7.88 (brs, 2H), 7.64 – 7.57 (m, 2H), 7.49 – 7.40 (m, 4H), 7.34 (d, *J* = 4.3 Hz, 2H), 6.57 – 6.51 (m, 2H).

3. Synthesis of BODIPY derivatives 2j-2k.

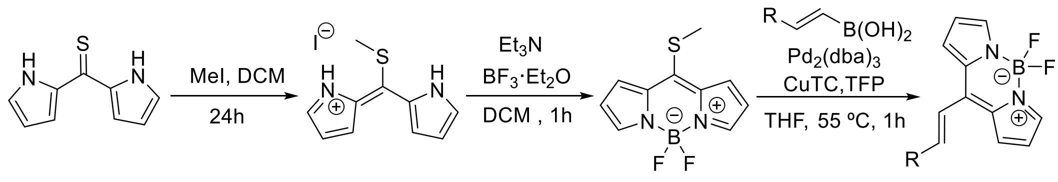

(*E*)-2-((methylthio)(1*H*-pyrrol-2-yl)methylene)-2*H*-pyrrol-1-ium iodide.[10]

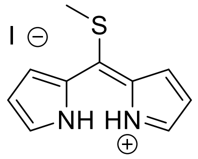

To a stirred solution of the thioketone (2.60 g, 14.86 mmol, 1 equiv.) in anhydrous DCM (50 mL), MeI (4.8 mL, 74.3 mmol, 5.8 equiv.) was added and the mixture was stirred at room temperature for 24h. Then, the solvent and the MeI in excess were removed under reduced pressure obtaining the iodide thioether as a black solid (4.73 g) in quantitative yield. Spectroscopic data are in agreement with the published data[10] and the product was used without further purification.

^{1}H NMR (300 MHz, $CDCl_3$) δ 12.0 (brs, 2H), 7.91 – 89 (m, 2H), 7.29 – 7.26 (m, 2H), 6.68 – 6.66 (m, 2H), 2.91 (s, 3H).

5,5-difluoro-10-(methylthio)-5*H*-4λ^4,5λ^4-dipyrrolo[1,2-*c*:2',1'-*f*][1,3,2] diazaborinine.[10]

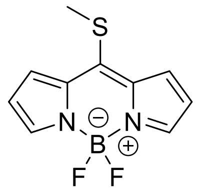

To a stirred solution under argon atmosphere of the iodide thioether (450 mg, 1.4 mmol, 1 equiv.) in anhydrous DCM (11 mL), triethylamine (0.35 mL, 4.76 mmol, 3.5 equiv.) was added and the mixture was stirred at room temperature for 30 minutes. Then, the solution was cooled to 0 ºC and $BF_3\cdot Et_2O$ (0.9 mL, 7.3 mmol, 5 equiv.) was added dropwise. The reaction mixture was led to reach room temperature and was stirred for an additional 30 minutes. The solvent was removed under reduced pressure and the crude was purified by column chromatography on silica gel, eluting with Cy/AcOEt (3:1) obtaining the final product as a red solid (85 mg, 25% yield). Spectroscopic data are in agreement with the published data.[10]

^{1}H NMR (300 MHz, $CDCl_3$) δ 7.80 (brs, 2H), 7.43 – 7.41 (m, 2H), 6.54 – 6.52 (m, 2H), 2.92 (s, 3H).

General procedure B for the synthesis of ***2j-k*** *by a Liebeskind-Srogl coupling reaction.*[9]

A two-neck round bottom flask equipped with a magnetic stir bar and a reflux condenser, under argon atmosphere, was charged with the corresponding boronic acid (3 equiv.)

and thioether compound (1 equiv.) in anhydrous THF (10 mL). To this solution, copper thiophene-2-carboxylate (3 equiv.), $Pd_2(dba)_3$ (0.025 equiv.) and tri(2-furyl)phosphine (0.075 equiv.) were added and the mixture was heated at 55 °C for 24 hours. After completion, the solvent was concentrated in vacuum and the residue was purified by column chromatography on silica gel, eluent indicated each case.

(*E*)-5,5-difluoro-10-(4-methoxystyryl)-5H-4λ^4,5λ^4-dipyrrolo[1,2-*c*:2',1'-*f*][1,3,2] diazaborinine (2j)

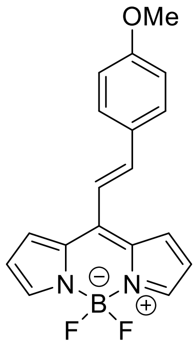

2j

From *trans*-2-(4-methoxyphenyl)vinylboronic acid (326 mg, 1.83 mmol, 3 equiv.), thioether compound (145 mg, 0.61 mmol, 1 equiv.), copper thiophene-2-carboxylate (349 mg, 1.83 mmol, 3 equiv.), $Pd_2(dba)_3$ (13.7 mg, 0.015 mmol, 0.025 equiv.) and tri(2-furyl)phosphine (10.7 mg, 0.046 mmol, 0.075 equiv.), following *general procedure B*, compound **2j** was obtained in 40% yield as a red solid. The crude product was purified by flash column chromatography (gradient Cy/AcOEt from 9:1 to 5:1). Spectroscopic data are in agreement with the published data.[9]

^{1}H NMR (300 MHz, $CDCl_3$) δ 7.87 (brs, 2H), 7.61 – 7.49 (m, 3H), 7.38 – 7.31 (m, 3H), 7.02 – 6.94 (m, 2H), 6.57 – 6.52 (m, 2H), 3.89 (s, 3H).

(*E*)-5,5-difluoro-10-(oct-1-en-1-yl)-5H-4λ^4,5λ^4-dipyrrolo[1,2-*c*:2',1'-*f*][1,3,2] diazaborinine (2k)

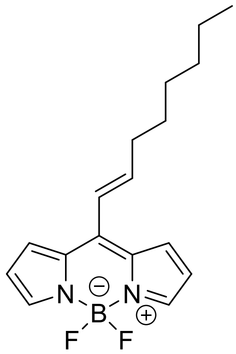

2k

From *trans*-1-octenylboronic acid (98.3 mg, 0.63 mmol, 3 equiv.), thioether compound (50.0 mg, 0.21 mmol, 1 equiv.), copper thiophene-2-carboxylate (120.2 mg, 0.63 mmol, 3 equiv.), $Pd_2(dba)_3$ (4.8 mg, 0.005 mmol, 0.025 equiv.) and tri(2-furyl)phosphine (3.7 mg, 0.016 mmol, 0.075 equiv.), following *general procedure B*, compound **2k** was obtained in 90% yield as a red oil. The crude product was purified by flash column chromatography (gradient pentane/AcOEt from 9:1 to 5:1).

^{1}H NMR (300 MHz, $CDCl_3$) δ 7.85 (brs, 2H), 7.25 (d, *J* = 4.1 Hz, 2H), 6.85 – 6.61 (m, 2H), 6.59 – 6.50 (m, 2H), 2.39 (td, *J* = 7.3, 5.9 Hz, 2H), 1.66 – 1.51 (m, 2H), 1.48 – 1.18 (m, 6H), 0.98 – 0.81 (m, 3H). **^{13}C NMR (75 MHz, $CDCl_3$)**: δ 149.4, 144.3, 142.9, 133.7, 128.5, 123.2, 117.7, 34.3, 31.6, 28.9, 28.6, 22.6, 14.1. **^{19}F NMR (282 MHz, $CDCl_3$)** δ -145.95 (dd, *J* = 57.2, 28.6 Hz, 2F). **HRMS (ESI$^+$)** calculated for $C_{17}H_{23}N_2BF_2$ $[M+H]^+$: 303.1953, found: 303.1930.

4. General procedure C for the organocatalytic [4+2] cycloaddition reaction.

A dry vial equipped with a magnetic stir bar was charged with the corresponding aminocatalyst **3** (0.01 mmol, 0.1 equiv.), PhCOOH (0.01 mmol, 0.1 equiv.) and the corresponding dienal (0.25 mmol, 2.5 equiv.). *p*-Xylene (1 mL) was added to dissolve the compounds, there upon the corresponding BODIPY (0.1 mmol, 1 equiv.) was added to the mixture. The reaction mixture was stirred at 45 °C for the time indicated in each case. After completion, full conversion was determined by ^{1}H NMR, *p*-xylene (1 mL) and (methoxycarbonylmethylene)triphenylphosphorane (0.25 mmol, 2.5 equiv.) were added to derivatize to the final products **5**. The crude product was purified by flash column chromatography on silica gel (eluent indicated in each case).

Methyl (*E*)-4-((1*S*,2*S*,3*R*)-2-(5,5-difluoro-5*H*-4λ^{4},5λ^{4}-dipyrrolo[1,2-*c*:2',1'-*f*][1,3,2]diazaborinin-10-yl)-5-methyl-1,2,3,6-tetrahydro-[1,1'-biphenyl]-3-yl)but-2-enoate (5a)

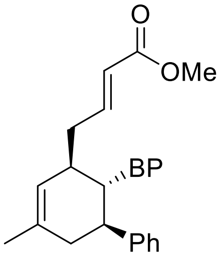

5a

From **1a** (27.5 mg, 0.25 mmol) and BODIPY **2a** (29.4 mg, 0.1 mmol), following the general procedure C (45 ºC, 18h), compound **5a** (35.9 mg, 0.078 mmol) was obtained in 78% yield as a red solid. The crude product was purified by flash column chromatography (gradient Cy/AcOEt from 9:1 to 5:1). The *ee* was determined by SFC using Chiralpak IC column [CO_2/MeOH (90:10), 120 bar, 40 °C)]; 3.0 mL/min. τ_{may} = 6.994 min, τ_{min} = 7.535 min, *ee*= 96%. **$[\alpha]^{20}_{D}$** = +945 (*c* 0.031, CH_2Cl_2).

^{1}H NMR (300 MHz, $CDCl_3$) δ 7.72 (brs, 2H), 7.53 (brd, *J* = 4.4 Hz, 1H), 7.21 – 7.17 (m, 1H), 7.10 – 6.78 (m, 6H), 6.56 (brd, *J* = 4.2 Hz, 1H), 6.40 (brd, *J* = 4.4 Hz, 1H), 5.70 (d, *J* = 15.5 Hz, 1H), 5.48 (brs, 1H), 3.73 (s, 3H), 3.61 – 3.47 (m, 1H), 3.22 – 3.13 (m, 2H), 2.53 – 2.27 (m, 3H), 2.17 – 2.02 (m, 1H), 1.82 (s, 3H). **^{13}C NMR (75 MHz, $CDCl_3$)** δ 166.6, 152.3, 145.6, 144.6, 141.8, 141.7, 137.3, 135.6, 133.0, 129.6, 128.4, 128.2 (2C), 127.4 (2C), 126.9, 123.6, 123.1, 117.9, 117.6, 51.5, 50.5, 48.8, 43.8, 39.7, 37.1, 23.3. **^{19}F NMR (282 MHz, $CDCl_3$)** δ -145.09 (ddd, *J* = 105.7, 58.4, 29.4 Hz, 1F), -147.68 (ddd, *J* = 105.1, 56.0, 27.7 Hz, 1F). **HRMS (ESI^{+})** calculated for $C_{27}H_{31}N_3O_2BF_2$ $[M+NH_4]^{+}$: 478.2586, found: 478.2566.

Methyl (*E*)-4-((1*S*,2*S*,3*R*)-2-(5,5-difluoro-5*H*-4λ^4,5λ^4-dipyrrolo[1,2-*c*:2',1'-*f*][1,3,2] diazaborinin-10-yl)-1,2,3,6-tetrahydro-[1,1'-biphenyl]-3-yl)but-2-enoate (5b).

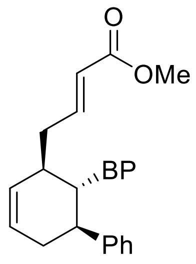

5b

From **1b** (28 µL, 0.25 mmol) and BODIPY **2a** (29.4 mg, 0.1 mmol), following the general procedure C (45 ºC, 48h), compound **5b** (32.1 mg, 0.072 mmol) was obtained in 72% yield as a red solid. The crude product was purified by flash column chromatography (gradient Cy/AcOEt from 9:1 to 5:1). The *ee* was determined by SFC using Chiralpak IA column [CO_2/MeOH (90:10), 120 bar, 40 °C]; 3.0 mL/min. τ_{may} = 9.547 min, τ_{min} = 9.088 min, *ee*=92% **$[\alpha]^{20}_D$** = -47.6 (*c* 0.043, CH_2Cl_2).

^{1}H NMR (300 MHz, $CDCl_3$) δ 7.71 (s, 2H), 7.53 (d, *J* = 4.3 Hz, 1H), 7.19 (d, *J* = 4.4 Hz, 1H), 7.06 – 6.99 (m, 3H), 6.92 – 6.79 (m, 3H), 6.59 – 6.52 (m, 1H), 6.42 – 6.35 (m, 1H), 6.04 – 6.00 (m, 1H), 5.78 (d, *J* = 10.4 Hz, 1H), 5.71 (d, *J* = 15.7 Hz, 1H), 3.71 (s, 3H), 3.59 – 3.43 (m, 1H), 3.28 – 3.21 (m, 2H), 2.50 – 2.46 (m, 2H), 2.40 – 2.32 (m, 1H), 2.17 – 2.06 (m, 1H). **^{13}C NMR (75 MHz, $CDCl_3$)** δ 166.4, 151.9, 145.2, 144.7, 141.9, 141.6, 137.3, 133.0, 129.6, 128.8, 128.4, 128.2 (2C), 128.0, 127.5 (2C), 126.9, 123.8, 118.0, 117.7, 51.5, 50.5, 48.3, 43.6, 36.7, 34.6. **^{19}F NMR (282 MHz, $CDCl_3$)** δ -145.07 (ddd, J = 105.4, 58.3, 29.1 Hz, 1F), -147.70 (ddd, *J* = 105.5, 56.0, 28.0 Hz, 1F). **HRMS (ESI$^+$)** calculated for $C_{26}H_{29}BF_2N_3O_2$ [M + NH_4^+] = 464.2430, found: 464.2451.

Methyl (*E*)-4-((2'*S*,3'*R*,4'*S*)-3'-(5,5-difluoro-5*H*-4λ^4,5λ^4-dipyrrolo[1,2-*c*:2',1'-*f*][1,3,2] diazaborinin-10-yl)-2',3',4',5'-tetrahydro-[1,1':4',1''-terphenyl]-2'-yl)but-2-enoate (5c).

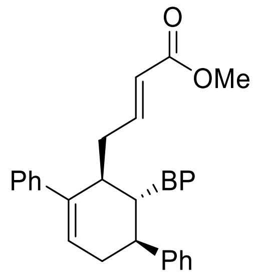

5c

From **1c** (43 mg, 0.25 mmol) and BODIPY **2a** (29.4 mg, 0.1 mmol), following the general procedure C (45 ºC, 18h), compound **5c** (40.7 mg, 0.078 mmol) was obtained in 78% yield as a red solid. The crude product was purified by flash column chromatography (gradient Cy/AcOEt from 9:1 to 5:1). The *ee* was determined by SFC using Chiralpak IC column [CO_2/MeOH (90:10), 120 bar, 40 °C)]; 3.0 mL/min. τ_{may} = 8.794 min, τ_{min} = 11.848 min, *ee*=95% **$[\alpha]^{20}_D$** = +1096 (*c* 0.031, CH_2Cl_2).

^{1}H NMR (300 MHz, $CDCl_3$) δ 7.76 (s, 1H), 7.72 (s, 1H), 7.63 (d, *J* = 4.3 Hz, 1H), 7.41 – 7.20 (m, 6H), 7.11 – 6.98 (m, 4H), 6.96 – 6.89 (m, 2H), 6.76 (ddd, *J* = 15.1, 8.9, 5.7 Hz, 1H), 6.63 (dd, *J* = 4.3, 1.9 Hz, 1H), 6.35 (dd, *J* = 4.4, 1.8 Hz, 1H), 6.31 (m, 1H), 5.56 (d, *J* = 15.5 Hz, 1H), 3.92 – 3.80 (m, 1H), 3.72 (s, 3H), 3.60 – 3.46 (m, 1H), 3.40 (dd, *J* =

11.9, 8.9 Hz, 1H), 2.67 – 2.57 (m, 2H), 2.44 – 2.31 (m, 1H), 2.29 – 2.15 (m, 1H). **^{13}C NMR (75 MHz, $CDCl_3$)** δ 166.4, 152.5, 145.3, 144.2, 141.6, 141.5, 140.6, 139.6, 137.0, 133.0, 130.0, 128.7 (2C), 128.33, 128.29 (2C), 127.9, 127.4, 127.3 (2C), 127.0, 126.3 (2C), 124.3, 118.1, 117.7, 51.5, 50.5, 48.8, 44.7, 34.3, 33.7. **^{19}F NMR (282 MHz, $CDCl_3$)** δ -144.44 (ddd, *J* = 105.1, 58.5, 29.1 Hz, 1F), -148.03 (ddd, *J* = 105.1, 55.6, 27.7 Hz, 1F). **HRMS (ESI$^+$)** calculated for $C_{32}H_{29}N_2O_2BF_2$ $[M+NH_4]^+$: 540.2743, found: 540.2692.

Methyl (*E*)-4-((1'*S*,5'*R*,6'*S*)-6'-(5,5-difluoro-5*H*-4λ^4,5λ^4-dipyrrolo[1,2-*c*:2',1'-*f*][1,3,2]diazaborinin-10-yl)-1',2',5',6'-tetrahydro-[1,1':3',1''-terphenyl]-5'-yl)but-2-enoate (5d)

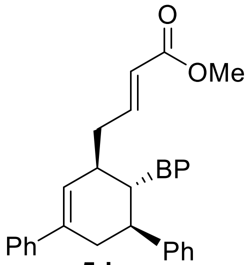

5d

From **1d** (21.5 mg, 0.12 mmol) and BODIPY **2a** (14 mg, 0.048 mmol), following the general procedure C (45 ºC, 18h), compound **5d** (20.0 mg, 0.038 mmol) was obtained in 80% yield as a red solid. The crude product was purified by flash column chromatography (gradient Cy/AcOEt from 9:1 to 5:1). The *ee* was determined by SFC using Chiralpak IB-3 column [CO_2/MeOH (90:10), 120 bar, 40 °C)]; 2.0 mL/min. τ_{may} = 5.816 min, τ_{min} = 6.623 min, *ee*= 95 %. **$[\alpha]^{20}_D$** = -10.3 (*c* 0.205, CH_2Cl_2).

^{1}H NMR (300 MHz, $CDCl_3$) δ 7.74 (s, 2H), 7.58 (d, *J* = 4.3 Hz, 1H), 7.46 – 7.39 (m, 2H), 7.40 – 7.27 (m, 3H), 7.24 (d, *J* = 4.5 Hz, 1H), 7.13 – 7.02 (m, 3H), 7.02 – 6.95 (m, 2H), 6.89 (ddd, *J* = 15.1, 8.9, 5.6 Hz, 1H), 6.58 (dd, *J* = 4.3, 1.9 Hz, 1H), 6.42 (dd, *J* = 4.4, 1.8 Hz, 1H), 6.18 – 6.11 (m, 1H), 5.75 (d, *J* = 15.7, 1H), 3.72 (s, 3H), 3.76 – 3.62 (m, 1H), 3.46 – 3.28 (m, 2H), 2.97 – 2.83 (m, 2H), 2.55 – 2.39 (m, 1H), 2.30 – 2.13 (m, 1H) **^{13}C NMR (75 MHz, $CDCl_3$)** δ 166.4, 151.8, 145.2, 144.8, 142.0, 141.4, 140.5, 138.2, 137.4, 132.9, 129.6, 128.5, 128.4, 128.3, 127.7, 127.5, 127.1, 125.5, 125.3, 124.0, 118.1, 117.7, 51.5, 50.2, 48.8, 44.4, 37.3, 37.1. **^{19}F NMR (282 MHz, $CDCl_3$)** δ -145.00 (ddd, *J* = 105.3, 58.1, 28.9 Hz, 1F), -147.62 (ddd, *J* = 105.3, 56.0, 28.0 Hz, 1F). **HRMS (ESI$^+$)** calculated for $C_{32}H_{34}N_3O_2BF_2$ $[M+NH_4]^+$: 540.2743, found: 540.2680.

Methyl (*E*)-4-((2'*S*,3'*S*,4'*S*,5'*R*)-3'-(5,5-difluoro-5*H*-4λ^4,5λ^4-dipyrrolo[1,2-*c*:2',1'-*f*][1,3,2]diazaborinin-10-yl)-5'-methyl-2',3',4',5'-tetrahydro-[1,1':4',1''-terphenyl]-2'-yl)but-2-enoate (5e).

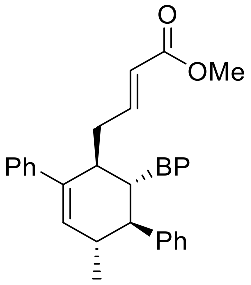

5e

From **1e** (46.5 mg, 0.25 mmol) and BODIPY **2a** (29.4 mg, 0.1 mmol), following the general procedure C (45 ºC, 48h), compound **5e** (49.4 mg, 0.092 mmol) was obtained in 92% yield as a red solid. The crude product was purified by flash column chromatography (gradient Cy/AcOEt from 9:1 to 5:1). The *ee* was determined by SFC using Chiralpak IB-3 column [CO_2/MeOH (90:10), 120 bar, 40 °C]; 2.0 mL/min. τ_{may} = 2.007 min, τ_{min} = 3.194 min, *ee*=98% **[α]$^{20}_{D}$** = -282 (*c* 0.023, CH_2Cl_2).

^{1}H NMR (300 MHz, CDCl$_3$) δ 7.82 (s, 1H), 7.68 (s, 1H), 7.61 (d, *J* = 4.3 Hz, 1H), 7.47 (d, *J* = 4.3 Hz, 1H), 7.40 – 7.28 (m, 6H), 7.11 – 7.04 (m, 3H), 7.04 – 6.93 (m, 2H), 6.80 (ddd, *J* = 15.6, 8.9, 5.5 Hz, 1H), 6.60 – 6.52 (m, 2H), 6.32 (dd, *J* = 6.3, 1.6 Hz, 1H), 5.60 (dt, *J* = 15.8, 1.5 Hz, 1H), 3.91 – 3.82 (m, 2H), 3.74 (s, 3H), 2.72 – 2.59 (m, 1H), 2.44 – 2.30 (m, 1H), 2.30 – 2.16 (m, 1H), 1.07 (d, *J* = 7.1 Hz, 3H). **^{13}C NMR (75 MHz, CDCl$_3$)** δ 166.3, 152.8, 144.8, 144.4, 142.0, 140.7, 139.6, 138.2, 137.5, 135.0, 133.5, 129.5, 129.0 (2C), 128.7 (2C), 128.3, 127.9 (2C), 127.4, 126.8, 126.5 (2C), 124.2, 118.3, 118.0, 51.52, 51.49, 45.6, 42.0, 37.2, 33.5, 15.3. **^{19}F NMR (282 MHz, CDCl$_3$)** δ -144.64 (ddd, *J* = 105.6, 58.5, 29.2 Hz, 1F), -147.64 (ddd, *J* = 105.7, 55.7, 27.9 Hz, 1F). **HRMS (ESI$^+$)** calculated for $C_{33}H_{35}N_3O_2BF_2$ [M+NH$_4$]$^+$: 554.2899, found: 554.2894.

Methyl (*E*)-4-((1*S*,2*S*,3*S*,6*R*)-2-(5,5-difluoro-5*H*-4λ^4,5λ^4-dipyrrolo[1,2-*c*:2',1'-*f*][1,3,2]diazaborinin-10-yl)-4,6-dimethyl-1,2,3,6-tetrahydro-[1,1'-biphenyl]-3-yl)but-2-enoate (5f).

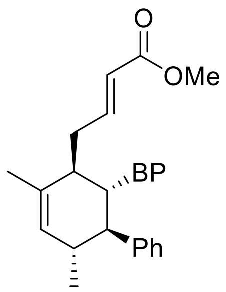

5f

From **1f** (31 mg, 0.25 mmol) and BODIPY **2a** (29.4 mg, 0.1 mmol), following the general procedure C (45 ºC, 48h), compound **5f** (37.0 mg, 0.078 mmol) was obtained in 78% yield as a red solid. The crude product was purified by flash column chromatography (gradient Cy/AcOEt from 9:1 to 5:1). The *ee* was determined by SFC using Chiralpak IA column [CO_2/MeOH (95:5), 120 bar, 40 °C]; 3.0 mL/min. τ_{may} = 8.013 min, τ_{min} = 6.898 min, *ee*=93% **[α]$^{20}_{D}$** = -221 (*c* 0.019, CH_2Cl_2).

^{1}H NMR (300 MHz, CDCl$_3$) δ 7.80 (s, 1H), 7.63 (s, 1H), 7.44 (dd, *J* = 14.9, 4.4 Hz, 2H), 7.08 – 6.98 (m, 4H), 6.98 – 6.90 (m, 2H), 6.90 – 6.80 (m, 1H), 6.58 – 6.52 (m, 1H), 6.51

– 6.44 (m, 1H), 5.92 (d, *J* = 6.0 Hz, 1H), 5.89 – 5.75 (m, 1H), 3.77 (s, 3H), 3.76 – 3.70 (m, 2H), 3.07 (brs, 1H), 2.59 (dtd, *J* = 16.2, 5.0, 2.3 Hz, 1H), 2.49 – 2.36 (m, 1H), 2.28 (ddd, *J* = 16.4, 8.5, 3.2 Hz, 1H), 1.80 (s, 3H), 0.93 (d, *J* = 7.1 Hz, 3H). **^{13}C NMR (75 MHz, $CDCl_3$)** δ 166.4, 153.0, 144.4 (2C), 141.9, 139.9, 137.6, 133.3, 132.4, 132.1, 129.4, 129.0, 128.6, 127.8, 126.5, 124.5, 118.1, 117.7, 51.8, 51.6, 47.6, 42.0, 37.0, 32.2, 21.4, 15.5. **^{19}F NMR (282 MHz, $CDCl_3$)** δ -144.61 (ddd, *J* = 105.3, 58.2, 29.3 Hz, 1F), -147.78 (ddd, *J* = 106.5, 55.0, 27.9 Hz, 1F). **HRMS (ESI$^+$)** calculated for $C_{28}H_{33}N_3BF_2O_2$ $[M+NH_4]^+$: 492.2743, found: 492.2767.

***tert*-Butyl (2*S*,3*R*,4*S*)-3-(5,5-difluoro-5*H*-4λ^4,5λ^4-dipyrrolo[1,2-*c*:2',1'-*f*][1,3,2] diazaborinin-10-yl)-4-((*E*)-4-methoxy-4-oxobut-2-en-1-yl)-2-phenyl-1,2,3,4-tetrahydro-9*H*-carbazole-9-carboxylate (5g).**

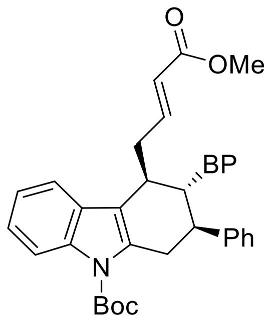

5g

From **1g** (71.3 mg, 0.25 mmol) and BODIPY **2a** (29.4 mg, 0.1 mmol), following the general procedure C (45 ºC, 18h), compound **5g** (42.6 mg, 0.067 mmol) was obtained in 67% yield as a red solid. The crude product was purified by flash column chromatography (gradient Cy/AcOEt from 9:1 to 5:1). The *ee* was determined by SFC using Chiralpak ID column [CO_2/MeOH gradient (from 5% to 40% of MeOH), 120 bar, 40 °C], 2.0 mL/min τ_{may} = 3.652 min, τ_{min} = 3.384, *ee*=82%. **$[\alpha]^{20}_D$** = +280 (*c* 0.051, CH_2Cl_2)

^{1}H NMR (300 MHz, $CDCl_3$) δ 8.20 (d, *J* = 8.3 Hz, 1H), 7.74 (s, 2H), 7.49 (d, *J* = 7.4 Hz, 1H), 7.46 (d, *J* = 4.2 Hz, 1H), 7.37 – 7.30 (m, 2H), 7.29 – 7.22 (m, 2H), 7.13 (d, *J* = 4.4 Hz, 1H), 7.11 – 7.02 (m, 3H), 7.00 – 6.92 (m, 2H), 6.64 (ddd, *J* = 15.6, 9.1, 5.3 Hz, 1H), 6.54 (dd, *J* = 4.3, 1.9 Hz, 1H), 6.40 (dd, *J* = 4.4, 1.9 Hz, 1H), 5.67 (d, *J* = 15.9 Hz, 1H), 4.18 – 4.07 (m, 1H), 3.67 (s, 3H), 3.71 – 3.45 (m, 3H), 3.36 (ddd, *J* = 18.8, 11.9, 2.4 Hz, 1H), 3.09 (dtd, *J* = 15.5, 5.4, 2.0 Hz, 1H), 2.69 – 2.56 (m, 1H), 1.68 (s, 9H). **^{13}C NMR (75 MHz, $CDCl_3$)** δ 166.3, 151.7, 150.3, 145.2, 143.8, 141.7, 140.8, 137.2, 136.5, 136.3, 133.0, 130.1, 128.2 (2C), 127.8, 127.5 (2C), 127.1, 124.7, 124.0, 122.8, 118.6, 118.1, 117.6, 116.0, 115.8, 84.3, 51.5, 50.5, 49.6, 40.8, 34.9, 34.1, 28.2. **^{19}F NMR (282 MHz, $CDCl_3$)** δ -144.81 (ddd, *J* = 104.8, 58.4, 29.2 Hz, 1F), -147.75 (ddd, *J* = 105.1, 55.7, 27.7 Hz, 1F). **HRMS (ESI$^+$)** calculated for $C_{37}H_{40}BF_2N_4O_4$ [M + NH_4^+] = 653.3220, found: 653.3278.

Methyl (*E*)-4-((1*S*,2*S*,3*R*)-2-(5,5-difluoro-5*H*-4λ⁴,5λ⁴-dipyrrolo[1,2-*c*:2',1'-*f*][1,3,2] diazaborinin-10-yl)-5-methyl-4'-(trifluoromethyl)-1,2,3,6-tetrahydro-[1,1'-biphenyl]-3-yl)but-2-enoate (5h)

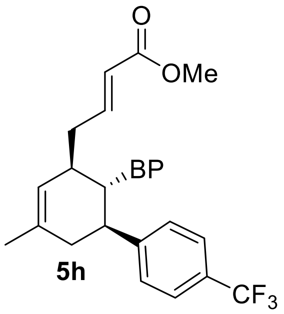

From **1a** (27.5 mg, 0.25 mmol) and BODIPY **2h** (36.2 mg, 0.1 mmol), following the general procedure C (45 ºC, 18h), compound **5h** (40.2 mg, 0.076 mmol) was obtained in 76% yield as a red solid. The crude product was purified by flash column chromatography (gradient Cy/AcOEt from 9:1 to 5:1). The *ee* was determined by SFC using Chiralpak OJ-H column [CO_2/MeOH (90:10), 120 bar, 40 °C)]; 3.0 mL/min. τ_{may} = 2.432 min, τ_{min} = 2.195 min, *ee*= 94 %. **$[\alpha]^{20}_D$** = +5722 (*c* 0.005, CH_2Cl_2).

^{1}H NMR (300 MHz, $CDCl_3$) δ 7.73 (d, *J* = 5.6 Hz, 2H), 7.51 (d, *J* = 4.2 Hz, 1H), 7.31 (d, *J* = 8.1 Hz, 2H), 7.19 (d, *J* = 4.3 Hz, 1H), 7.04 (d, *J* = 8.0 Hz, 2H), 6.83 (ddd, *J* = 14.9, 8.9, 5.7 Hz, 1H), 6.56 (dd, *J* = 4.5, 2.0 Hz, 1H), 6.42 (dd, *J* = 4.6, 2.0 Hz, 1H), 5.70 (d, *J* = 15.7 Hz, 1H), 5.50 (s, 1H), 3.71 (s, 3H), 3.68 – 3.51 (m, 1H), 3.26 – 3.11 (m, 2H), 2.50 – 2.23 (m, 3H), 2.16 – 2.02 (m, 1H), 1.81 (s, 3H). **^{13}C NMR (75 MHz, $CDCl_3$)** δ 166.5, 151.3, 145.9 (q, $J_{C\text{-}F}$ = 1.1 Hz), 145.3, 145.1, 142.2, 137.2, 135.2, 132.8, 129.4, 129.1 (q, $J_{C\text{-}F}$ = 32.5 Hz), 128.2, 127.8 (2C), 125.2 (q, $J_{C\text{-}F}$ = 3.7 Hz), 123.9 (q, $J_{C\text{-}F}$ = 272.0 Hz), 123.8, 123.3, 118.3, 117.8, 51.5, 49.9, 48.5, 43.7, 39.7, 36.9, 23.3. **^{19}F NMR (282 MHz, $CDCl_3$)** δ -62.6 (s, CF_3), -144.95 (ddd, *J* = 104.9, 58.2, 29.0 Hz, 1F), -147.79 (ddd, *J* = 104.9, 55.6, 27.9 Hz, 1F). **HRMS (ESI$^+$)** calculated for $C_{28}H_{30}BF_5N_3O_2$ [M + NH_4^+] = 546.2460, found: 546.2411.

Methyl (*E*)-4-((1*S*,2*S*,3*R*)-4'-chloro-2-(5,5-difluoro-5*H*-4λ⁴,5λ⁴-dipyrrolo[1,2-*c*:2',1'-*f*] [1,3,2]diazaborinin-10-yl)-5-methyl-1,2,3,6-tetrahydro-[1,1'-biphenyl]-3-yl)but-2-enoate (5i).

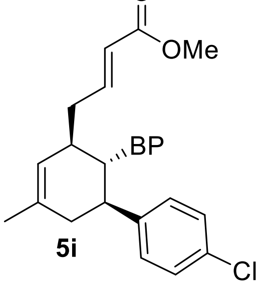

From **1a** (27.5 mg, 0.25 mmol) and BODIPY **2i** (32.9 mg, 0.1 mmol), following the general procedure C (45 ºC, 48h), compound **5i** (30.7 mg, 0.062 mmol) was obtained in 62% yield as a red solid. The crude product was purified by flash column chromatography (gradient Cy/AcOEt from 9:1 to 5:1). The *ee* was determined by SFC using Chiralpak OJ-H column [CO_2/MeOH (95:5), 120 bar, 40 °C)]; 3.0 mL/min. τ_{may} = 8.685 min, τ_{min} = 6.575 min, *ee*= 95 %. **$[\alpha]^{20}_D$** = +1170 (*c* 0.025, CH_2Cl_2).

^{1}H NMR (300 MHz, $CDCl_3$) δ 7.74 (d, *J* = 8.7 Hz, 2H), 7.50 (d, *J* = 4.3 Hz, 1H), 7.18 (d, *J* = 4.5 Hz, 1H), 7.02 and 6.85 (AA'BB' system, 4H), 6.89 – 6.76 (m, 1H), 6.55 (dd, *J* = 4.3, 2.0 Hz, 1H), 6.42 (dd, *J* = 4.4, 1.9 Hz, 1H), 5.69 (d, *J* = 15.7, 1H), 5.48 (s, 1H), 3.72 (s, 3H), 3.58 – 3.44 (m, 1H), 3.22 – 3.06 (m, 2H), 2.48 – 2.22 (m, 3H), 2.14 – 1.95 (m, 1H), 1.80 (s, 3H). **^{13}C NMR (75 MHz, $CDCl_3$)** δ 166.5, 151.7, 145.4, 145.0, 142.1, 140.3, 137.2, 135.4, 132.8, 132.5, 129.4, 128.7, 128.4, 128.3, 123.7, 123.3, 118.2, 117.7, 51.5, 50.2, 48.1, 43.8, 39.8, 37.0, 23.3. **^{19}F NMR (282 MHz, $CDCl_3$)** δ -144.80 (ddd, *J* = 104.3, 58.8, 29.3 Hz, 1F), -147.72 (ddd, *J* = 104.8, 55.2, 27.5 Hz, 1F). **HRMS (ESI$^+$)** calculated for $C_{27}H_{30}BF_2N_3O_2Cl$ [M + NH_4^+] = 512.2197, found: 512.2175.

Methyl (*E*)-4-((1*S*,2*S*,3*R*)-2-(5,5-difluoro-5*H*-4λ^4,5λ^4-dipyrrolo[1,2-*c*:2',1'-*f*][1,3,2] diazaborinin-10-yl)-4'-methoxy-5-methyl-1,2,3,6-tetrahydro-[1,1'-biphenyl]-3-yl) but-2-enoate (5j)

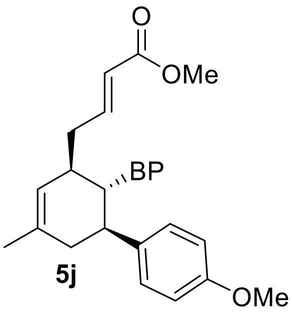

5j

From **1a** (27.5 mg, 0.25 mmol) and BODIPY **2j** (32.4 mg, 0.1 mmol), following the general procedure C (45 ºC, 18h), compound **5j** (39.2 mg, 0.08 mmol) was obtained in 80% yield as a red solid. The crude product was purified by flash column chromatography (gradient Cy/AcOEt from 9:1 to 5:1). The *ee* was determined by SFC using a Chiralpak OJ-H column [CO_2/MeOH (95:5), 120 bar, 40 °C)]; 3.0 mL/min. τ_{may} = 8.714 min, τ_{min} = 6.591 min, *ee*= 95 %. **$[\alpha]^{20}_D$** = +1437 (*c* 0.021, CH_2Cl_2)

^{1}H NMR (300 MHz, $CDCl_3$) δ 7.72 (s, 2H), 7.52 (d, *J* = 4.0 Hz, 1H), 7.18 (d, *J* = 4.2 Hz, 1H), 6.89 – 6.76 (m, 3H), 6.65 – 6.49 (m, 3H), 6.40 (d, *J* = 3.9 Hz, 1H), 5.68 (d, *J* = 15.7 Hz, 1H), 5.46 (s, 1H), 3.72 (s, 3H), 3.64 (s, 3H), 3.60 – 3.39 (m, 1H), 3.21 – 3.07 (m, 2H), 2.50 – 2.21 (m, 3H), 2.15 – 1.97 (m, 1H), 1.80 (s, 3H). **^{13}C NMR (75 MHz, $CDCl_3$)** δ 166.6, 158.2, 152.7, 145.7, 144.6, 141.7, 137.4, 135.7, 133.8, 133.0, 129.6, 128.3 (3C), 123.6, 123.1, 118.0, 117.5, 113.7, 55.1, 51.5, 50.7, 47.9, 43.9, 40.0, 37.1, 23.3. **^{19}F NMR (282 MHz, $CDCl_3$)** δ -144.66 (ddd, *J* = 105.7, 58.7, 29.2 Hz, 1F), -147.83 (ddd, *J* = 105.7, 55.7, 27.8 Hz, 1F). **HRMS (ESI$^+$)** calculated for $C_{28}H_{33}BF_2N_3O_3$ [M + NH_4^+] = 508.2692, found: 508.2563.

Methyl (*E*)-4-((1*R*,5*S*,6*R*)-6-(5,5-difluoro-5*H*-4λ^4,5λ^4-dipyrrolo[1,2-*c*:2',1'-*f*][1,3,2] diazaborinin-10-yl)-5-hexyl-3-methylcyclohex-2-en-1-yl)but-2-enoate (5k)

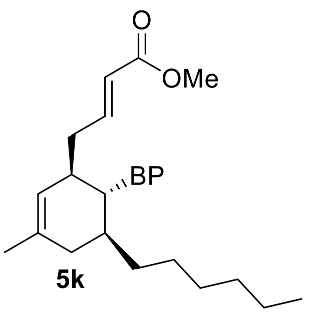

5k

From **1a** (27.5 mg, 0.25 mmol) and BODIPY **2k** (30.2 mg, 0.1 mmol), following the general procedure C (45 ºC, 15h), compound **5k** (35.3 mg, 0.075 mmol) was obtained in 75% yield as a red solid. The crude product was purified by flash column chromatography (gradient Cy/AcOEt from 9:1 to 5:1). The *ee* was determined by SFC using Chiralpak IA column [CO_2/MeOH (95:5), 120 bar, 40 °C)]; 3.0 mL/min. τ_{may} = 8.017 min, τ_{min} = 7.121 min, *ee*= 88 %. **$[\alpha]^{20}_D$** = -182.5 (*c* 0.079, CH_2Cl_2).

^{1}H NMR (300 MHz, $CDCl_3$) δ 7.92 (s, 1H), 7.82 (s, 1H), 7.39 (d, *J* = 4.3 Hz, 1H), 7.27 (d, *J* = 4.3 Hz, 1H), 6.76 (ddd, *J* = 15.1, 9.0, 5.6 Hz, 1H), 6.56 – 6.48 (m, 2H), 5.64 (d, *J* = 15.7 Hz, 1H), 5.36 (s, 1H), 3.70 (s, 3H), 3.04 – 2.88 (m, 1H), 2.66 (t, *J* = 10.9 Hz, 1H), 2.40 – 2.12 (m, 3H), 2.06 – 1.87 (m, 1H), 1.76 (s, 3H), 1.39 – 0.90 (m, 11H), 0.79 (t, *J* = 7.0 Hz, 3H). **^{13}C NMR (75 MHz, $CDCl_3$)** δ 166.6, 154.2, 145.9, 144.6, 142.2, 137.5, 135.1, 132.7, 129.3, 128.3, 123.3, 123.2, 118.1, 117.7, 51.4, 50.5, 43.8, 40.8, 37.0, 36.9, 34.2, 31.6, 28.8, 26.3, 23.5, 22.5, 14.0. **^{19}F NMR (282 MHz, $CDCl_3$)** δ -145.08 (ddd, *J* = 106.1, 57.9, 28.8 Hz, 1F), -146.39 (ddd, *J* = 106.3, 56.8, 28.2 Hz, 1F). **HRMS (ESI$^+$)** calculated for $C_{27}H_{40}N_3O_2BF_2$ $[M+NH_4]^+$: 486.3212, found: 486.3100.

5. Synthesis of compound 8.

A dry vial equipped with a magnetic stir bar was charged with the aminocatalyst **3a** (3.3 mg, 0.01 mmol, 0.1 equiv.), PhCOOH (1.2mg, 0.01 mmol, 0.1 equiv.) and dienal **1a** (29.4mg, 0.25 mmol, 2.5 equiv.). *p*-Xylene (1 mL) was added to dissolve the compounds, then the BODIPY **2a** (27.4mg, 0.1 mmol, 1 equiv.) was added to the mixture. The reaction mixture was stirred at 45 °C for 18h affording the crude with the product **4a**. After that, the reaction crude was added dropwise over a solution of ylide, prepared by reaction of CBr_4 (248.7 mg, 0.75 mmol, 3 equiv.) and PPh_3 (393.5 mg, 1.5 mmol, 6 equiv.) in DCM at -5 °C following the procedure described in the literature.[11] The mixture was stirred at -5 °C during 10 minutes (full conversion was determined by TLC). The crude product was purified by flash column chromatography (gradient of Cy/AcOEt from 9:1 to 4:1) achieving the desired product **8** (32.9mg, 0.05mmol) in 50% yield.

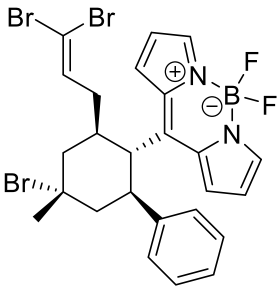

8

^{1}H NMR (300 MHz, $CDCl_3$) δ 8.03 (d, *J* = 4.3 Hz, 1H), 7.79 (s, 1H), 7.69 (s, 1H), 7.14 – 6.97 (m, 4H), 6.96 – 6.86 (m, 2H), 6.64 (dd, *J* = 4.3, 1.9 Hz, 1H), 6.33 (dd, *J* = 4.4, 1.9 Hz, 1H), 6.24 (dd, *J* = 8.5, 6.4 Hz, 1H), 3.88 (td, *J* = 11.6, 3.4 Hz, 1H), 3.10 – 2.94 (m, 1H), 2.88 (t, *J* = 11.4 Hz, 1H), 2.49 – 2.35 (m, 2H), 2.01 (s, 3H), 2.14 – 1.83 (m, 3H), 1.49 (dd, *J* = 14.7, 11.3 Hz, 1H). **^{13}C NMR (75 MHz, $CDCl_3$)** δ 150.9, 144.7, 142.4, 140.8, 136.97, 136.9, 134.9, 132.8, 129.1, 128.8, 128.5 (2C), 127.3 (2C), 127.2, 118.0, 91.4, 68.9, 53.3, 49.5, 48.6, 47.9, 40.8, 37.5, 35.4. **^{19}F NMR (282 MHz, $CDCl_3$)** δ -144.90 (ddd, *J* = 105.1, 58.0, 29.0 Hz), -147.53 (ddd, *J* = 105.2, 56.2, 28.1 Hz). **HRMS (ESI^{+})** calculated for $C_{25}H_{25}N_2BBr_3F_2$ $[M+H]^{+}$: 638,9623, found: 638,9640.

6. X-Ray structure of compound 8

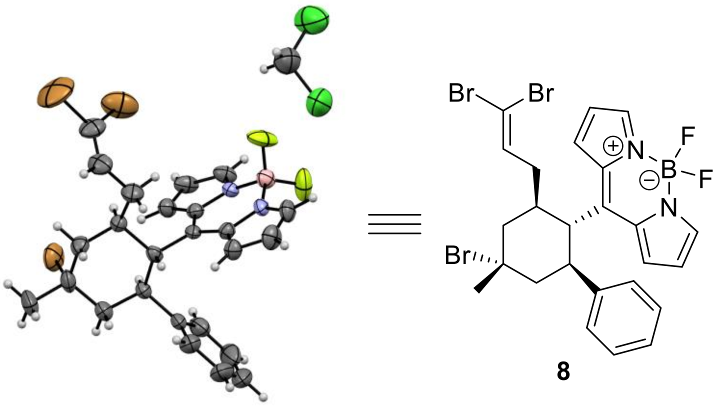

8

7. UV-VIS absorption and fluorescence emission spectra of products 5

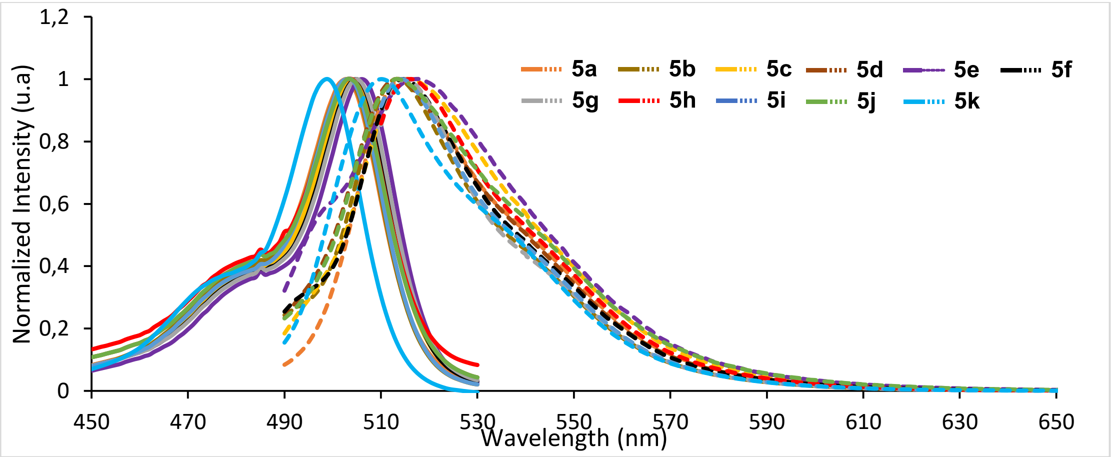

Figure S1: UV-vis absorption and fluorescence emission spectra of final products **5** dissolved in acetonitrile.

8. NMR SPECTRA

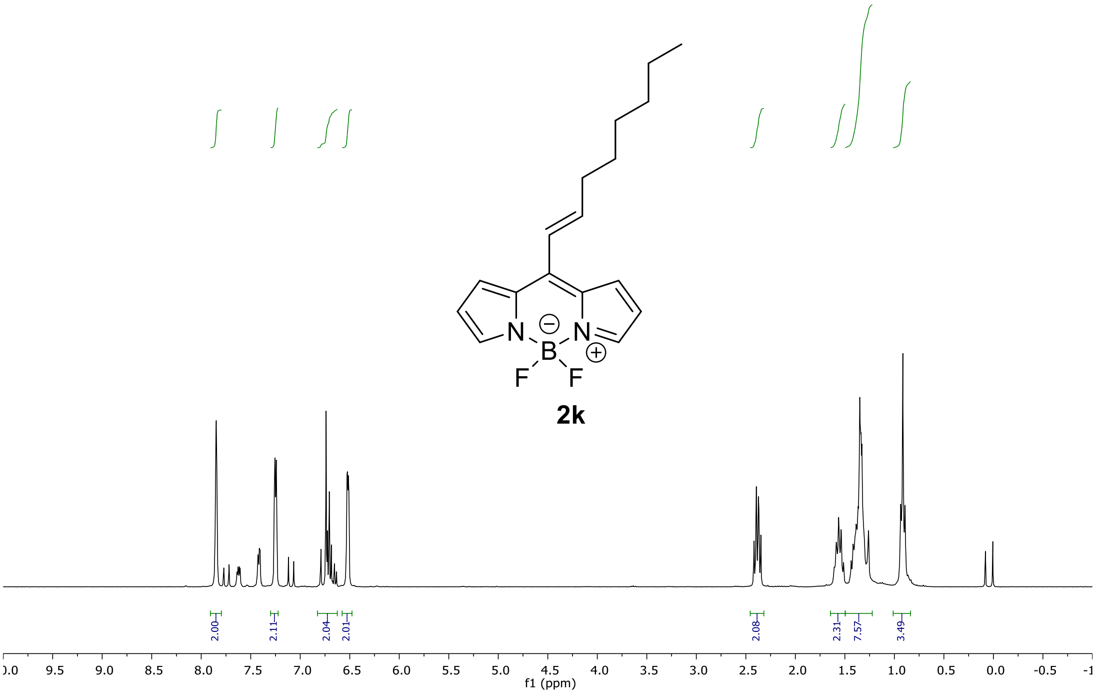

2k

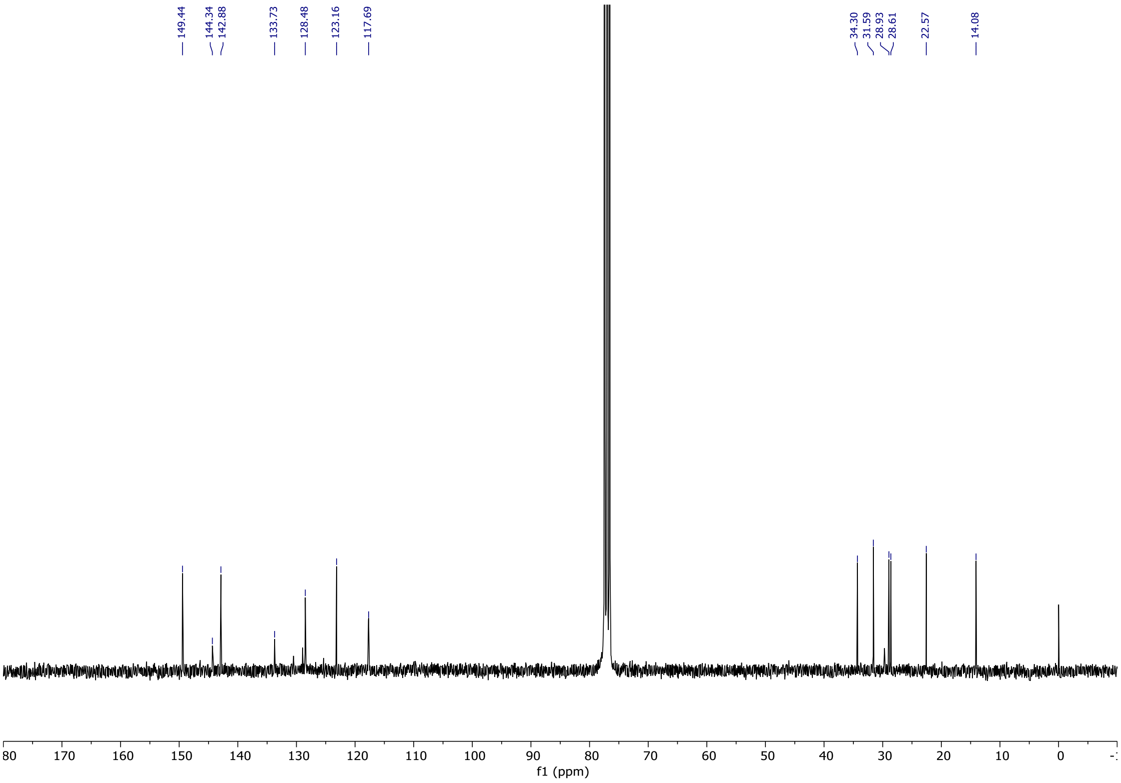

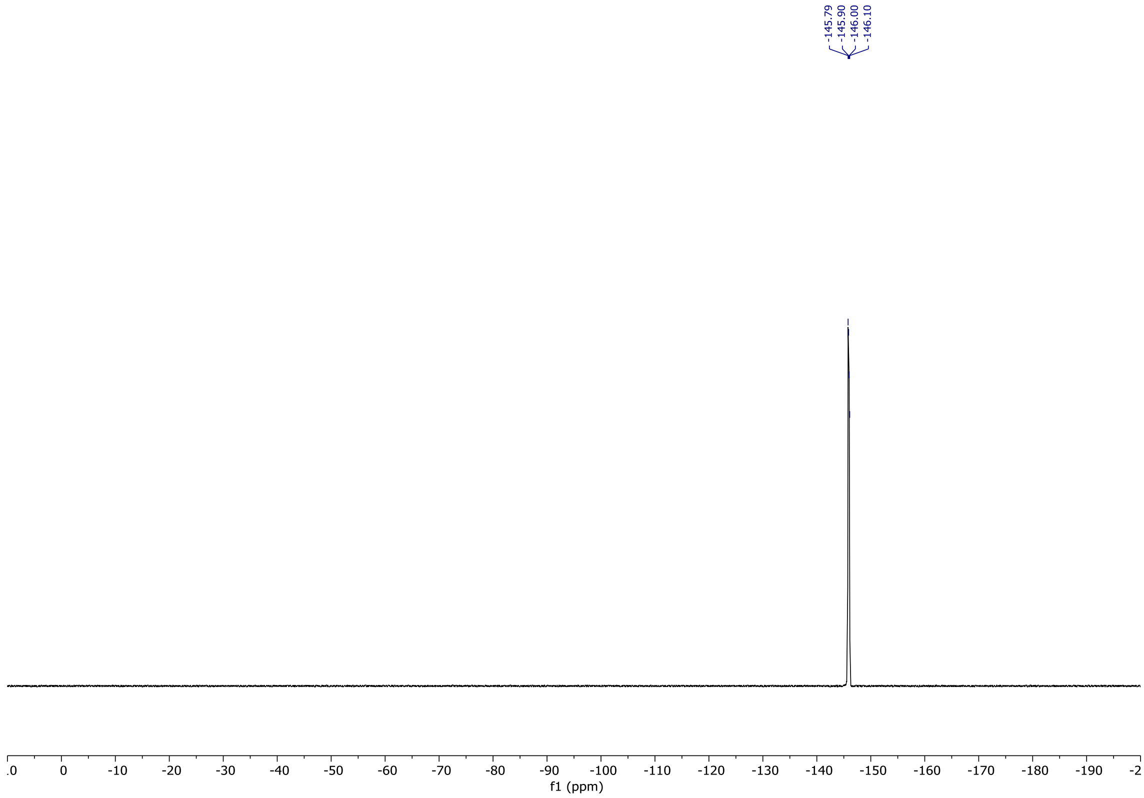
-145.79
-145.90
-146.00
-146.10
f1 (ppm)

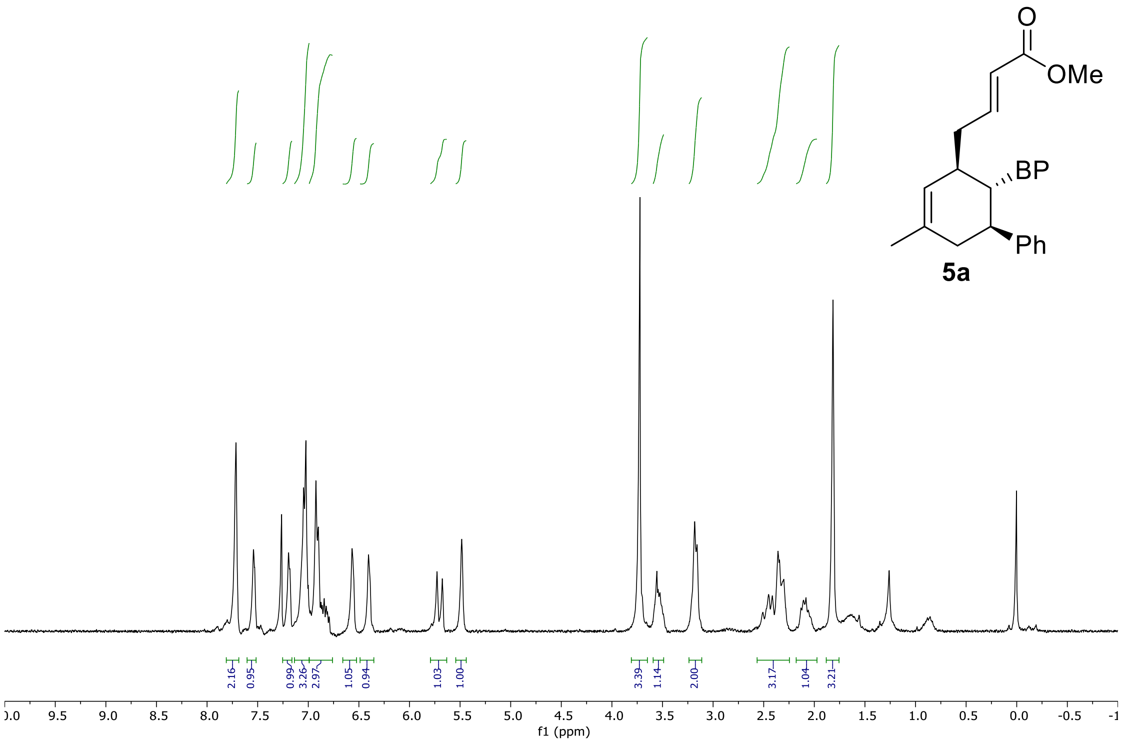
O
OMe
BP
Ph
5a
2.16
0.95
0.99
3.26
2.97
1.05
0.94
1.03
1.00
3.39
1.14
2.00
3.17
1.04
3.21
f1 (ppm)

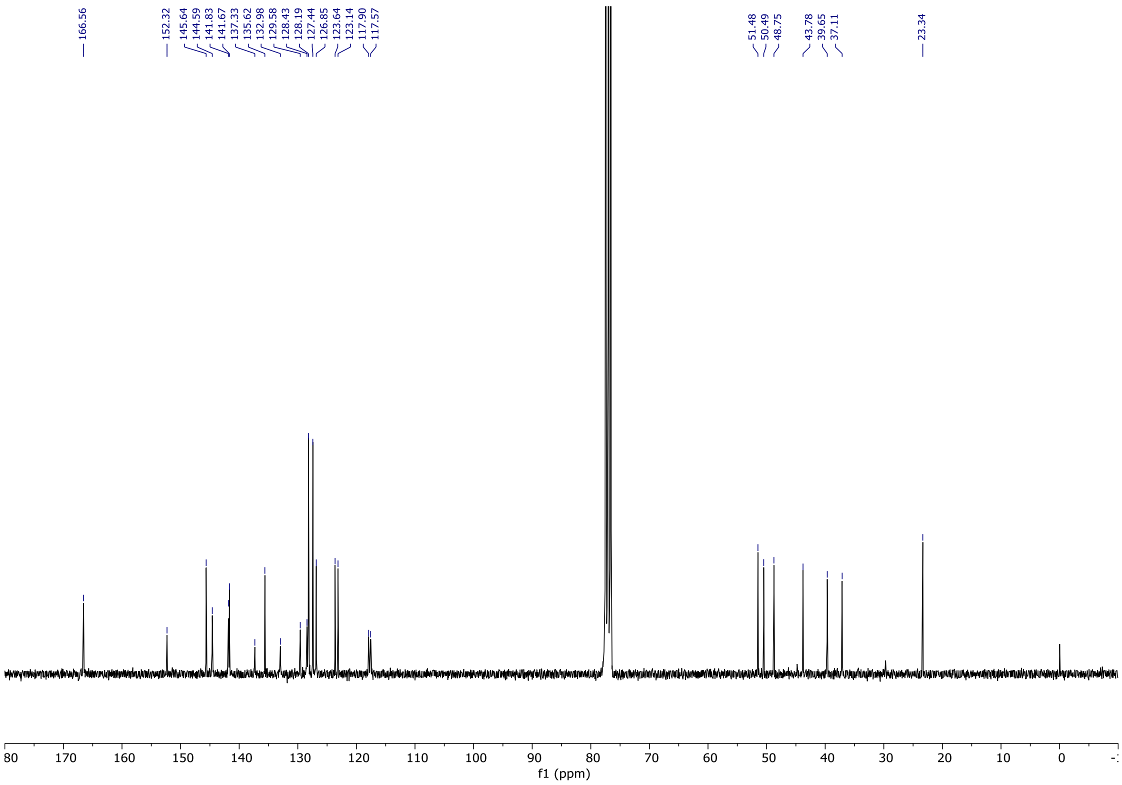
166.56
152.32
145.64
144.59
141.83
141.67
137.33
135.62
132.98
129.58
128.43
128.19
127.44
126.85
123.64
123.14
117.90
117.57
51.48
50.49
48.75
43.78
39.65
37.11
23.34
f1 (ppm)

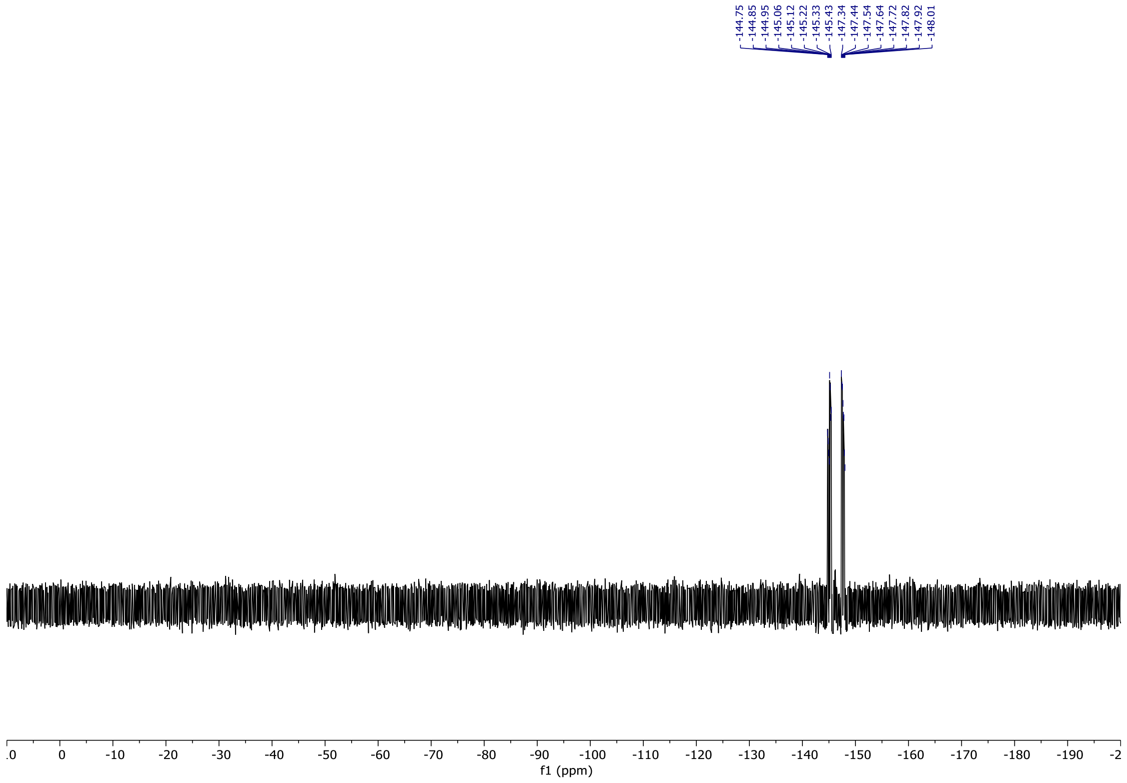
-144.75
-144.85
-144.95
-145.06
-145.12
-145.22
-145.33
-145.43
-147.34
-147.44
-147.54
-147.64
-147.72
-147.82
-147.92
-148.01
f1 (ppm)

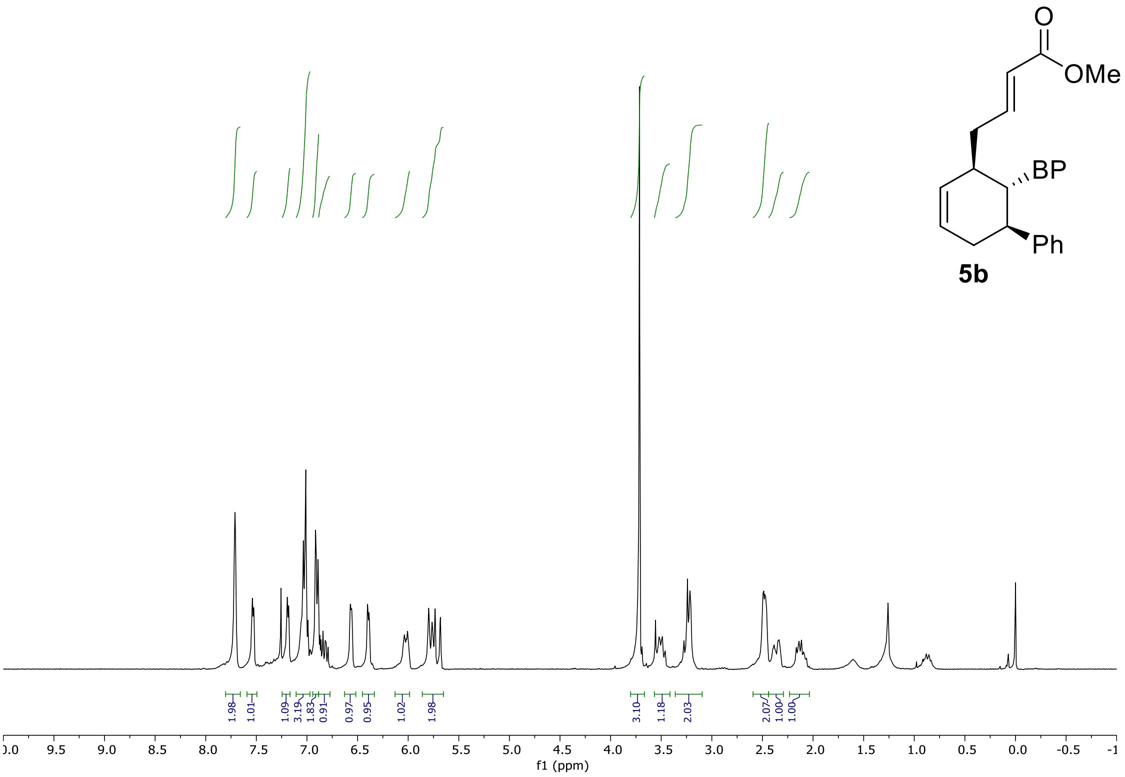
O
OMe
BP
Ph
1.98
1.01
1.09
3.19
1.83
0.91
0.97
0.95
1.02
1.98
3.10
1.18
2.03
2.07
1.00
1.00
f1 (ppm)

5b

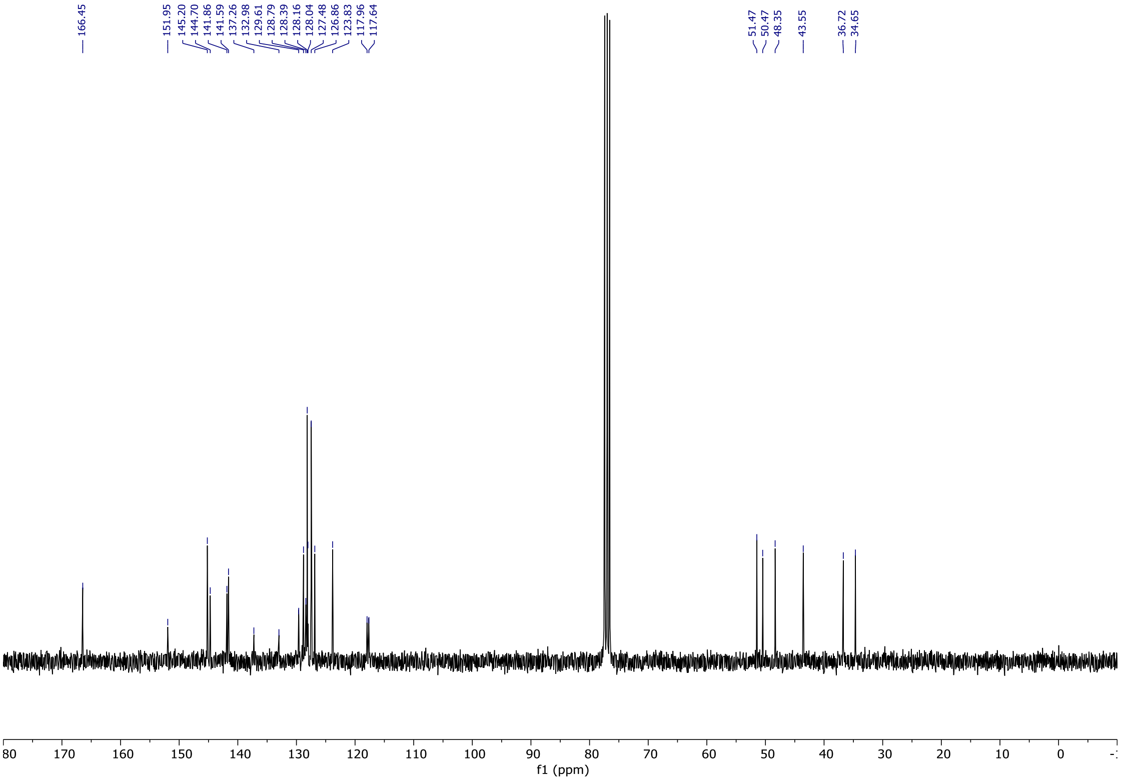
166.45
151.95
145.20
144.70
141.86
141.59
137.26
132.98
129.61
128.79
128.39
128.16
128.04
127.48
126.86
123.83
117.96
117.64
51.47
50.47
48.35
43.55
36.72
34.65
f1 (ppm)

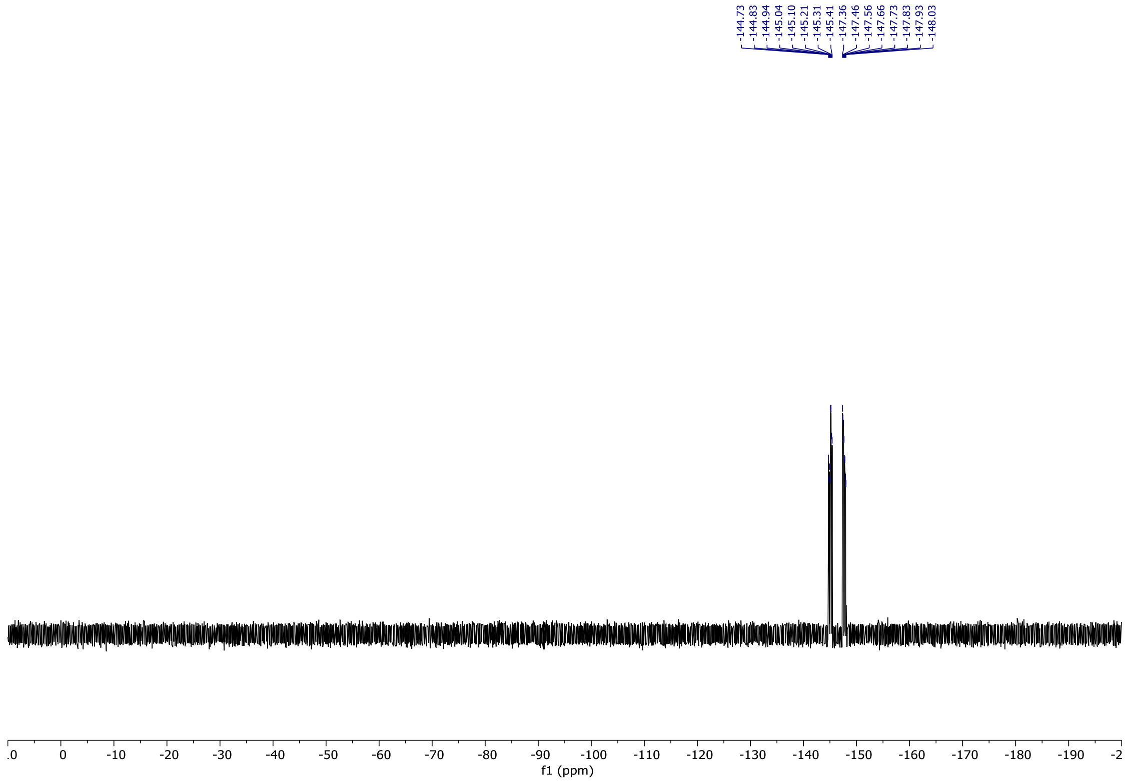
-144.73
-144.83
-144.94
-145.04
-145.10
-145.21
-145.31
-145.41
-147.36
-147.46
-147.56
-147.66
-147.73
-147.83
-147.93
-148.03
.0
0
-10
-20
-30
-40
-50
-60
-70
-80
-90
-100
-110
-120
-130
-140
-150
-160
-170
-180
-190
-2
f1 (ppm)

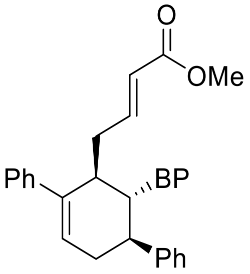
O
OMe
Ph
BP
Ph

5c

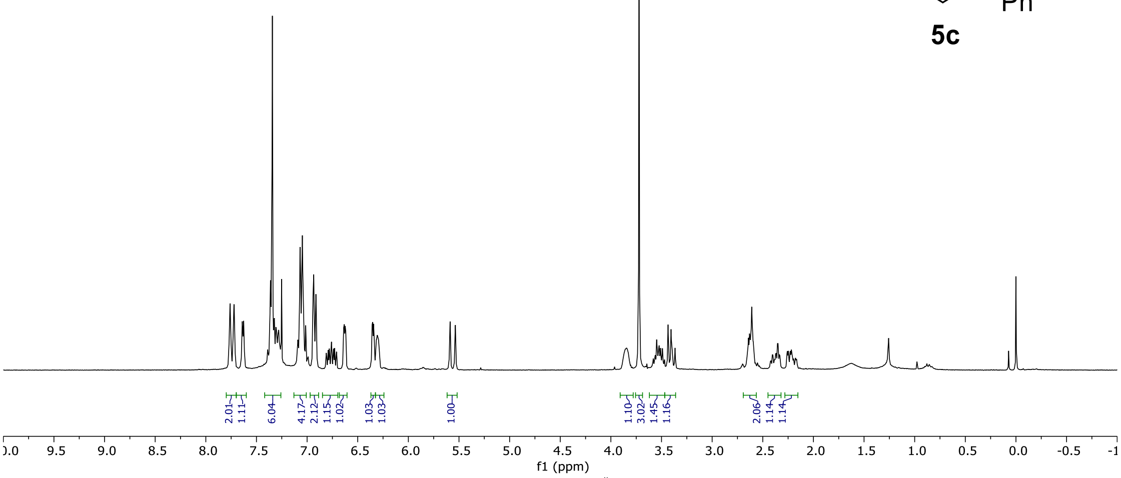
2.01
1.11
6.04
4.17
2.12
1.15
1.02
1.03
1.03
1.00
1.10
3.02
1.45
1.16
2.06
1.14
1.14
f1 (ppm)

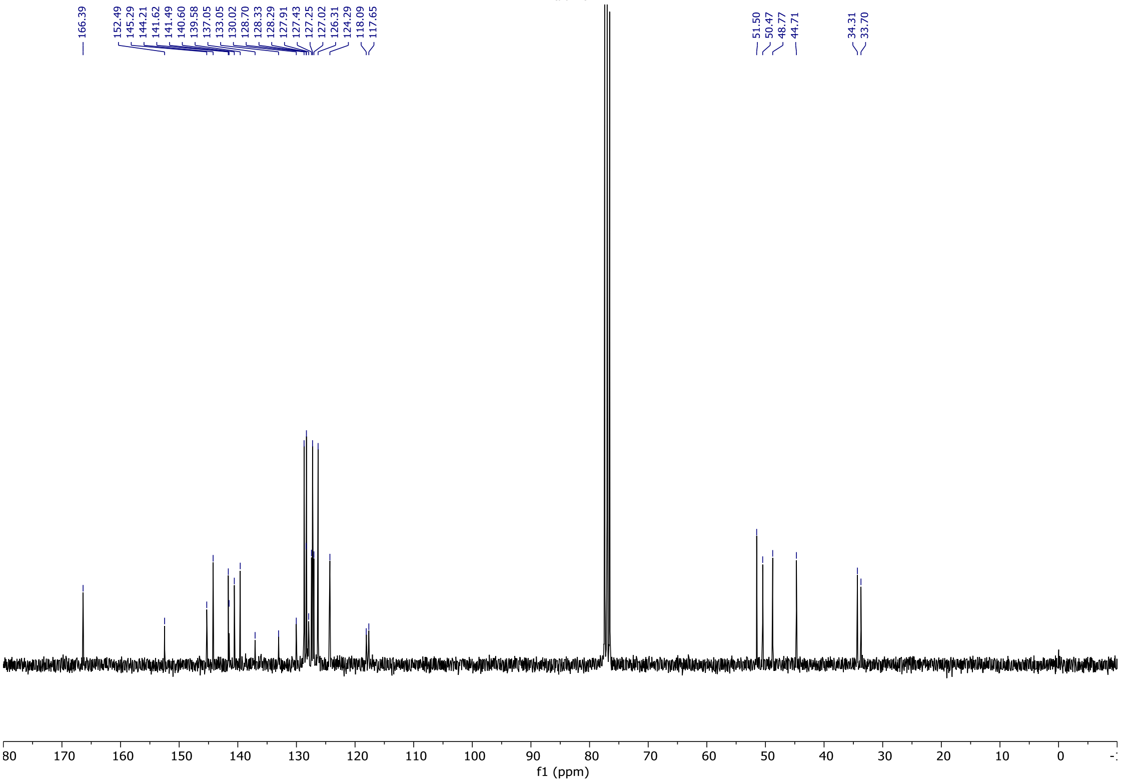
166.39
152.49
145.29
144.21
141.62
141.49
140.60
139.58
137.05
133.05
130.02
128.70
128.33
128.29
127.91
127.43
127.25
127.02
126.31
124.29
118.09
117.65
51.50
50.47
48.77
44.71
34.31
33.70
f1 (ppm)

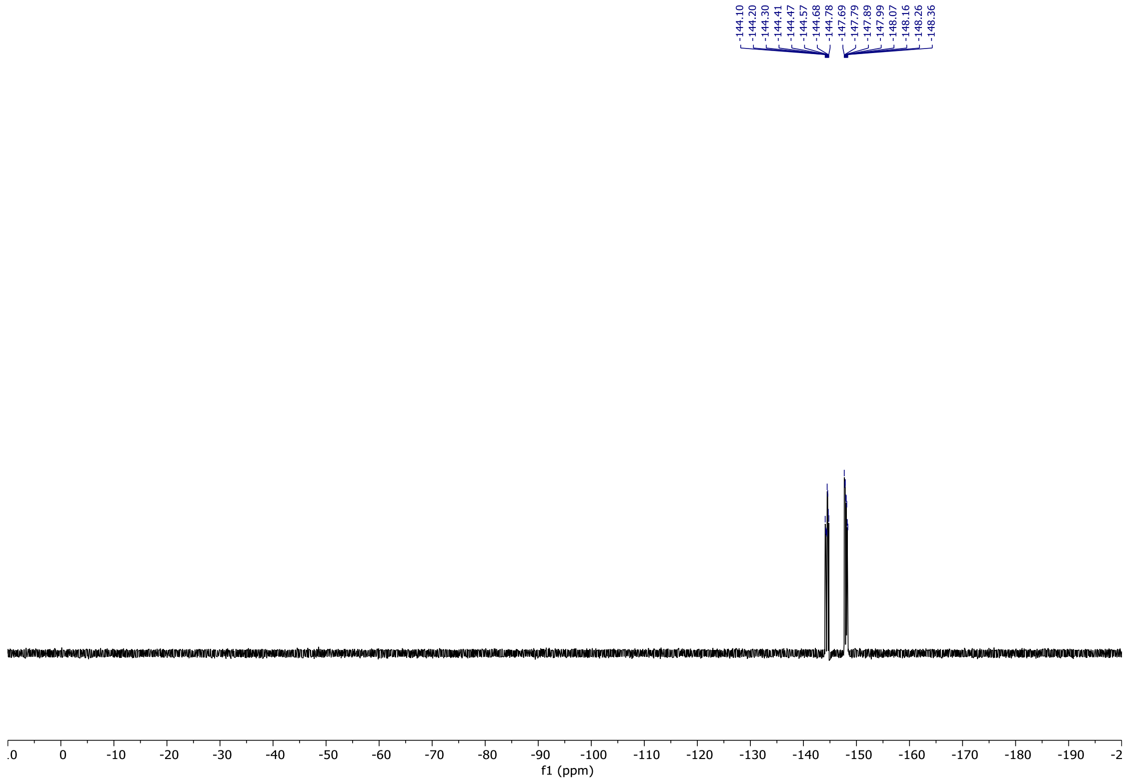
-144.10
-144.20
-144.30
-144.41
-144.47
-144.57
-144.68
-144.78
-147.69
-147.79
-147.89
-147.99
-148.07
-148.16
-148.26
-148.36
0
-10
-20
-30
-40
-50
-60
-70
-80
-90
-100
-110
-120
-130
-140
-150
-160
-170
-180
-190
f1 (ppm)

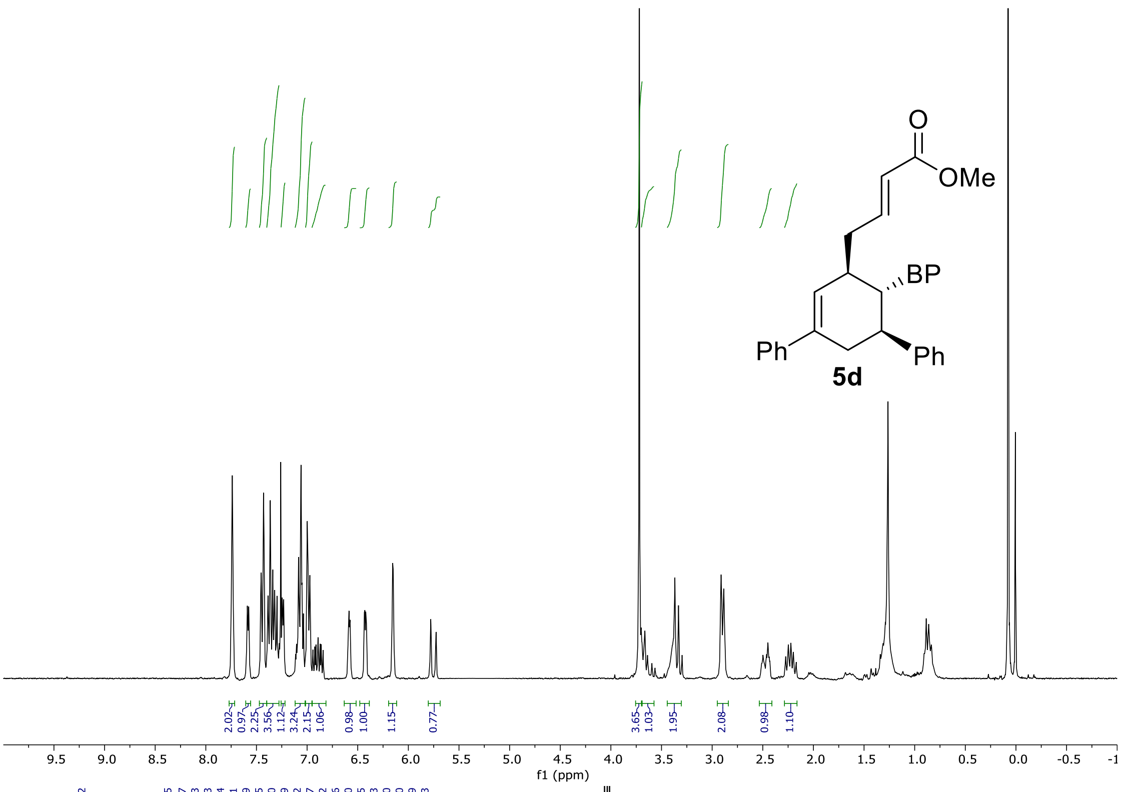

O
OMe
BP
Ph
Ph
5d
2.02
0.97
2.25
3.56
1.12
3.24
2.15
1.06
0.98
1.00
1.15
0.77
3.65
1.03
1.95
2.08
0.98
1.10
f1 (ppm)

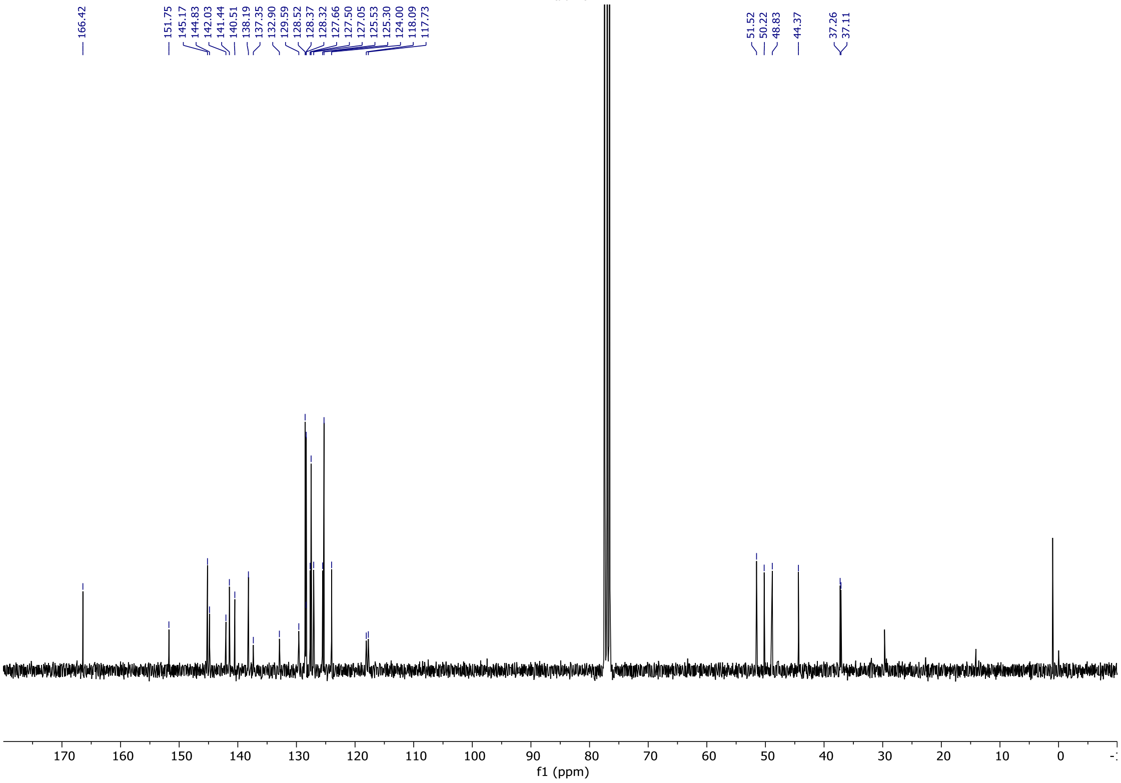

166.42
151.75
145.17
144.83
142.03
141.44
140.51
138.19
137.35
132.90
129.59
128.52
128.37
128.32
127.66
127.50
127.05
125.53
125.30
124.00
118.09
117.73
51.52
50.22
48.83
44.37
37.26
37.11
f1 (ppm)

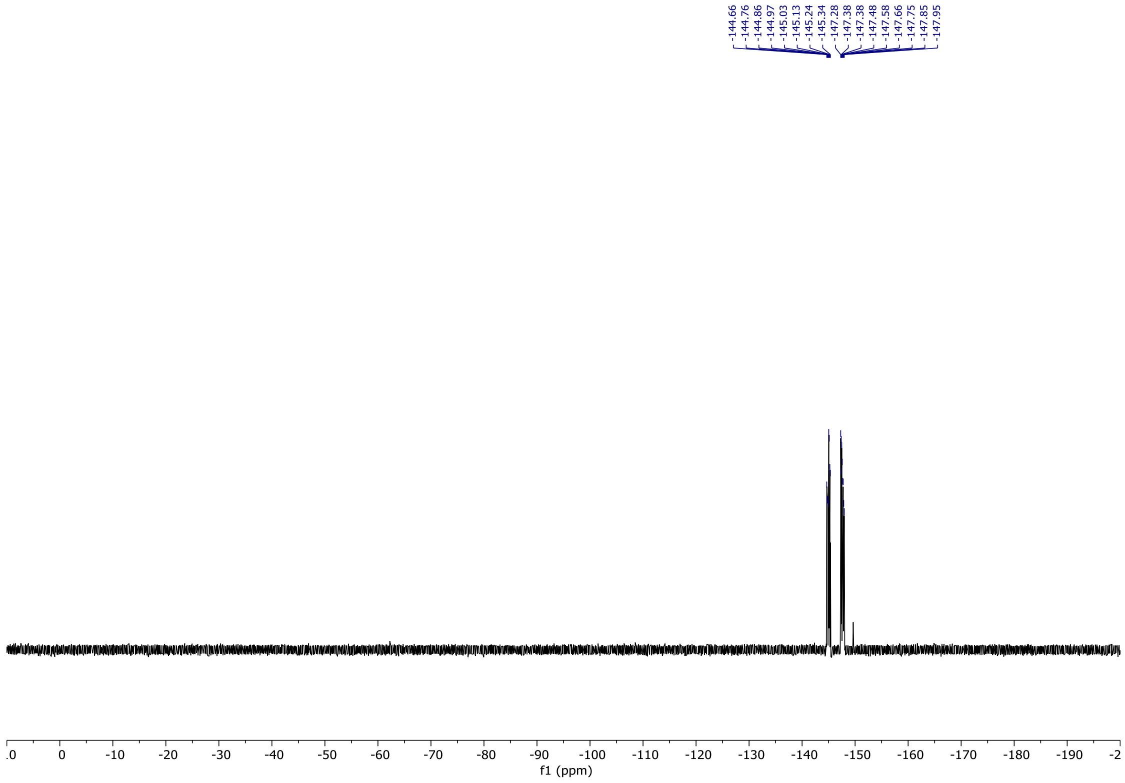
-144.66
-144.76
-144.86
-144.97
-145.03
-145.13
-145.24
-145.34
-147.28
-147.38
-147.38
-147.48
-147.58
-147.66
-147.75
-147.85
-147.95
f1 (ppm)

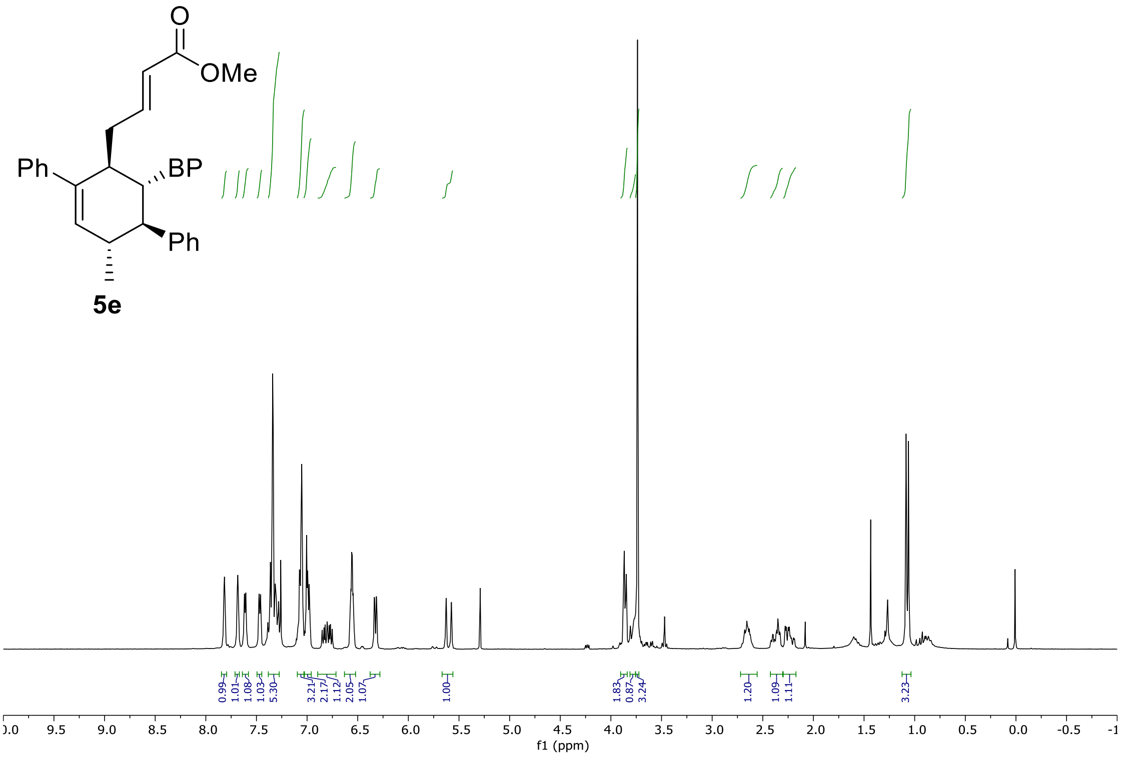
O
OMe
Ph
BP
Ph

5e

0.99
1.01
1.08
1.03
5.30
3.21
2.17
1.12
2.05
1.07
1.00
1.83
0.87
3.24
1.20
1.09
1.11
3.23
f1 (ppm)

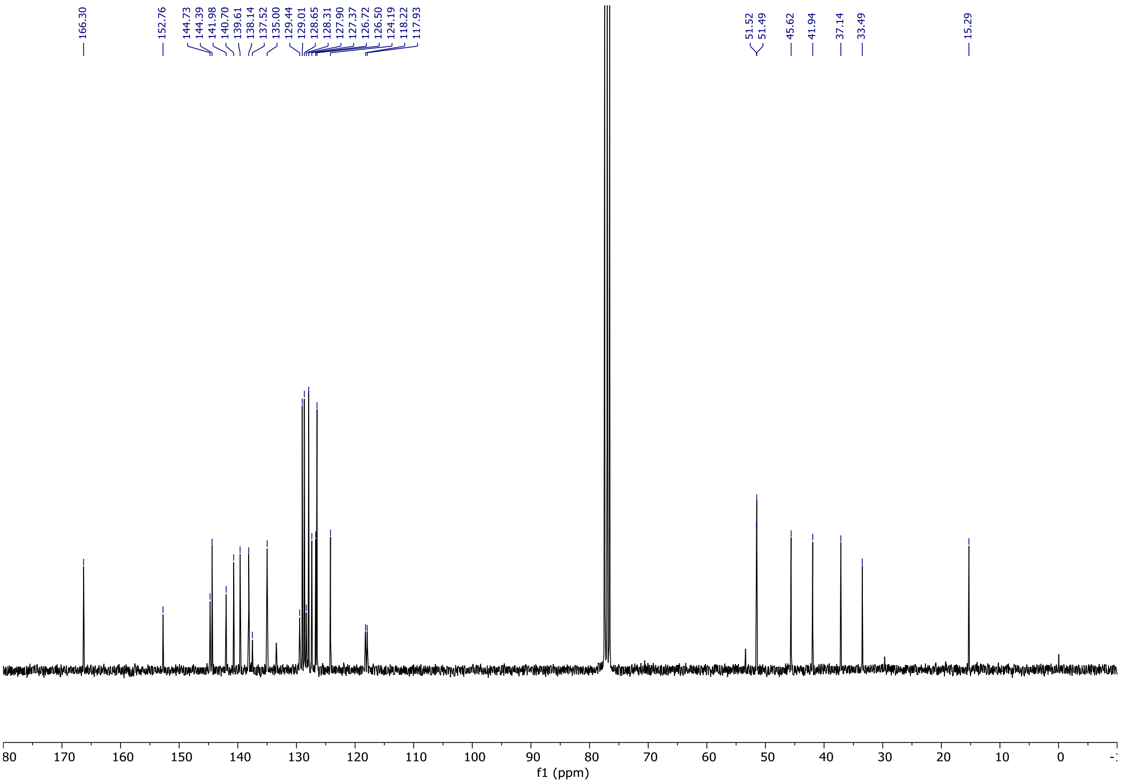
166.30
152.76
144.73
144.39
141.98
140.70
139.61
138.14
137.52
135.00
129.44
129.01
128.65
128.31
127.90
127.37
126.72
126.50
124.19
118.22
117.93
51.52
51.49
45.62
41.94
37.14
33.49
15.29
f1 (ppm)

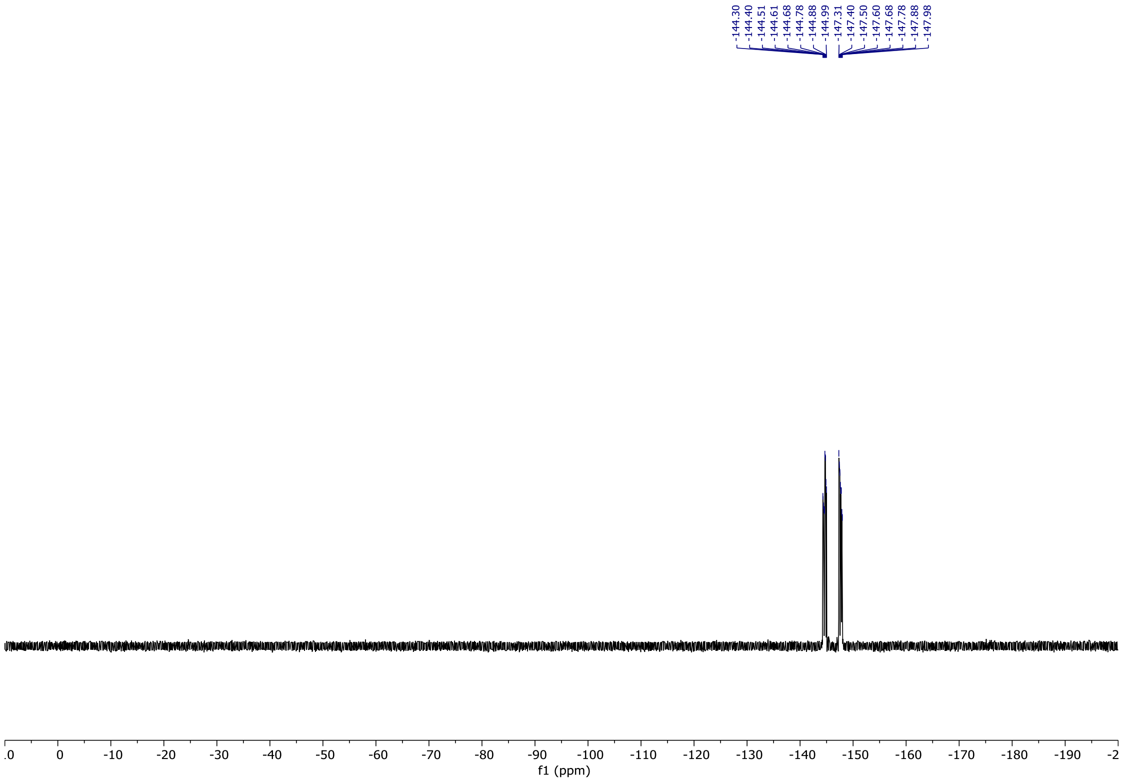

-144.30
-144.40
-144.51
-144.61
-144.68
-144.78
-144.88
-144.99
-147.31
-147.40
-147.50
-147.60
-147.68
-147.78
-147.88
-147.98
.0 0 -10 -20 -30 -40 -50 -60 -70 -80 -90 -100 -110 -120 -130 -140 -150 -160 -170 -180 -190 -2
f1 (ppm)

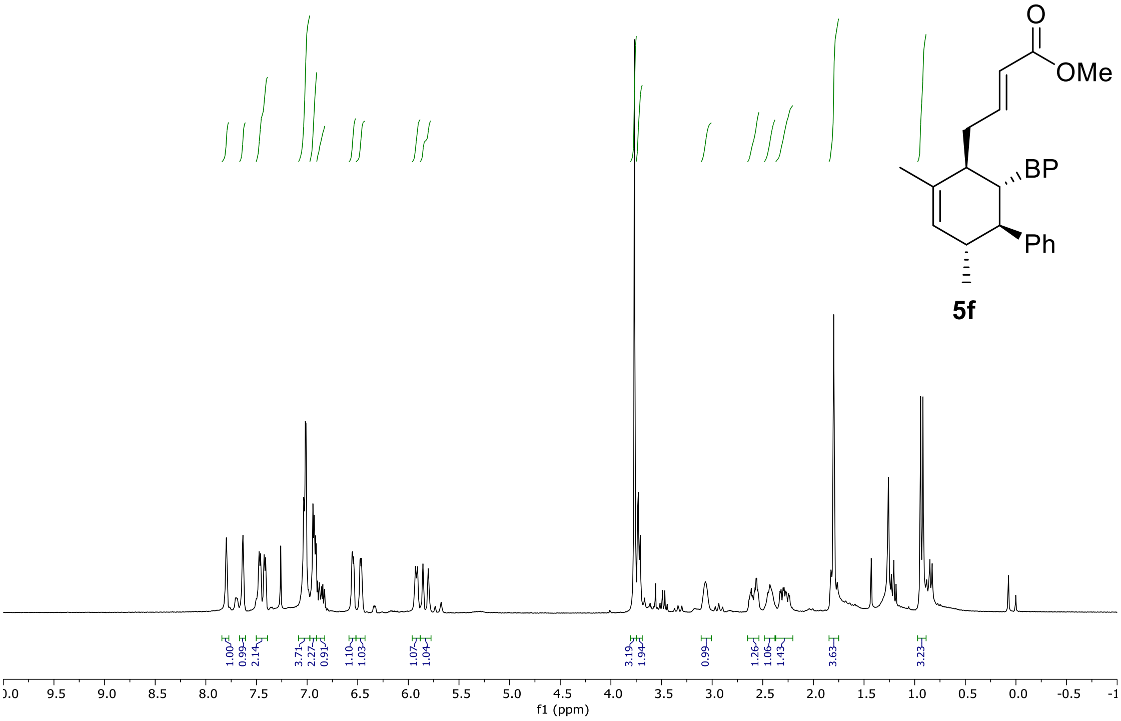
O
OMe
BP
Ph
5f
1.00
0.99
2.14
3.71
2.27
0.91
1.10
1.03
1.07
1.04
3.19
1.94
0.99
1.26
1.06
1.43
3.63
3.23
f1 (ppm)

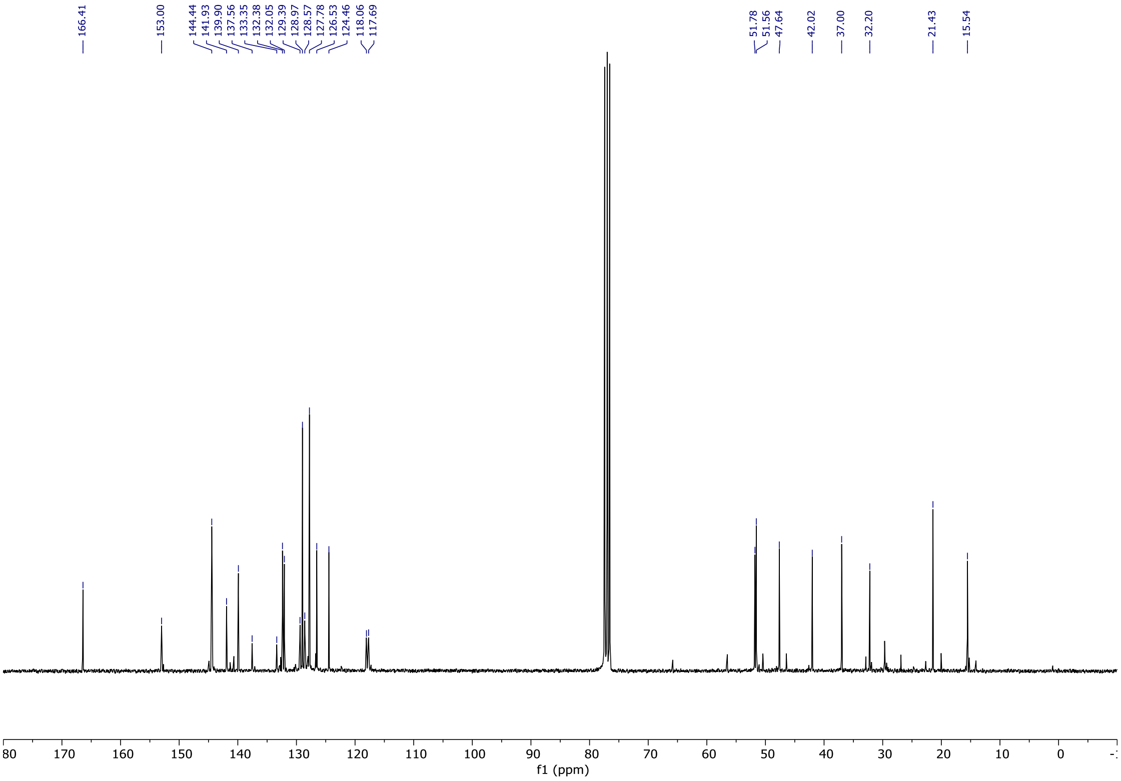
166.41
153.00
144.44
141.93
139.90
137.56
133.35
132.38
132.05
129.39
128.97
128.57
127.78
126.53
124.46
118.06
117.69
51.78
51.56
47.64
42.02
37.00
32.20
21.43
15.54
f1 (ppm)

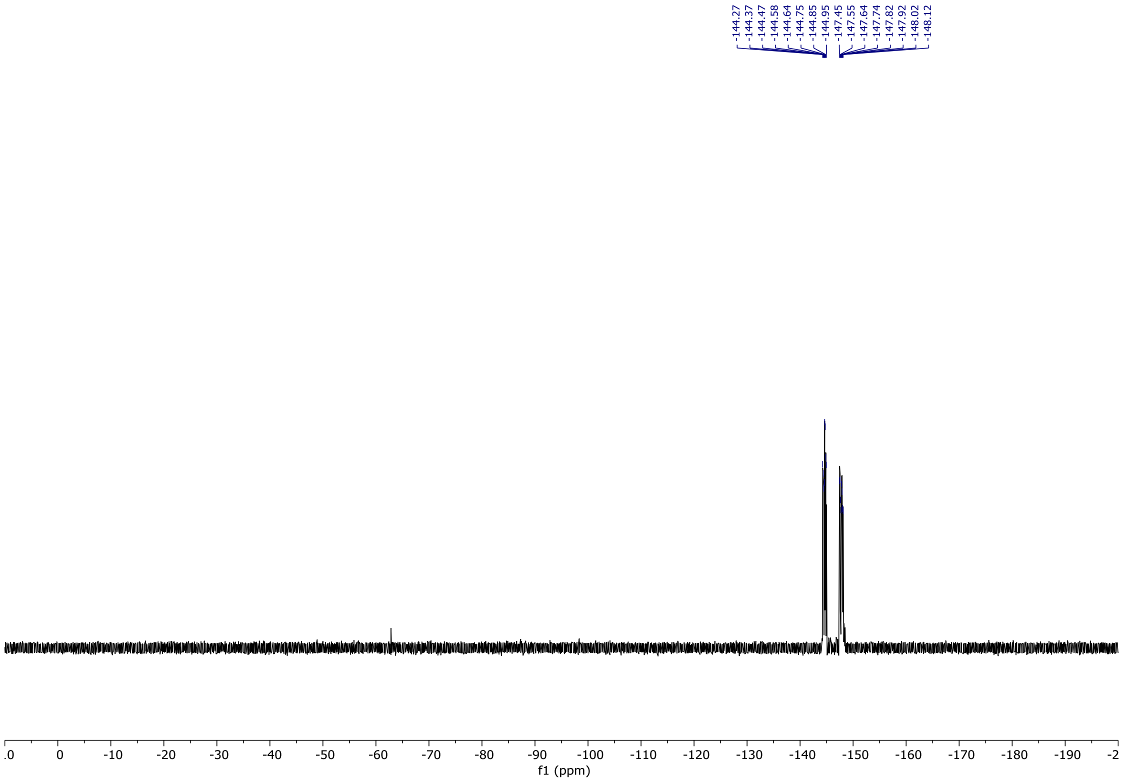
-144.27
-144.37
-144.47
-144.58
-144.64
-144.75
-144.85
-144.95
-147.45
-147.55
-147.64
-147.74
-147.82
-147.92
-148.02
-148.12
f1 (ppm)

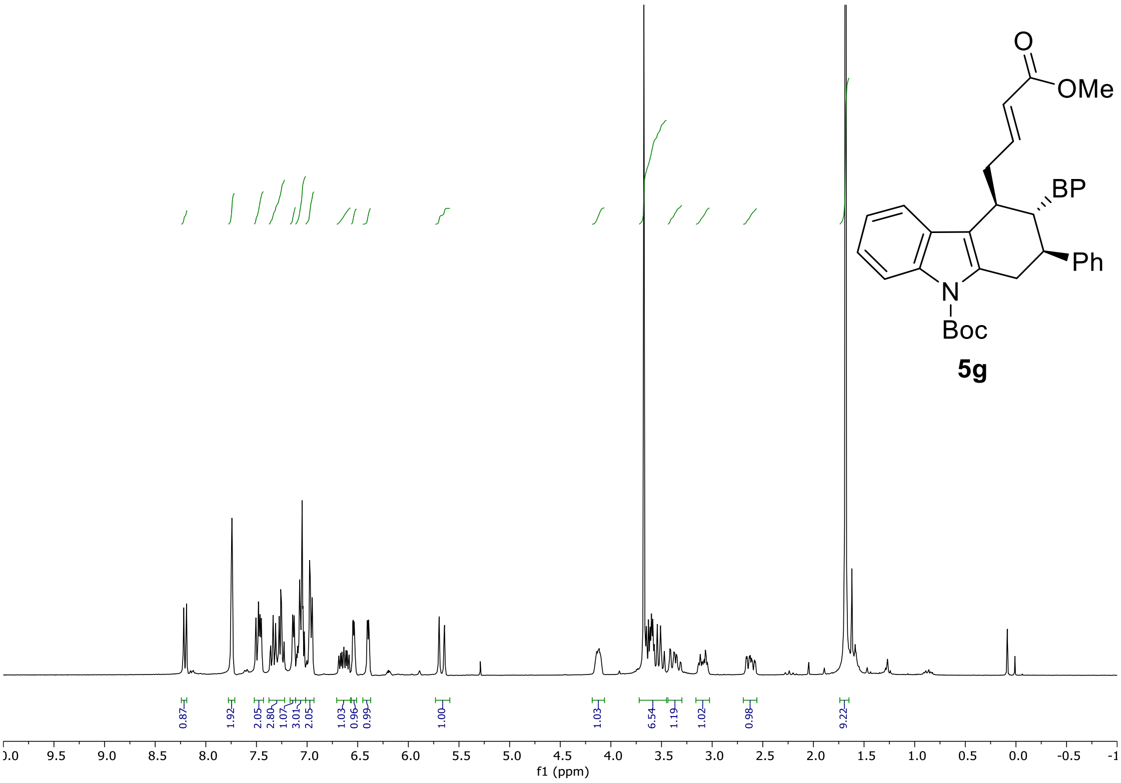
O
OMe
BP
Ph
N
Boc
5g
0.87
1.92
2.05
2.80
1.07
3.01
2.05
1.03
0.96
0.99
1.00
1.03
6.54
1.19
1.02
0.98
9.22
f1 (ppm)

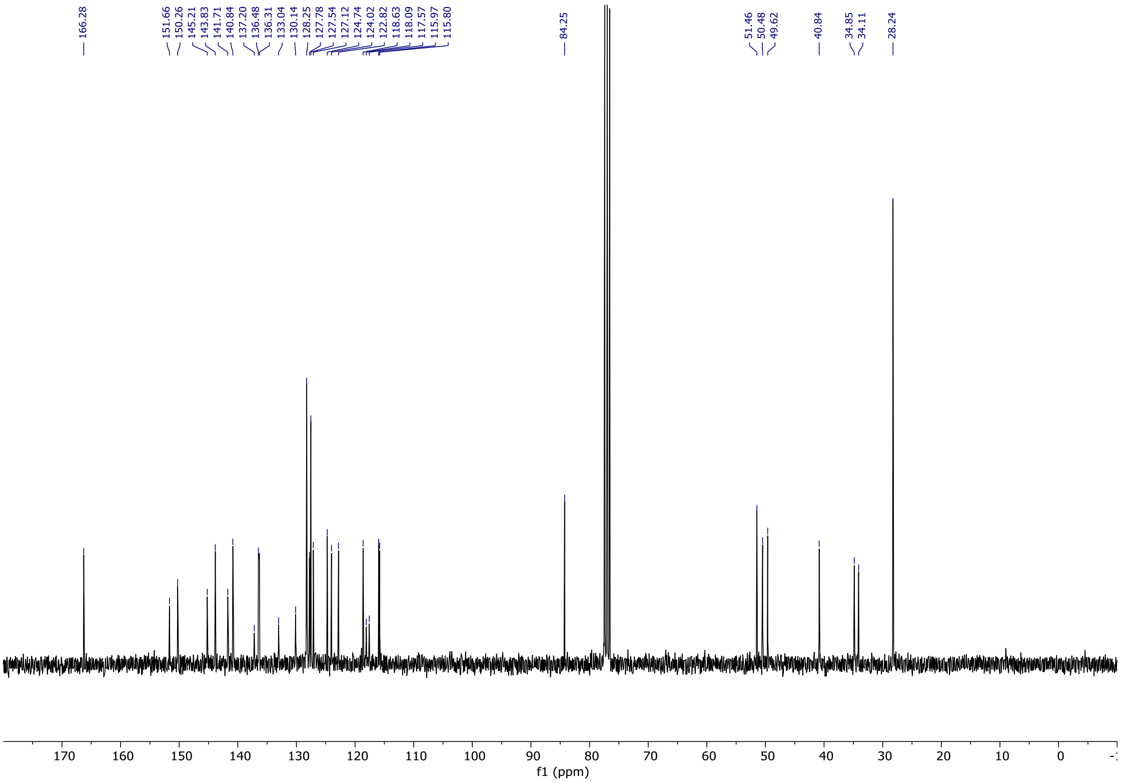
166.28
151.66
150.26
145.21
143.83
141.71
140.84
137.20
136.48
136.31
133.04
130.14
128.25
127.78
127.54
127.12
124.74
124.02
122.82
118.63
118.09
117.57
115.97
115.80
84.25
51.46
50.48
49.62
40.84
34.85
34.11
28.24
f1 (ppm)

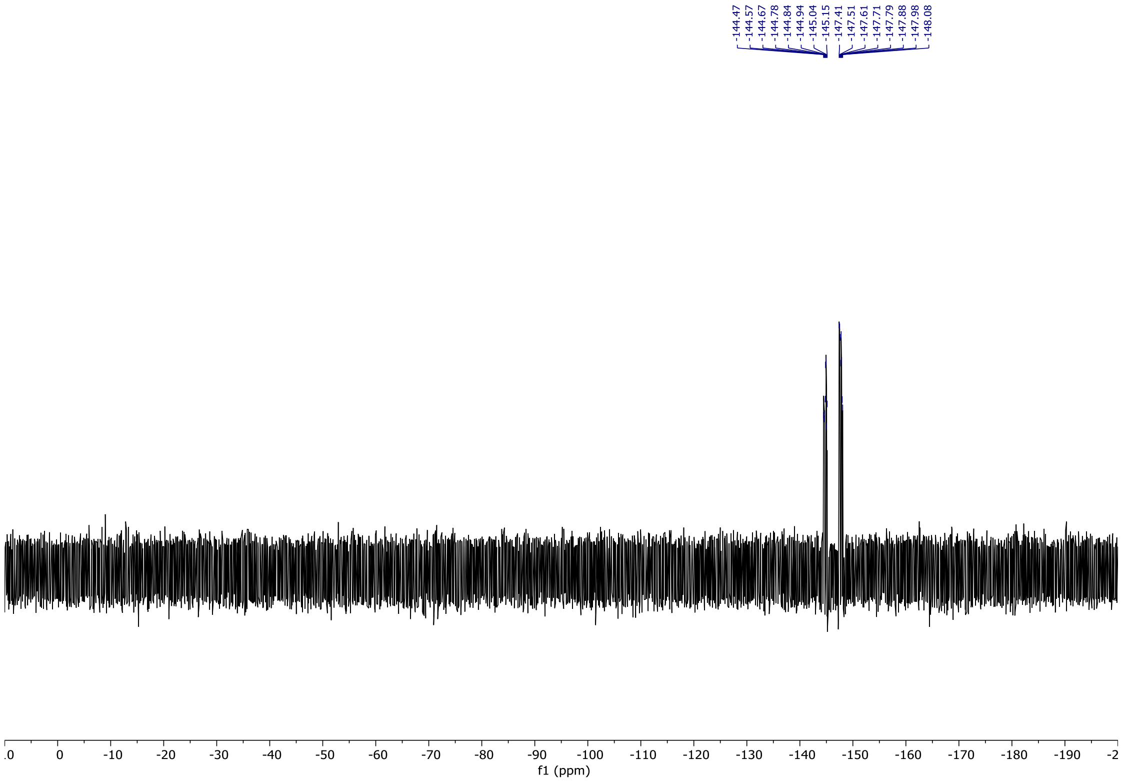

-144.47
-144.57
-144.67
-144.78
-144.84
-144.94
-145.04
-145.15
-147.41
-147.51
-147.61
-147.71
-147.79
-147.88
-147.98
-148.08
f1 (ppm)

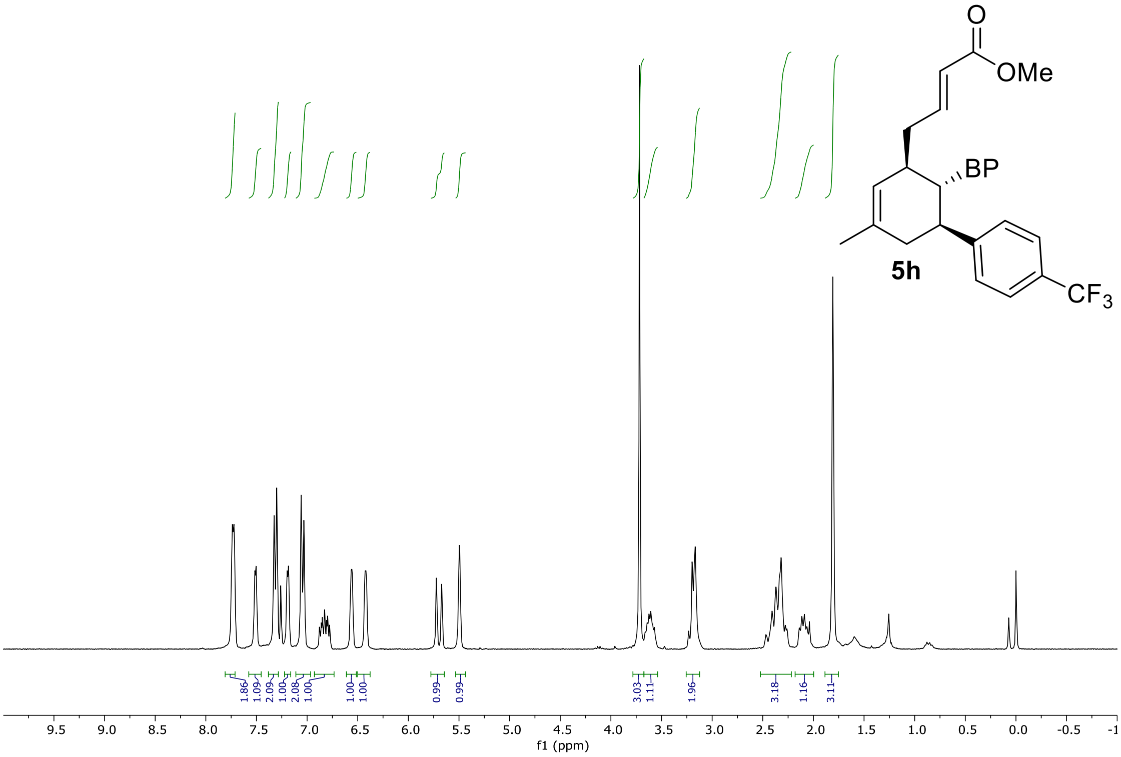

OMe
O
BP
CF3
5h
1.86
1.09
2.09
1.00
2.08
1.00
1.00
1.00
0.99
0.99
3.03
1.11
1.96
3.18
1.16
3.11
9.5 9.0 8.5 8.0 7.5 7.0 6.5 6.0 5.5 5.0 4.5 4.0 3.5 3.0 2.5 2.0 1.5 1.0 0.5 0.0 -0.5
f1 (ppm)

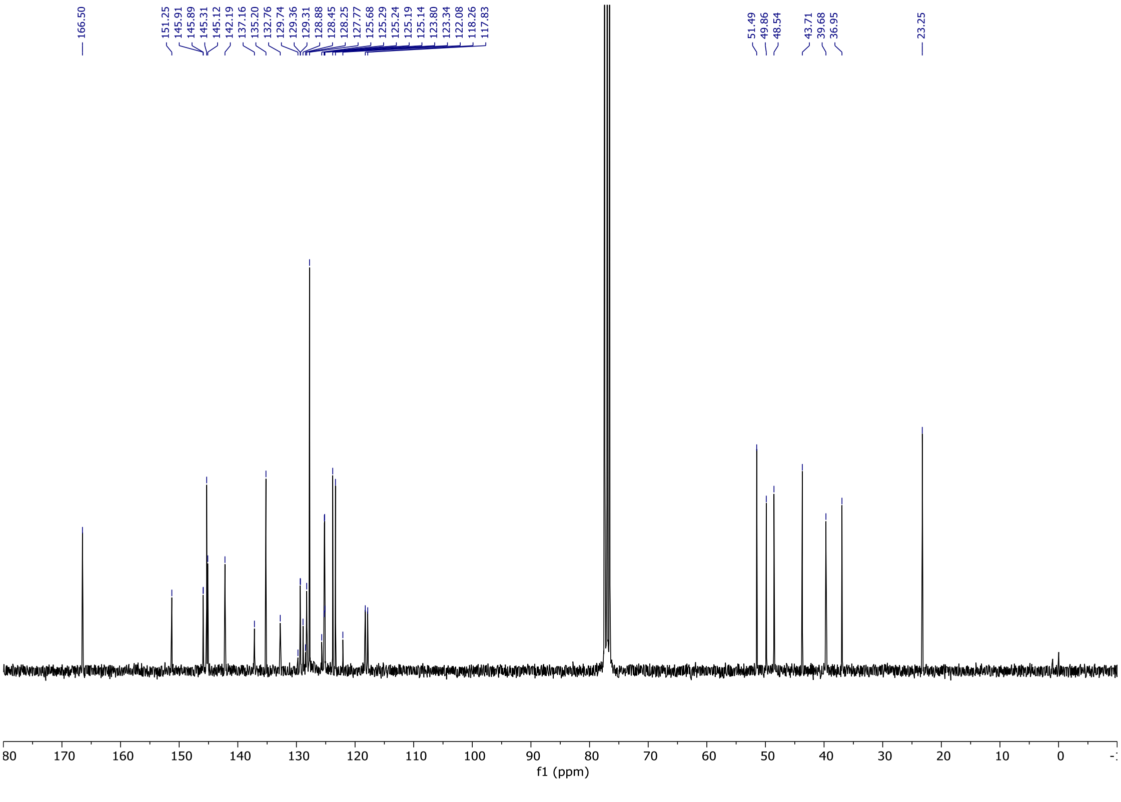

166.50
151.25
145.91
145.89
145.31
145.12
142.19
137.16
135.20
132.76
129.74
129.36
129.31
128.88
128.45
128.25
127.77
125.68
125.29
125.24
125.19
125.14
123.80
123.34
122.08
118.26
117.83
51.49
49.86
48.54
43.71
39.68
36.95
23.25
170 160 150 140 130 120 110 100 90 80 70 60 50 40 30 20 10 0
f1 (ppm)

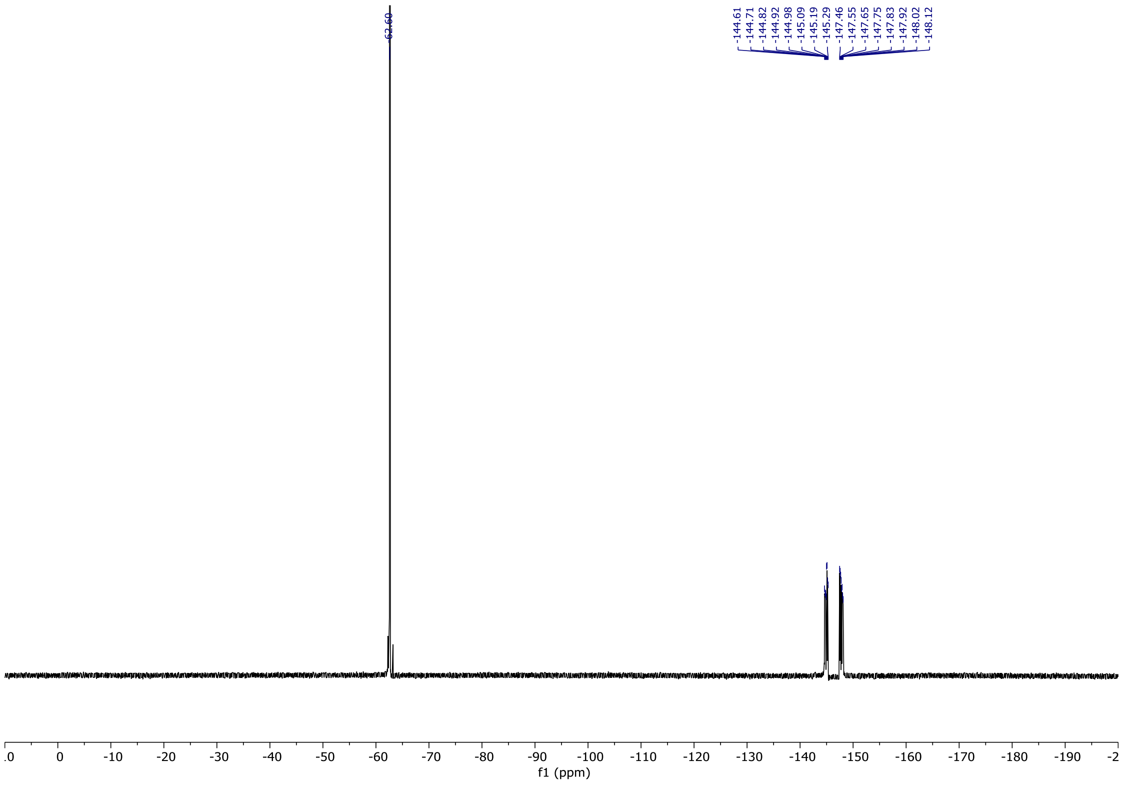
-62.60
-144.61
-144.71
-144.82
-144.92
-144.98
-145.09
-145.19
-145.29
-147.46
-147.55
-147.65
-147.75
-147.83
-147.92
-148.02
-148.12
.0
0
-10
-20
-30
-40
-50
-60
-70
-80
-90
-100
-110
-120
-130
-140
-150
-160
-170
-180
-190
-2
f1 (ppm)

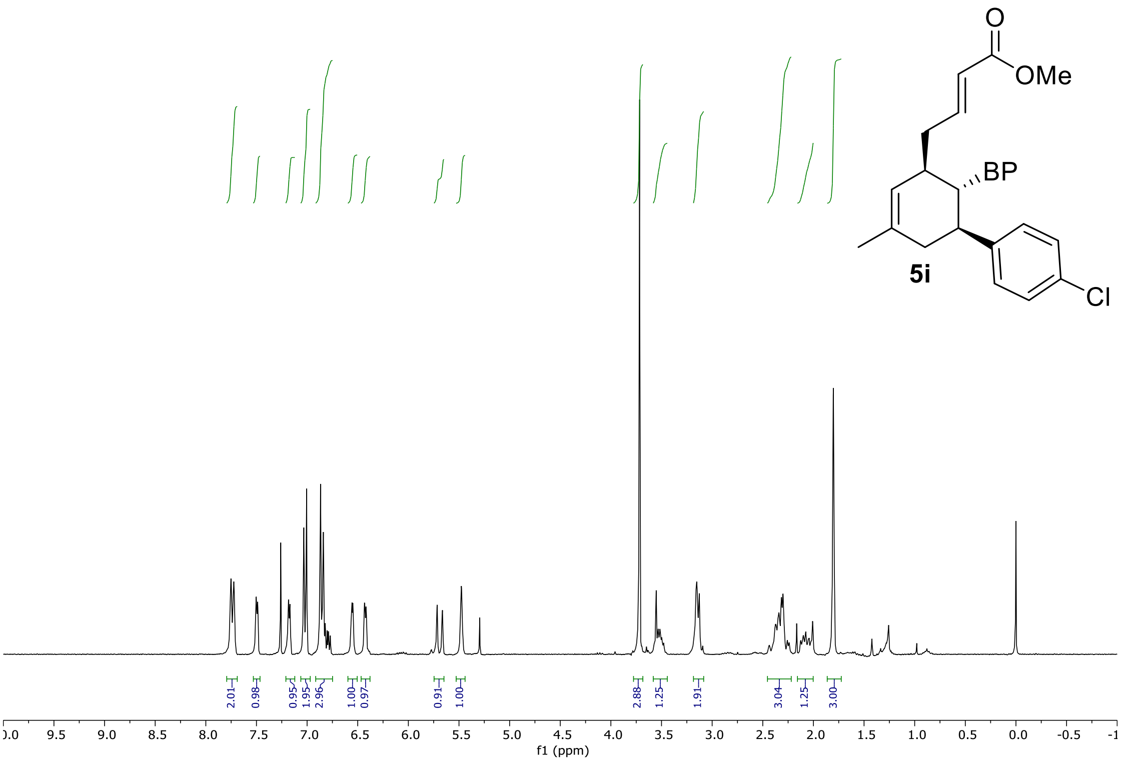
O
OMe
BP
5i
Cl
2.01
0.98
0.95
1.95
2.96
1.00
0.97
0.91
1.00
2.88
1.25
1.91
3.04
1.25
3.00
f1 (ppm)

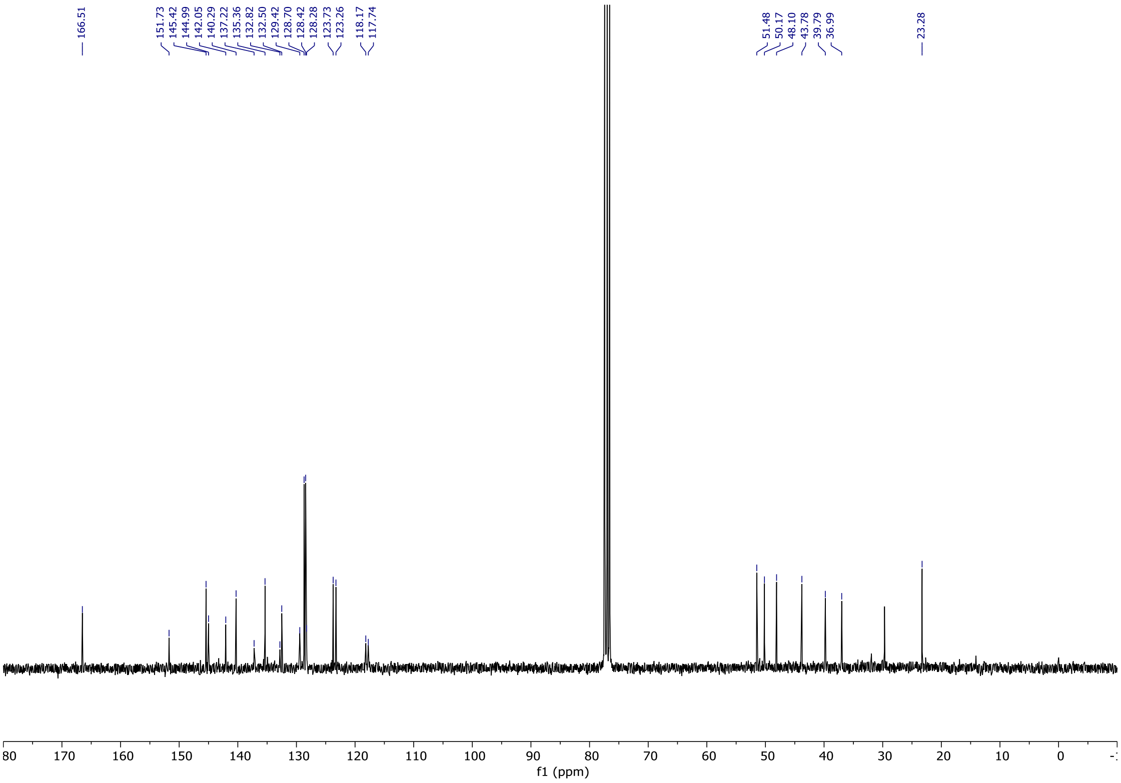
166.51
151.73
145.42
144.99
142.05
140.29
137.22
135.36
132.82
132.50
129.42
128.70
128.42
128.28
123.73
123.26
118.17
117.74
51.48
50.17
48.10
43.78
39.79
36.99
23.28
f1 (ppm)

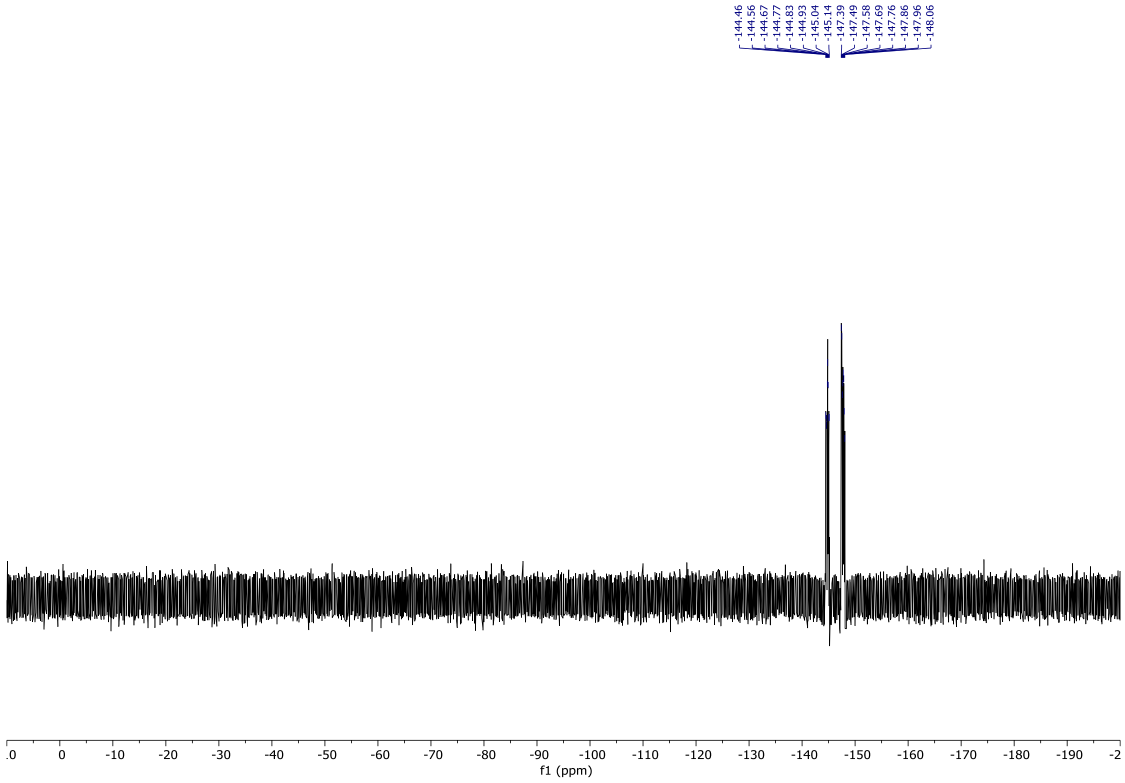
-144.46
-144.56
-144.67
-144.77
-144.83
-144.93
-145.04
-145.14
-147.39
-147.49
-147.58
-147.69
-147.76
-147.86
-147.96
-148.06
f1 (ppm)

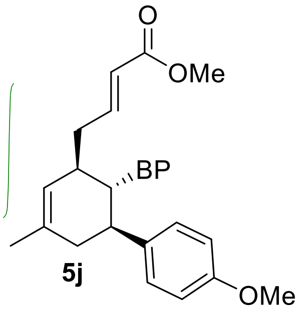
O
OMe
BP
5j
OMe

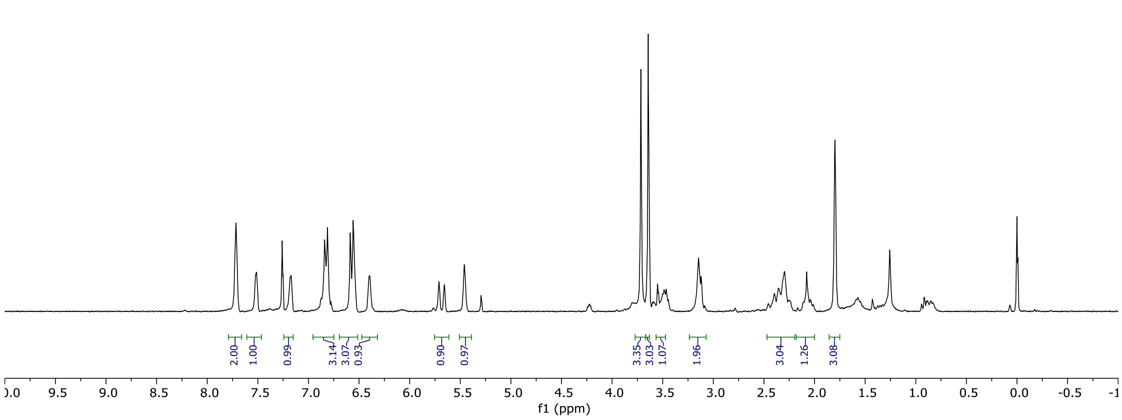
2.00
1.00
0.99
3.14
3.07
0.93
0.90
0.97
3.35
3.03
1.07
1.96
3.04
1.26
3.08
f1 (ppm)

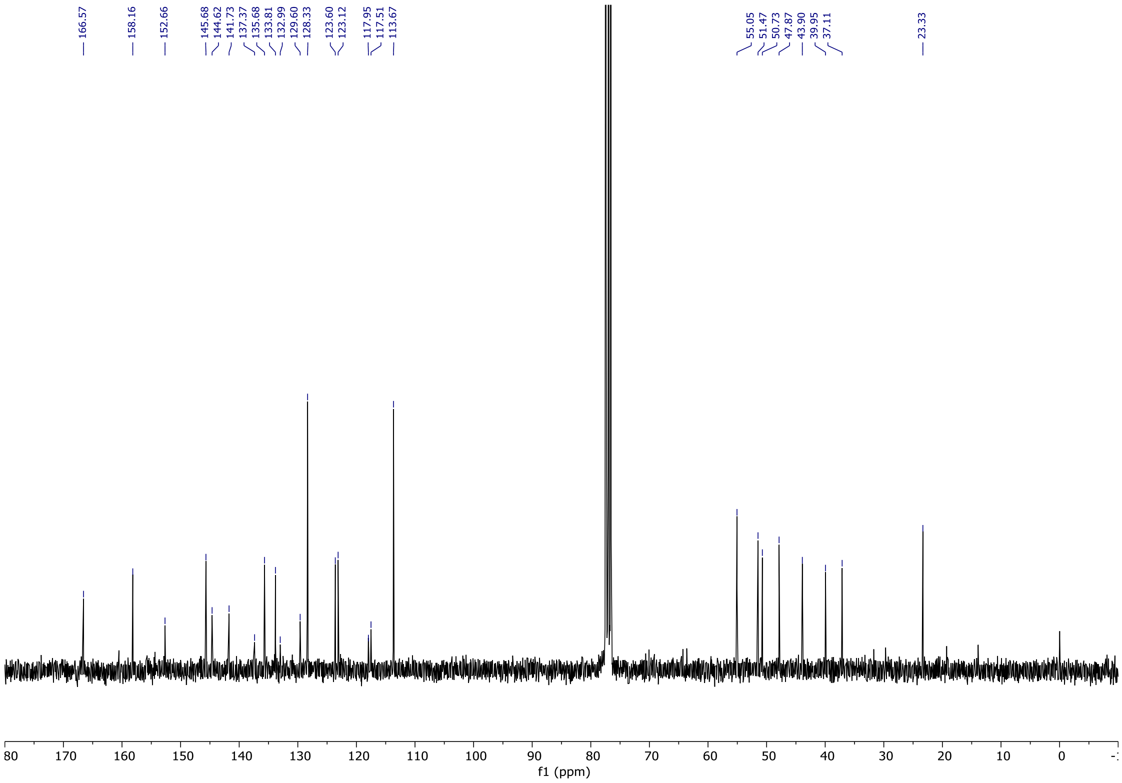
166.57
158.16
152.66
145.68
144.62
141.73
137.37
135.68
133.81
132.99
129.60
128.33
123.60
123.12
117.95
117.51
113.67
55.05
51.47
50.73
47.87
43.90
39.95
37.11
23.33
f1 (ppm)

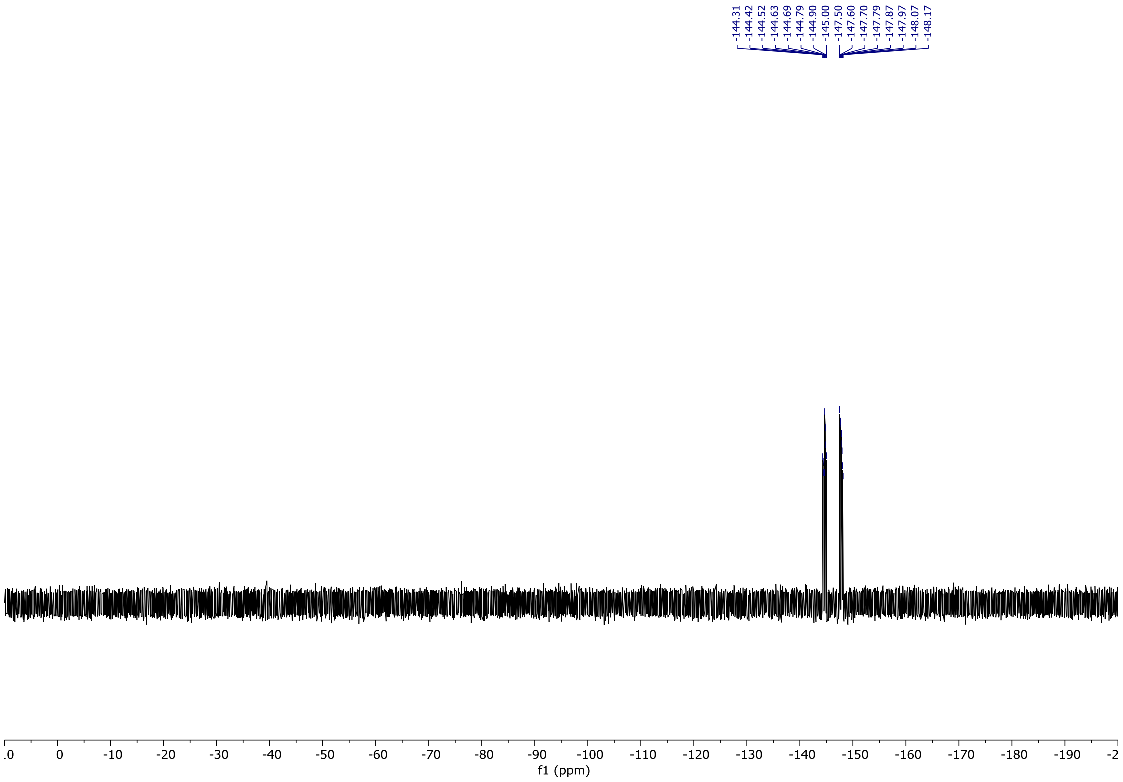
-144.31
-144.42
-144.52
-144.63
-144.69
-144.79
-144.90
-145.00
-147.50
-147.60
-147.70
-147.79
-147.87
-147.97
-148.07
-148.17
f1 (ppm)

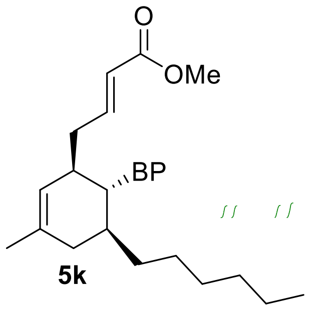

O
OMe
BP

5k

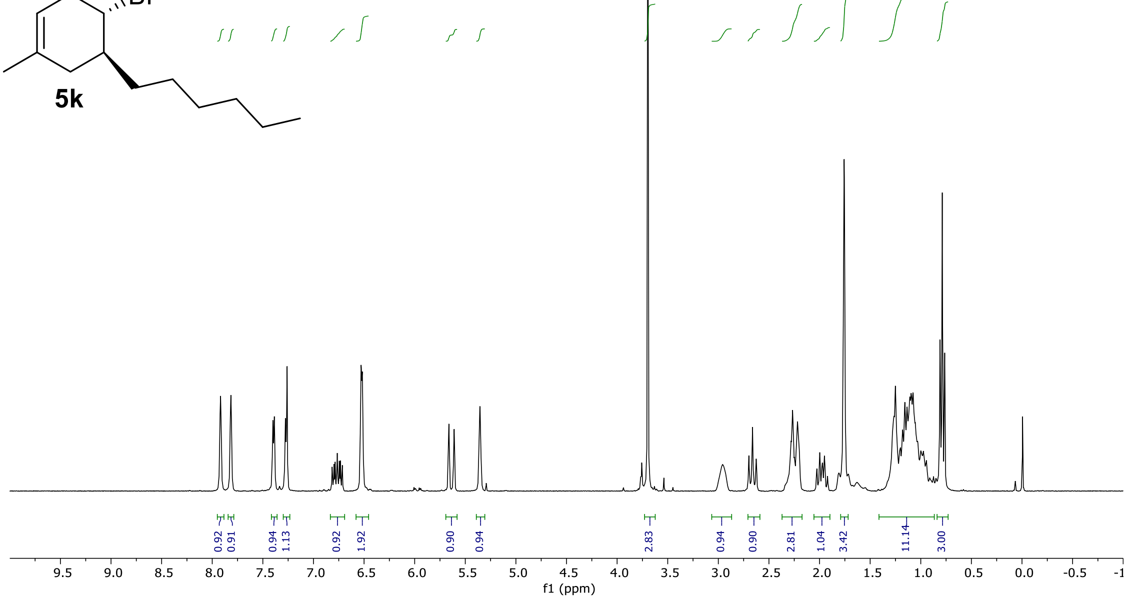

0.92
0.91
0.94
1.13
0.92
1.92
0.90
0.94
2.83
0.94
0.90
2.81
1.04
3.42
11.14
3.00
f1 (ppm)

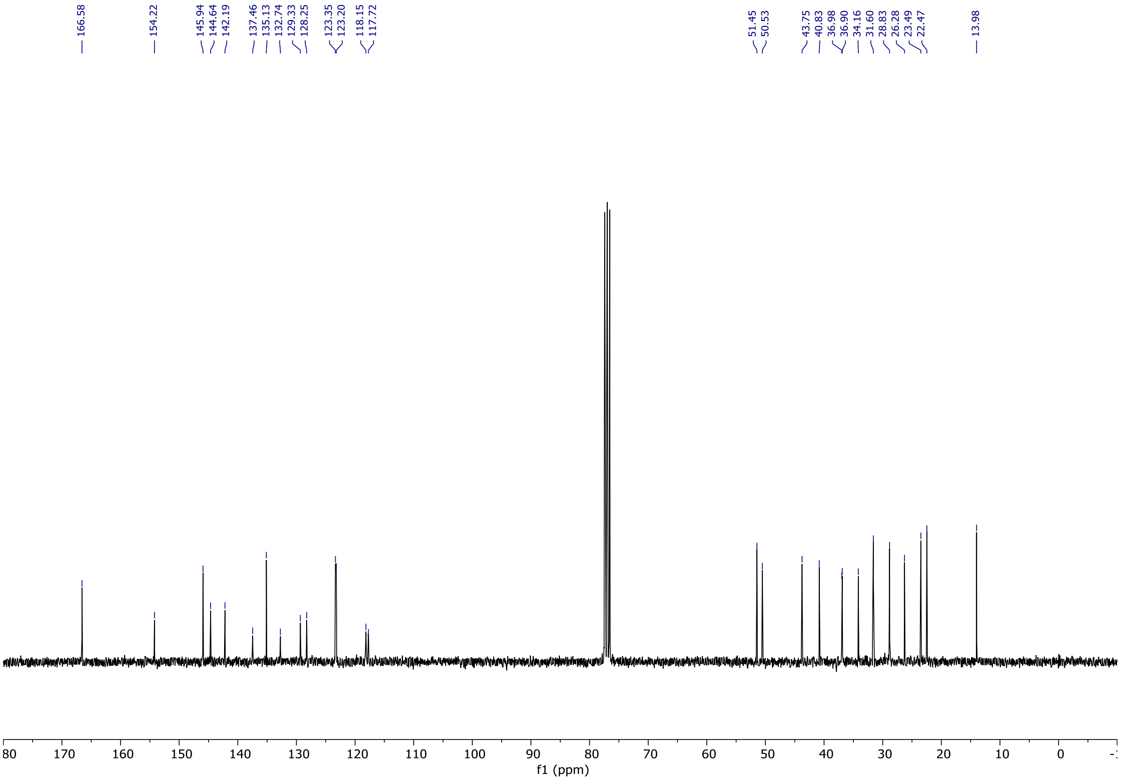

166.58
154.22
145.94
144.64
142.19
137.46
135.13
132.74
129.33
128.25
123.35
123.20
118.15
117.72
51.45
50.53
43.75
40.83
36.98
36.90
34.16
31.60
28.83
26.28
23.49
22.47
13.98
f1 (ppm)

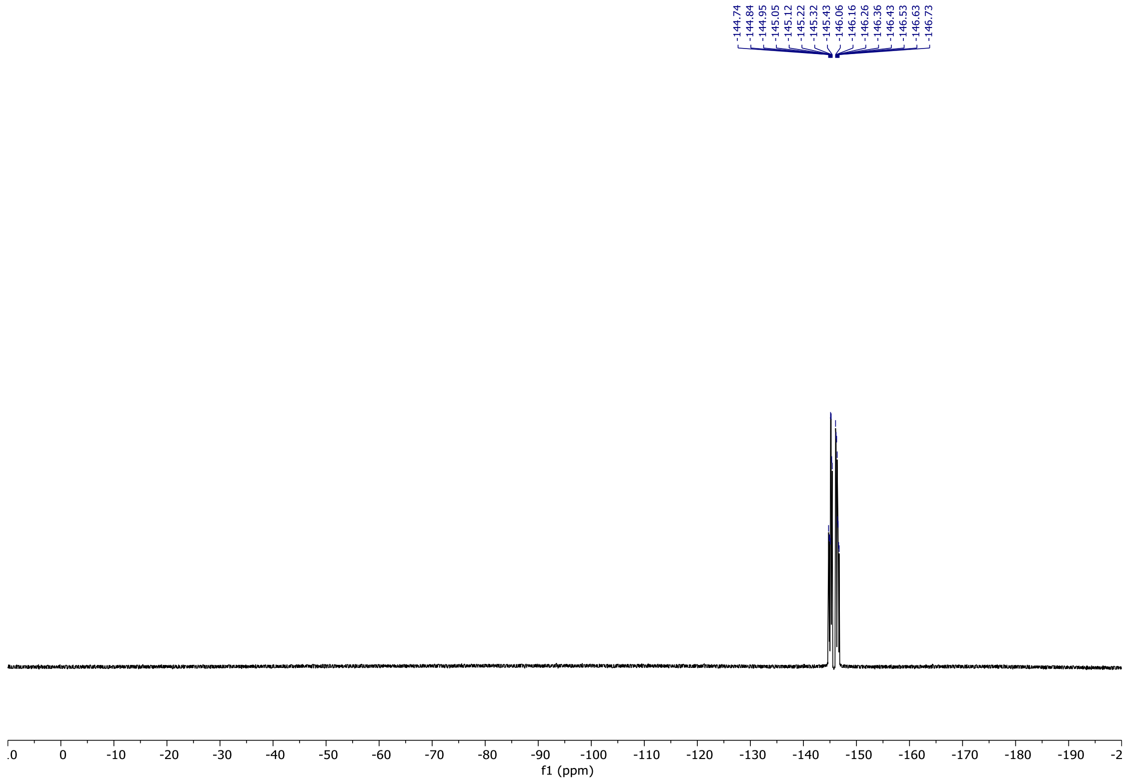
-144.74
-144.84
-144.95
-145.05
-145.12
-145.22
-145.32
-145.43
-146.06
-146.16
-146.26
-146.36
-146.43
-146.53
-146.63
-146.73
f1 (ppm)

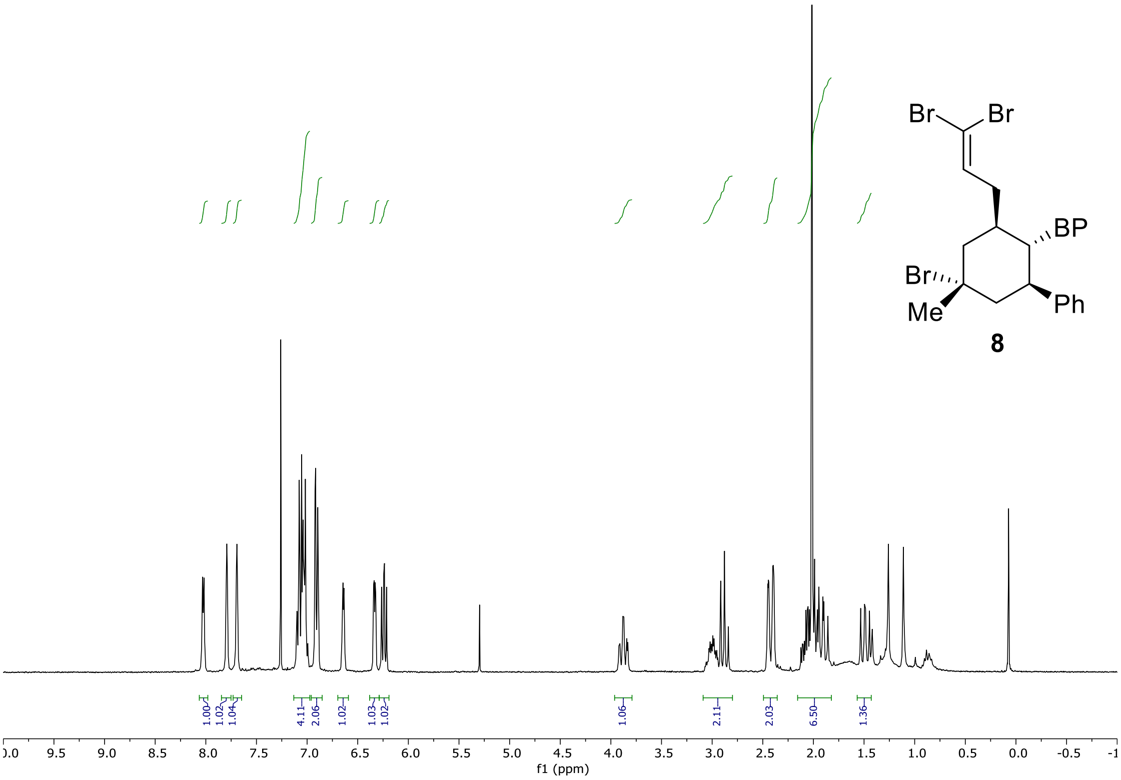

1.00
1.02
1.04
4.11
2.06
1.02
1.03
1.02
1.06
2.11
2.03
6.50
1.36
f1 (ppm)
Br
Br
BP
Br
Me
Ph
8

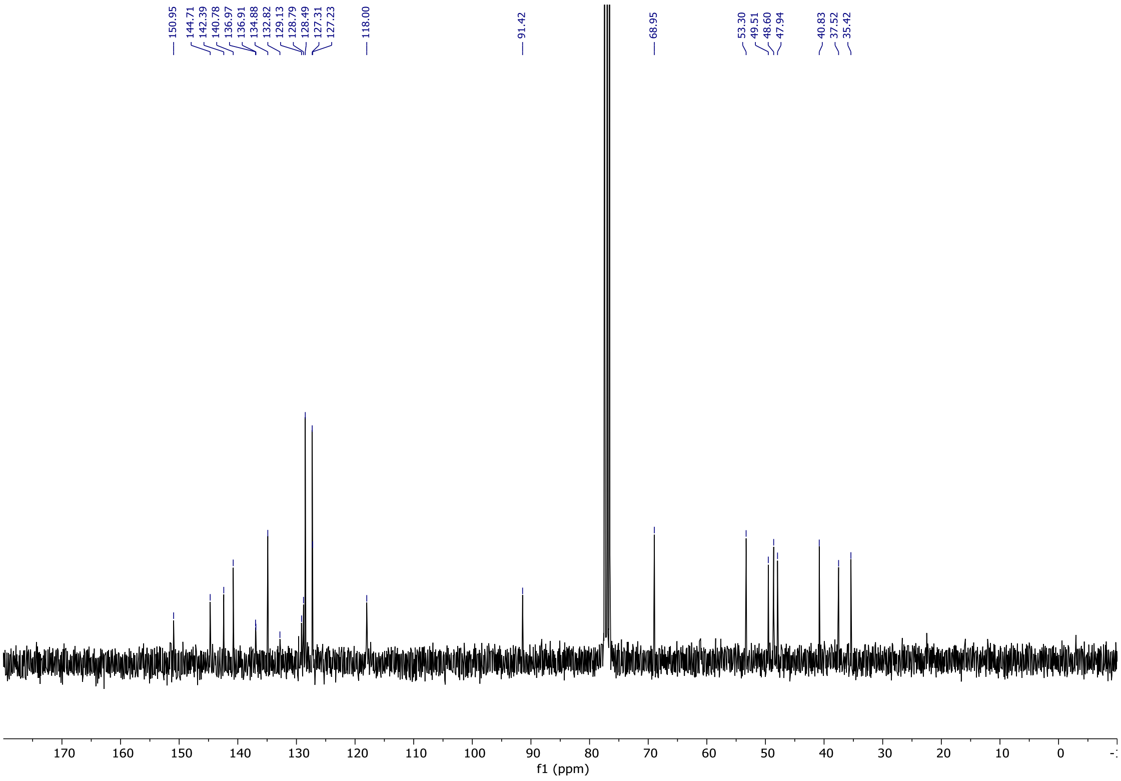

150.95
144.71
142.39
140.78
136.97
136.91
134.88
132.82
129.13
128.79
128.49
127.31
127.23
118.00
91.42
68.95
53.30
49.51
48.60
47.94
40.83
37.52
35.42
f1 (ppm)

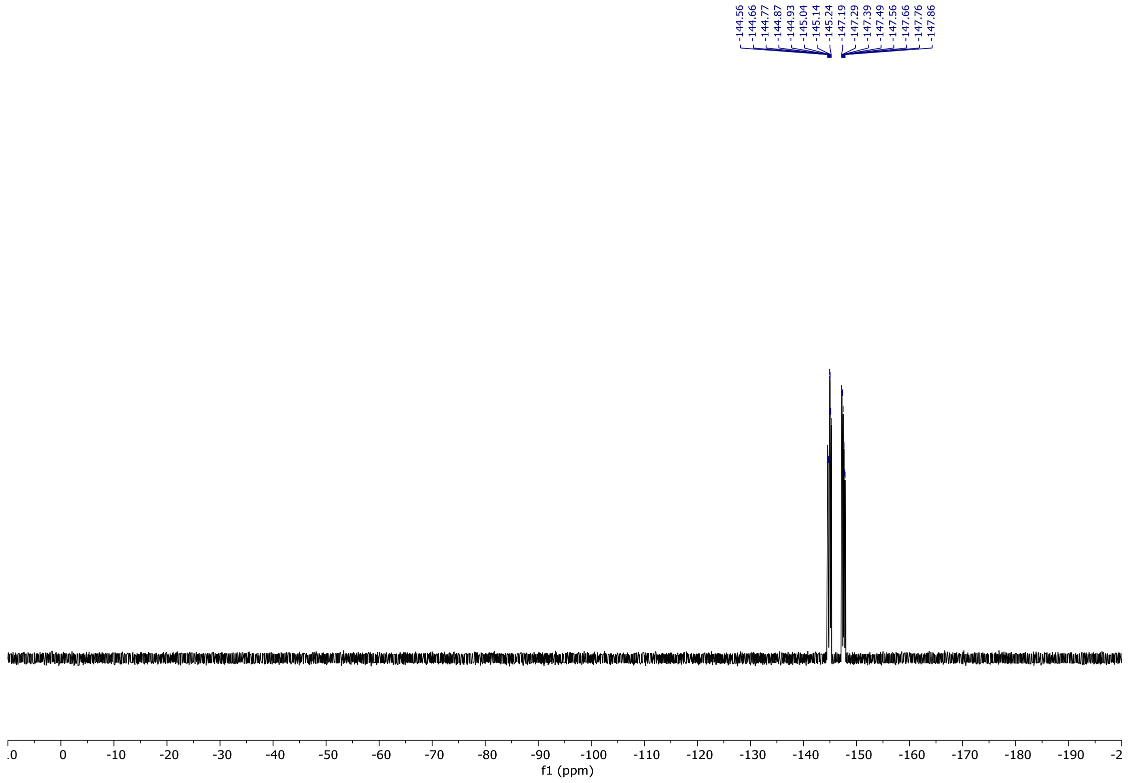
-144.56
-144.66
-144.77
-144.87
-144.93
-145.04
-145.14
-145.24
-147.19
-147.29
-147.39
-147.49
-147.56
-147.66
-147.76
-147.86
f1 (ppm)

9. SFC CHROMATOGRAPHS

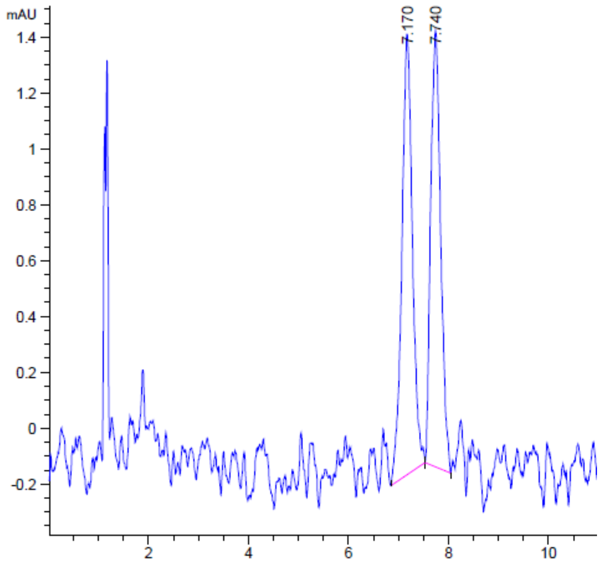

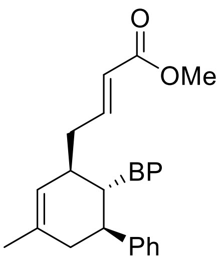

5a

Peak #	RetTime [min]	Type	Width [min]	Area [mAU*s]	Height [mAU]	Area %
1	7.170	BB	0.1989	23.12912	1.57853	51.7958
2	7.740	BB	0.1808	21.52529	1.56186	48.2042

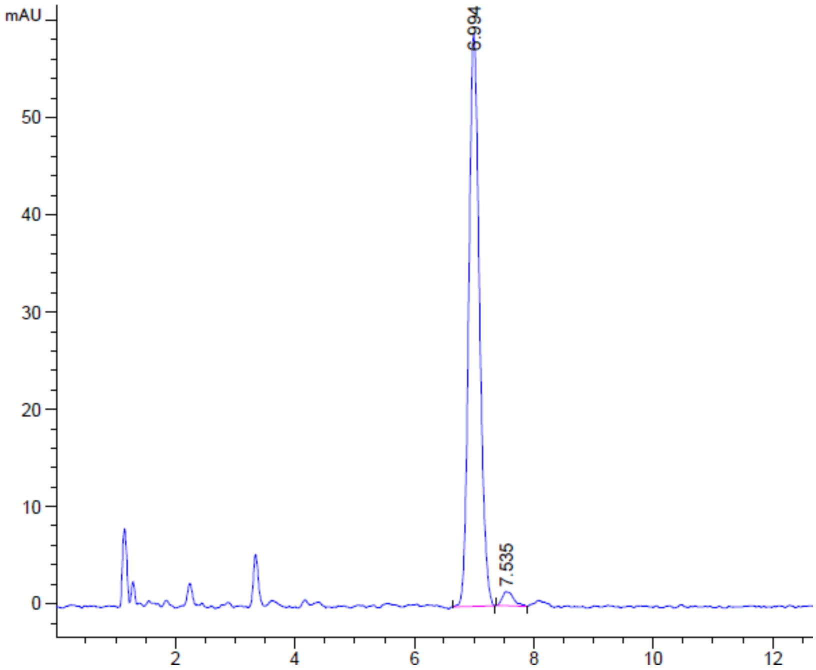

Peak #	RetTime [min]	Type	Width [min]	Area [mAU*s]	Height [mAU]	Area %
1	6.994	BB	0.1936	733.52258	58.78619	97.4015
2	7.535	BB	0.1714	19.56873	1.45043	2.5985

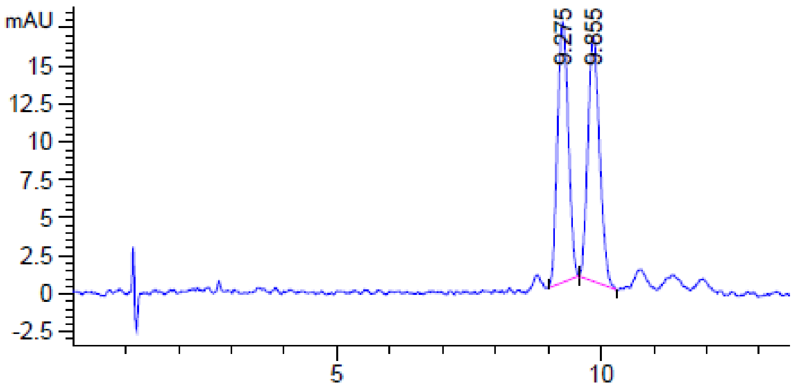

Peak #	RetTime [min]	Type	Width [min]	Area [mAU*s]	Height [mAU]	Area %
1	9.273	BB	0.2385	638.62671	43.70446	49.3486
2	9.853	BB	0.2444	655.48596	41.03282	50.6514

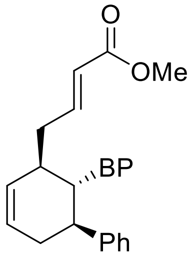

5b

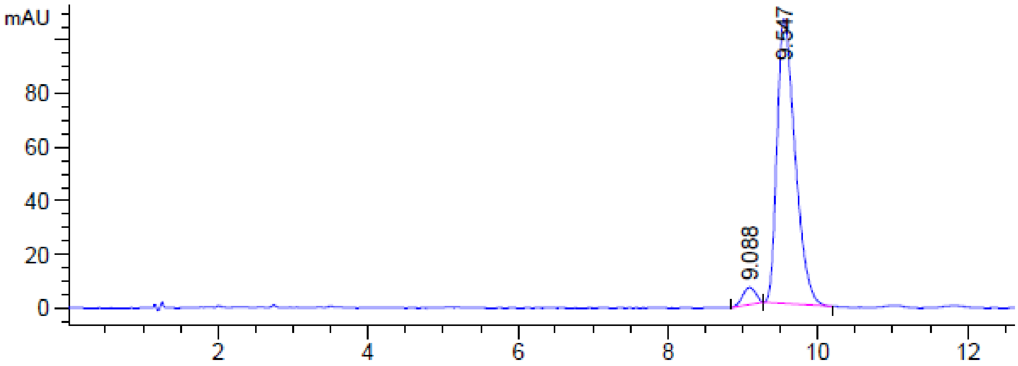

Peak #	RetTime [min]	Type	Width [min]	Area [mAU*s]	Height [mAU]	Area %
1	9.088	BB	0.1966	77.41145	6.42494	4.1707
2	9.547	BB	0.2573	1778.66394	106.22799	95.8293

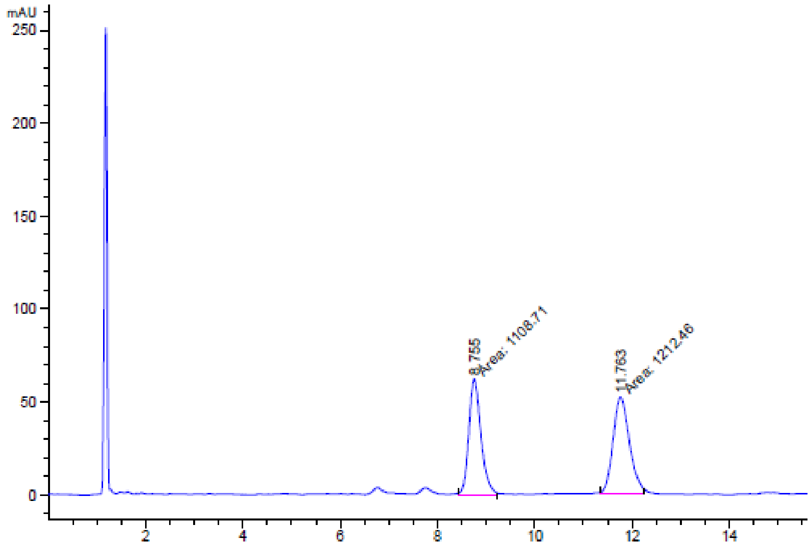

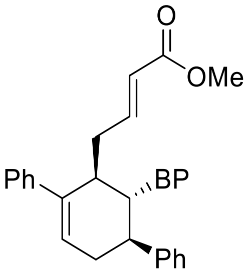

5c

Peak #	RetTime [min]	Type	Width [min]	Area [mAU*s]	Height [mAU]	Area %
1	8.755	MM	0.2935	1108.71094	62.94939	47.7651
2	11.763	MM	0.3887	1212.46436	51.99137	52.2349

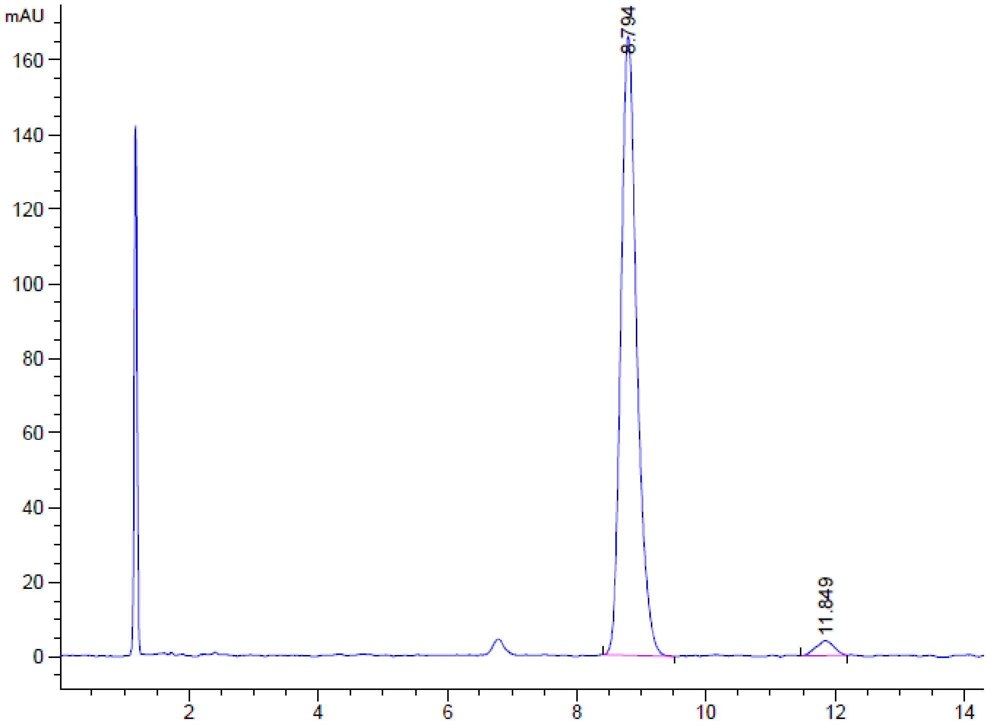

Peak #	RetTime [min]	Type	Width [min]	Area [mAU*s]	Height [mAU]	Area %
1	8.794	BB	0.2625	2849.28833	165.83229	97.4399
2	11.849	BB	0.2688	74.86259	3.81703	2.5601

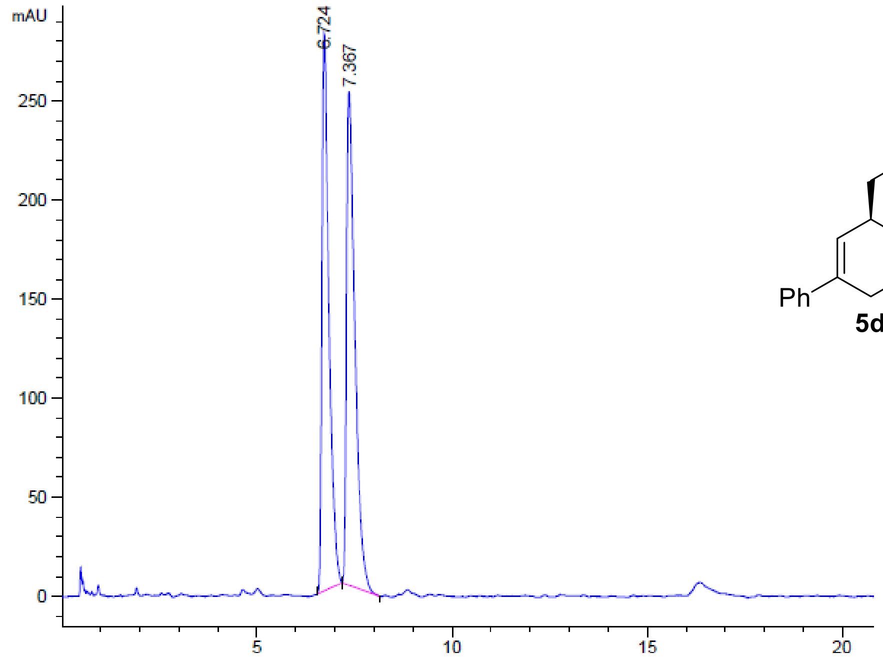

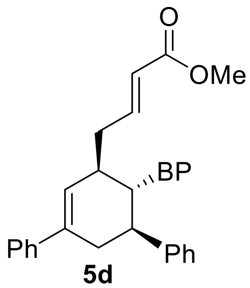

5d

Peak #	RetTime [min]	Type	Width [min]	Area [mAU*s]	Height [mAU]	Area %
1	6.724	BB	0.1854	3556.15210	281.41531	49.3022
2	7.367	BB	0.2171	3656.81714	249.38553	50.6978

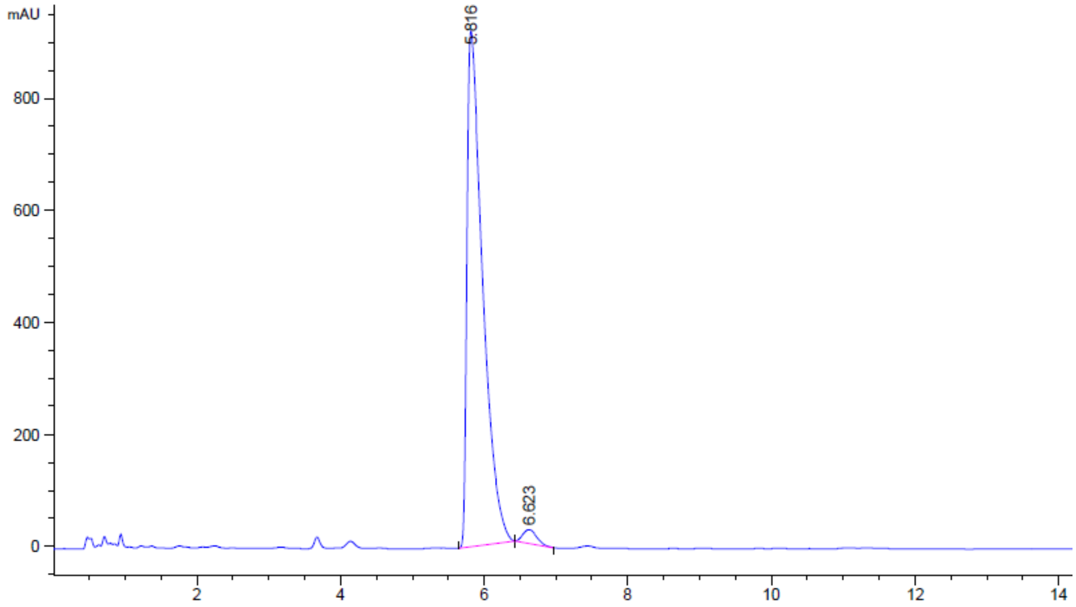

Peak #	RetTime [min]	Type	Width [min]	Area [mAU*s]	Height [mAU]	Area %
1	5.816	BB	0.2288	1.39684e4	921.49664	97.6874
2	6.623	BB	0.2094	330.67609	24.83158	2.3126

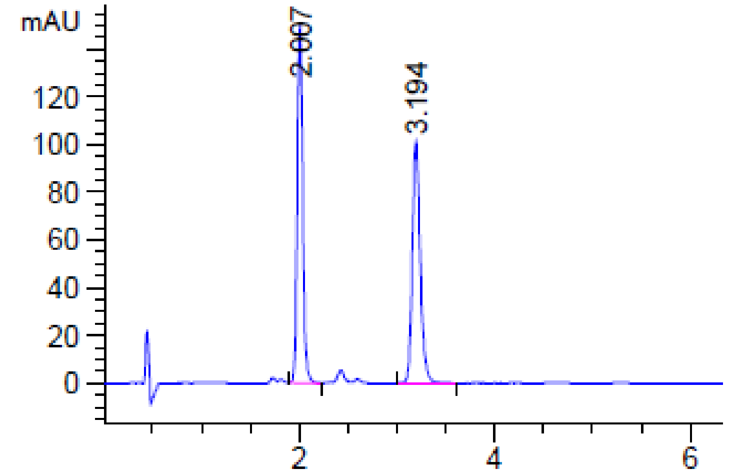

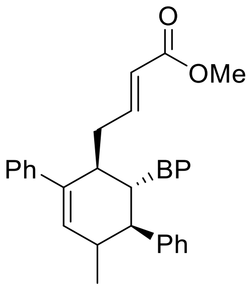

5e

Peak #	RetTime [min]	Type	Width [min]	Area [mAU*s]	Height [mAU]	Area %
1	2.007	BB	0.0591	582.14606	150.60413	50.1999
2	3.194	BB	0.0886	577.50952	101.13416	49.8001

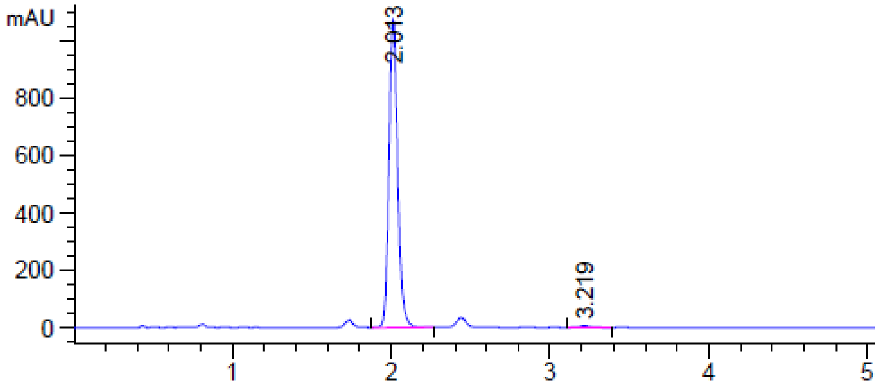

Peak #	RetTime [min]	Type	Width [min]	Area [mAU*s]	Height [mAU]	Area %
1	2.013	BB	0.0582	4087.44824	1078.05249	99.2454
2	3.219	BB	0.0820	31.07694	5.84950	0.7546

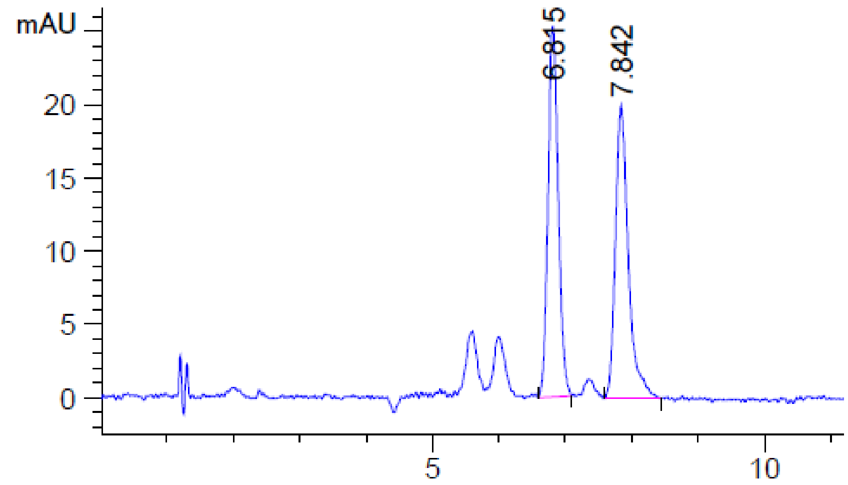

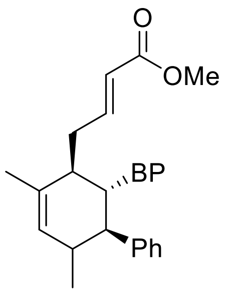

5f

Peak #	RetTime [min]	Type	Width [min]	Area [mAU*s]	Height [mAU]	Area %
1	6.815	BB	0.1635	272.15576	25.29503	49.8545
2	7.842	BB	0.2072	273.74457	20.05802	50.1455

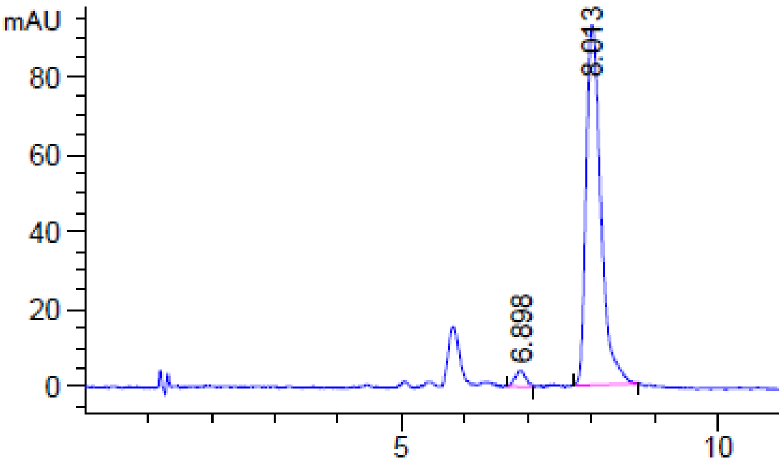

Peak #	RetTime [min]	Type	Width [min]	Area [mAU*s]	Height [mAU]	Area %
1	6.898	BB	0.1731	50.69524	4.13314	3.2699
2	8.013	BB	0.2519	1499.68286	93.12270	96.7301

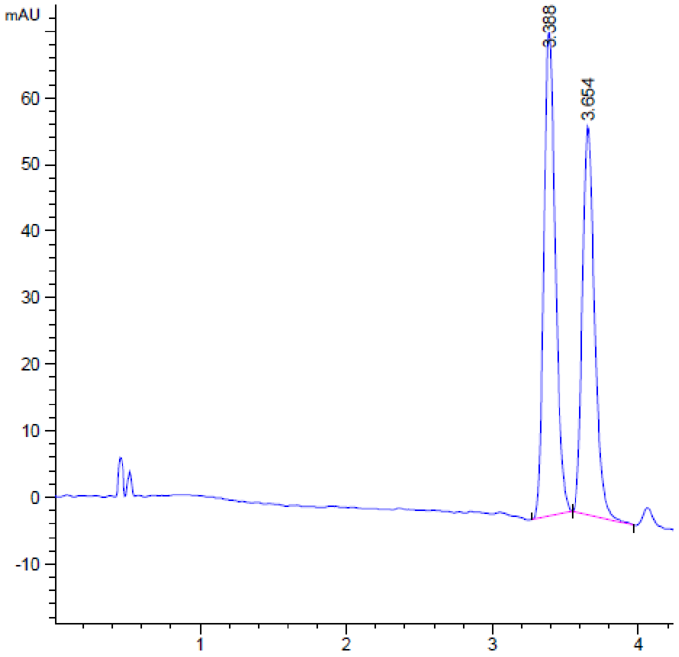

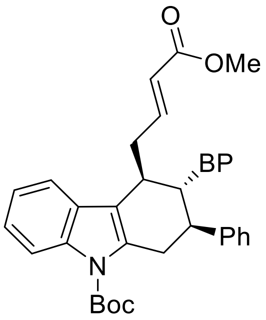

5g

Peak #	RetTime [min]	Type	Width [min]	Area [mAU*s]	Height [mAU]	Area %
1	3.388	BB	0.0840	399.34598	72.81319	53.8769
2	3.654	BB	0.0903	341.87314	58.40452	46.1231

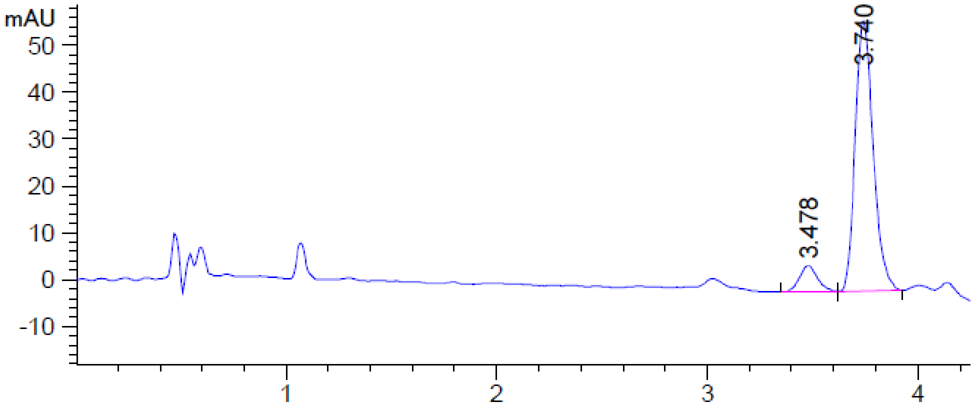

Peak #	RetTime [min]	Type	Width [min]	Area [mAU*s]	Height [mAU]	Area %
1	3.478	BB	0.0931	33.13215	5.59410	8.8712
2	3.740	BB	0.0907	340.34708	57.77324	91.1288

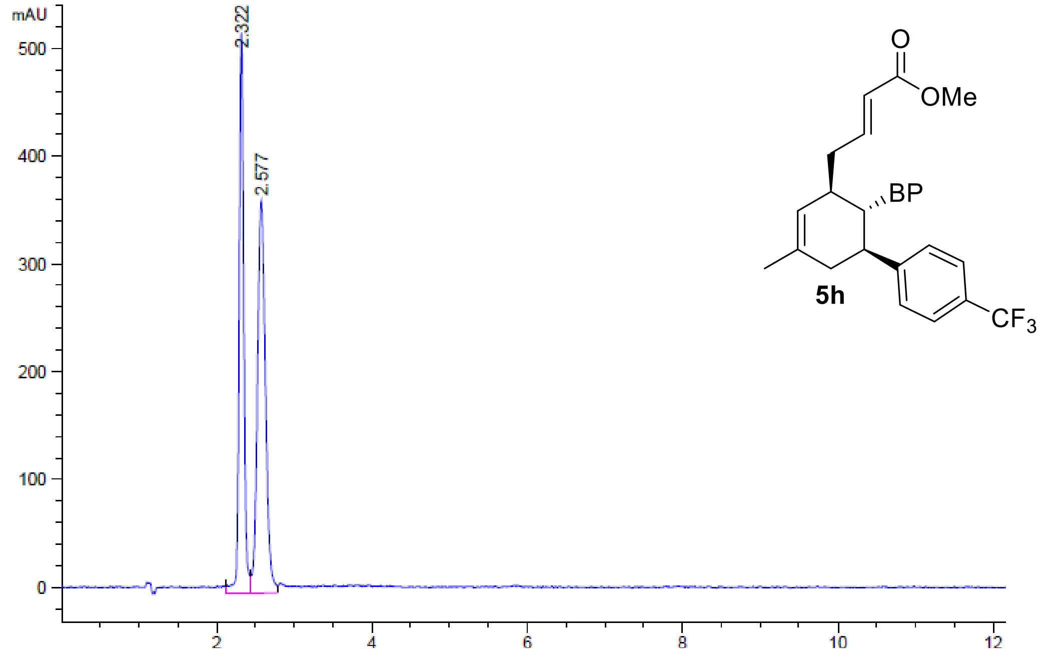

Peak #	RetTime [min]	Type	Width [min]	Area [mAU*s]	Height [mAU]	Area %
1	2.322	VV	0.0603	2065.04321	520.43439	43.7581
2	2.577	VV	0.1131	2654.17920	364.02188	56.2419

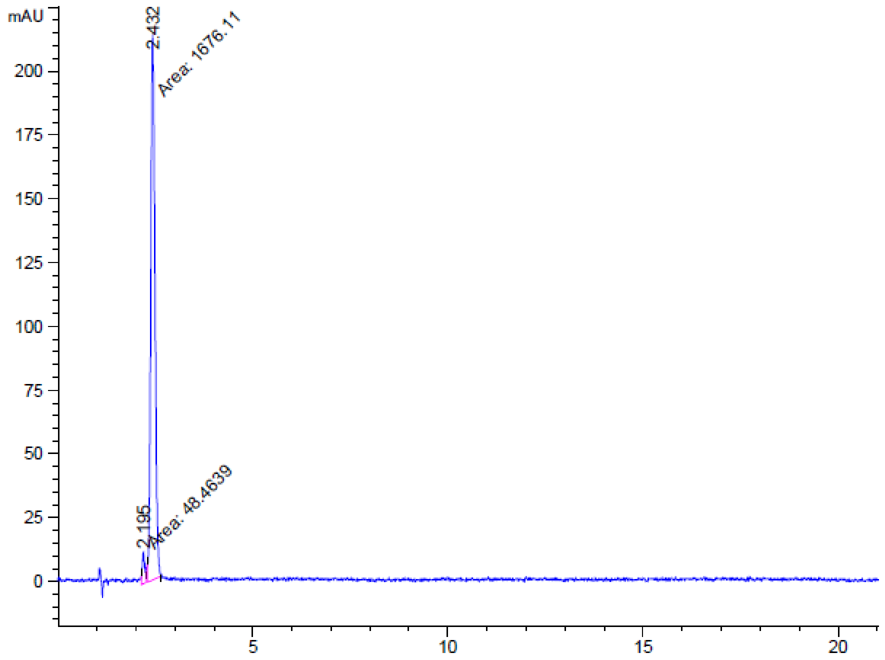

Peak #	RetTime [min]	Type	Width [min]	Area [mAU*s]	Height [mAU]	Area %
1	2.195	MF	0.0661	48.46390	12.21202	2.8102
2	2.432	FM	0.1305	1676.11328	214.04582	97.1898

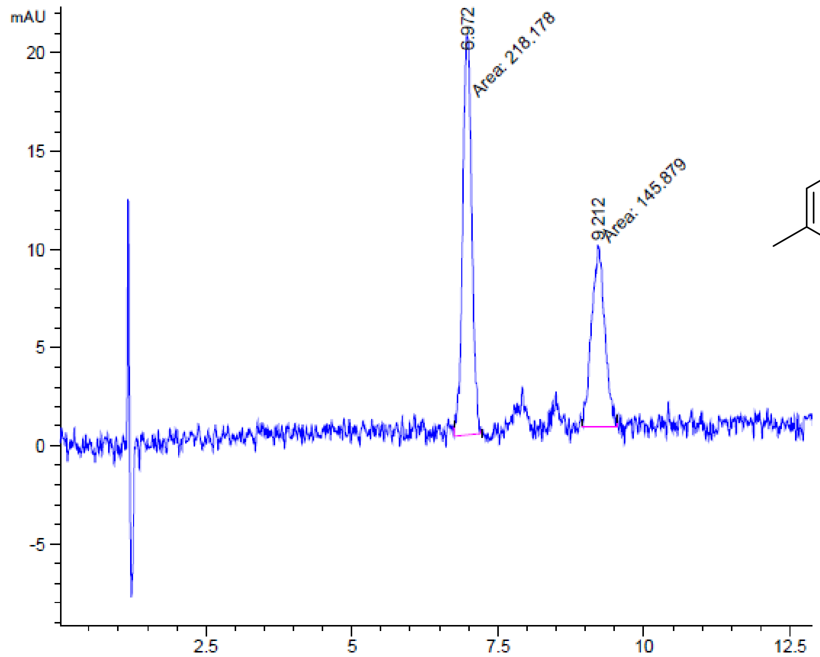

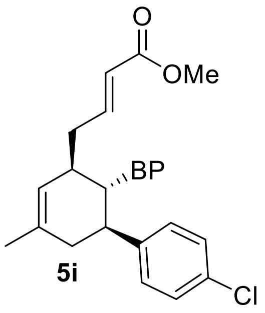

5i

Peak #	RetTime [min]	Type	Width [min]	Area [mAU*s]	Height [mAU]	Area %
1	6.972	MM	0.1786	218.17836	20.36241	59.9297
2	9.212	MM	0.2628	145.87903	9.25128	40.0703

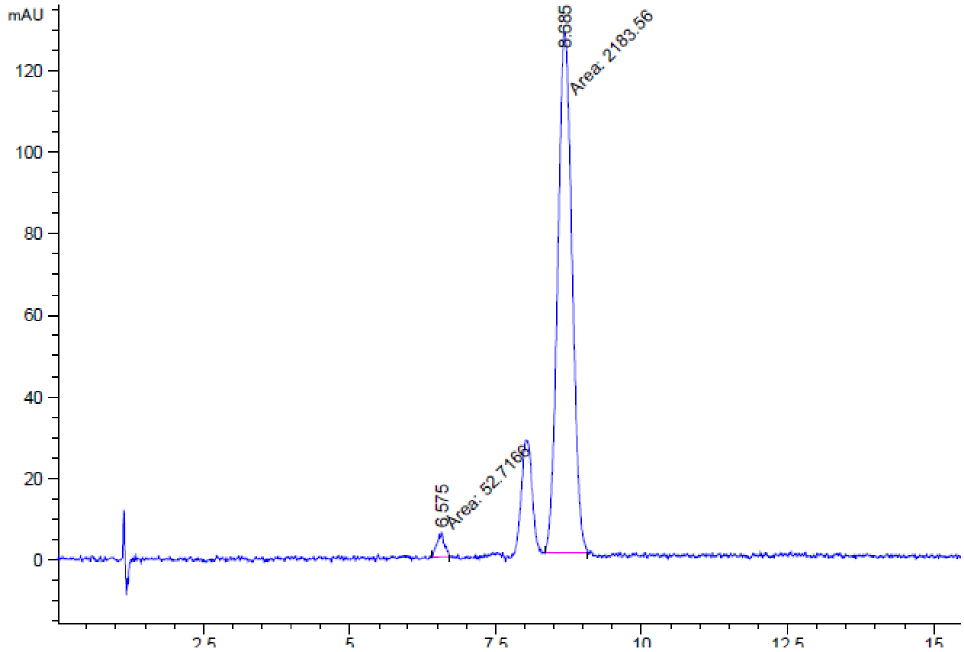

Peak #	RetTime [min]	Type	Width [min]	Area [mAU*s]	Height [mAU]	Area %
1	6.575	MM	0.1478	52.71663	5.94552	2.3573
2	8.685	MM	0.2855	2183.55615	127.47028	97.6427

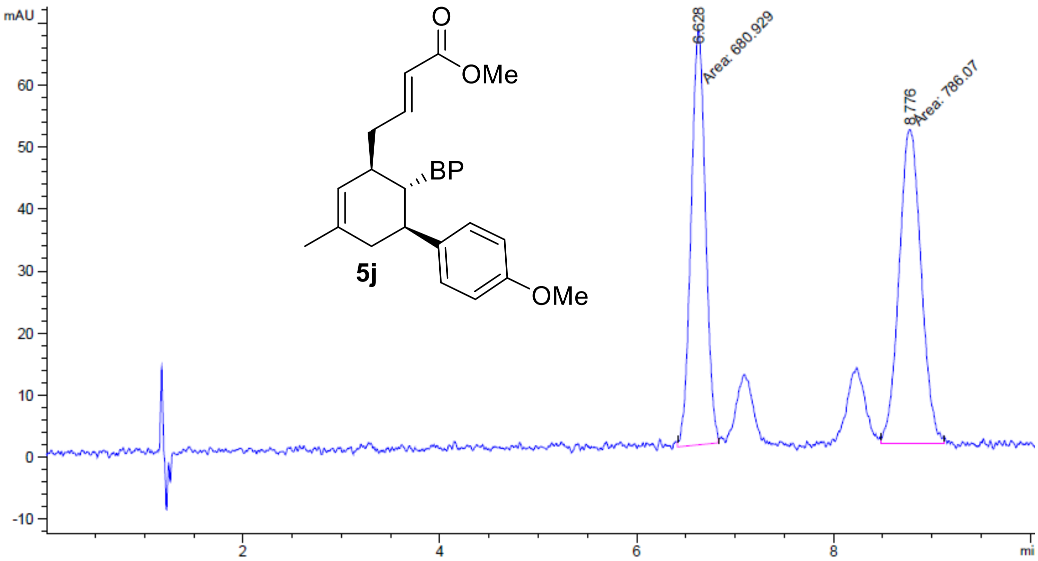

Peak #	RetTime [min]	Type	Width [min]	Area [mAU*s]	Height [mAU]	Area %
1	6.628	MM	0.1701	680.92889	66.71729	46.4165
2	8.776	MM	0.2593	786.07001	50.52860	53.5835

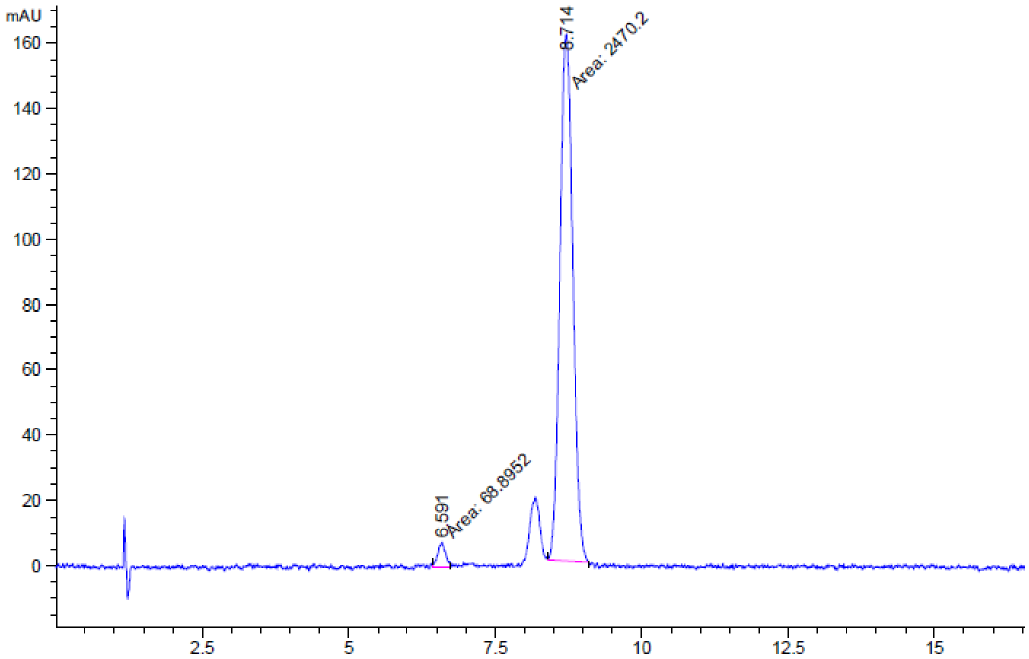

Peak #	RetTime [min]	Type	Width [min]	Area [mAU*s]	Height [mAU]	Area %
1	6.591	MM	0.1532	68.89517	7.49732	2.7134
2	8.714	MM	0.2556	2470.20288	161.09389	97.2866

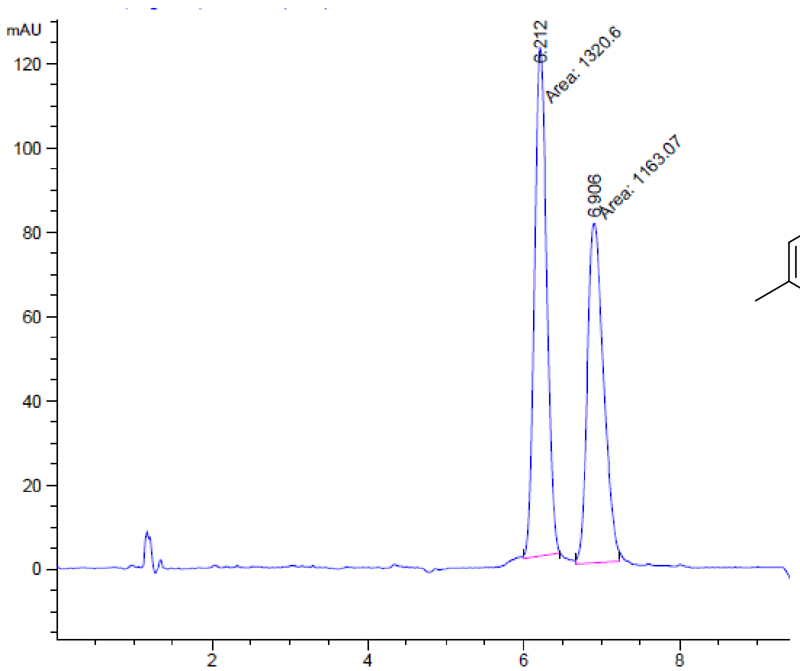

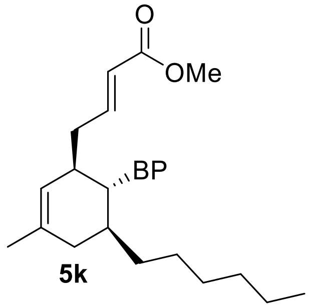

5k

Peak #	RetTime [min]	Type	Width [min]	Area [mAU*s]	Height [mAU]	Area %
1	6.212	MM	0.1824	1320.59534	120.64341	53.1712
2	6.906	MM	0.2403	1163.07031	80.66151	46.8288

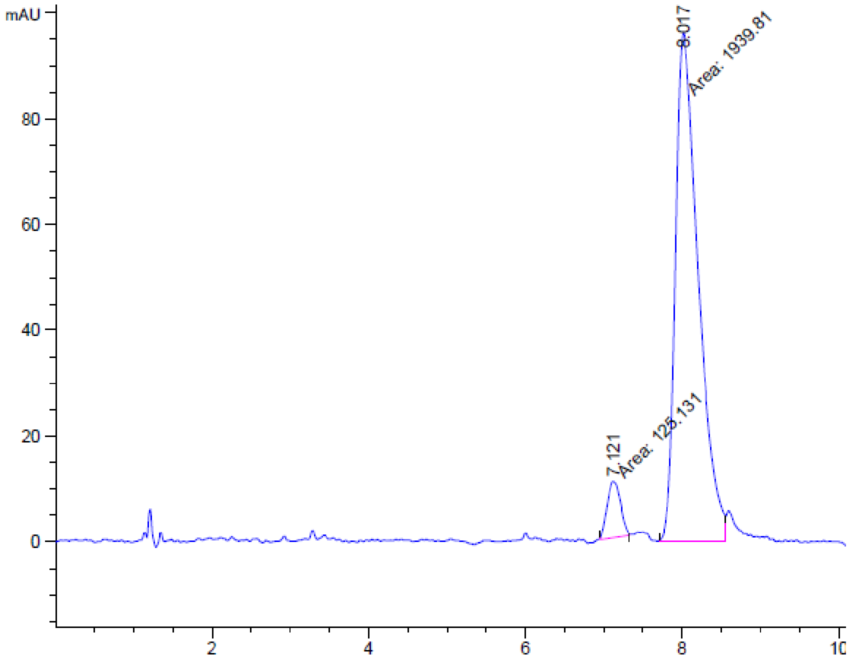

Peak #	RetTime [min]	Type	Width [min]	Area [mAU*s]	Height [mAU]	Area %
1	7.121	MM	0.1964	125.13101	10.61758	6.0598
2	8.017	MF	0.3368	1939.81079	95.98139	93.9402

10. Computational details

All the calculations were performed using M06-2X Minnesota functional that is specially designed to account for dispersive interactions and broadly used for mechanistic studies.[12,13] For geometry optimizations, orbital energies, harmonic frequency calculations, thermodynamic corrections and intrinsic reaction coordinate (IRC) calculations we used Pople's double-ξ basis set: 6-31G(d, p) which includes polarization functions. Harmonic vibrational frequencies were computed to characterize minima and transition states (TS) and IRCs to verify connectivity between TSs and adjacent minima.

More accurate values for the final energies were computed by means of single point calculations over the geometries previously optimized. A larger basis set was used for this calculations: 6-31+G(d, p) adding a set of diffuse functions for heavy atoms. The effect of the solvent (p-xylene) was also taken into account using the SMD continuum solvation model.[14]

All the above calculations were performed with the Gaussian09 suite of programs.[15]

11. Intrinsic reaction coordinate plots

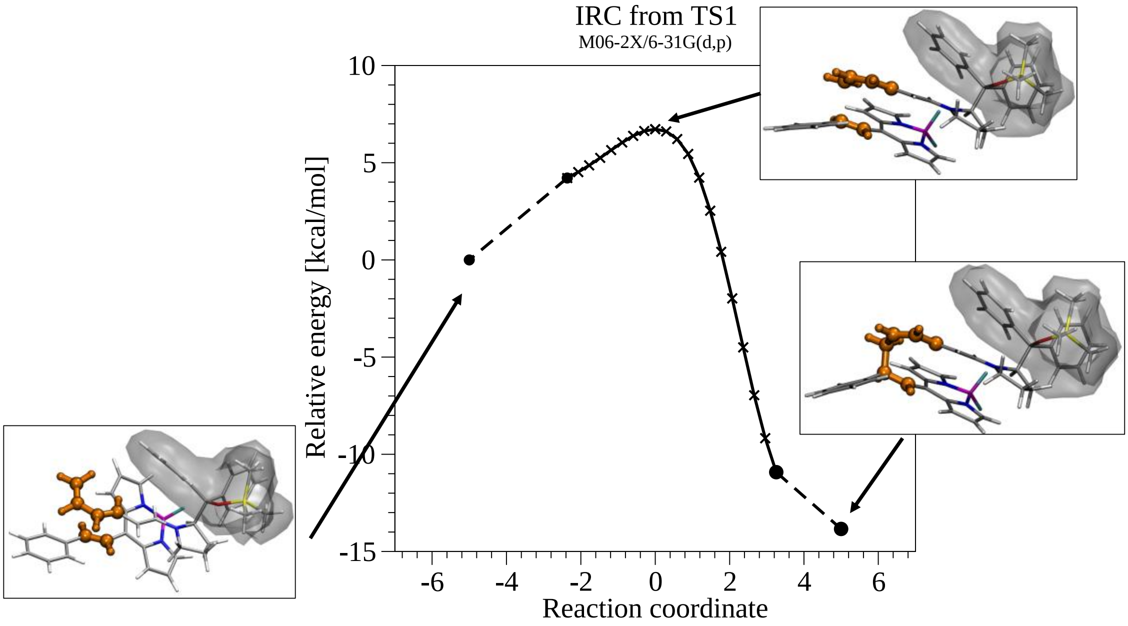

Figure S2: Intrinsic reaction coordinate curve starting from TS_1: first C-C bond formation. The crosses are the IRC pints and the black dots correspond to the energy of the last point of the IRC (forward and reverse) and the energy of the optimized structure of this point, that corresponds to the PAC (left hand side) and int1 (right hand side).

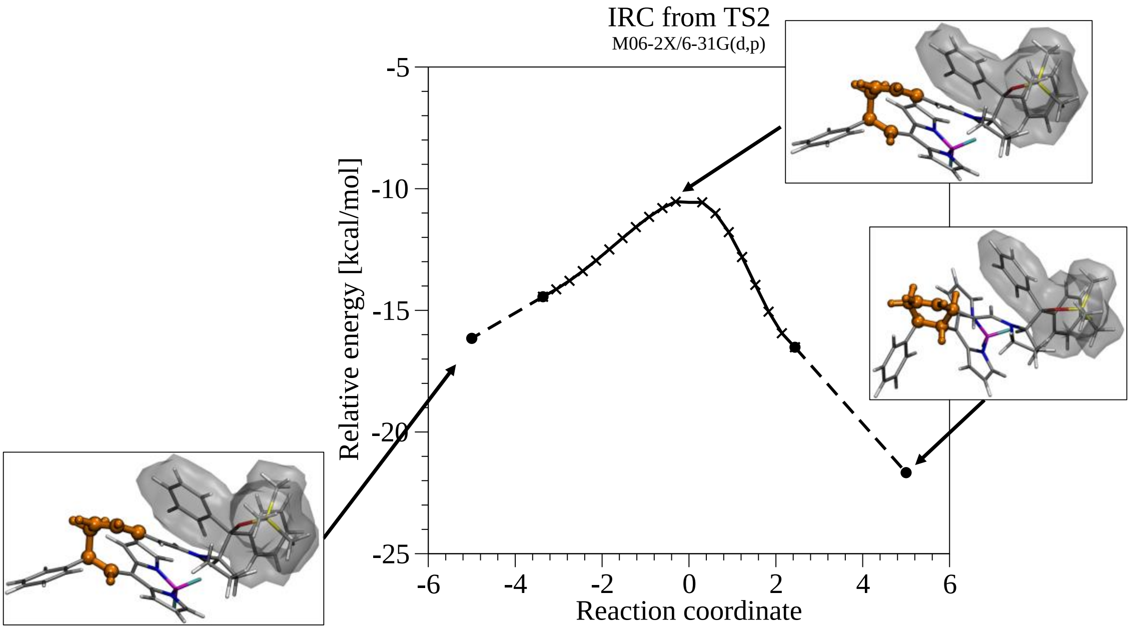

Figure S3: Intrinsic reaction coordinate curve starting from TS_2: second C-C bond formation. The crosses are the IRC pints and the black dots correspond to the energy of the last point of the IRC (forward and reverse) and the energy of the optimized structure of this point, that corresponds to the int1 (left hand side) and product (right hand side).

12. Frontier molecular orbitals

The frontier molecular orbital (FMO) theory is a widely used model to describe chemical reactivity, specially for pericyclic reactions.[16] The frontier molecular orbitals are the highest occupied and lowest unoccupied molecular orbitals (HOMO and LUMO) respectively. The electrons coming from or moving to these orbitals are the most prone to participate in a reaction. Therefore, analyzing the energies, shapes and localizations of these orbitals it is possible to explain and predict reactivity and selectivity.

For this reaction the relevant orbitals are the HOMO of the nucleophile (trienamine **1b**), and the LUMO of the electrophile, the double bond with the BODIPY as EWG (**2**), that is the orbital receiving electron density from the nucleophile. Trienamines are good as nucleophiles since the energy of their HOMOs is relatively high, however, simple alkenes have relatively high-energy LUMOs and therefore they are not good reactants on these kind of reactions. By conjugating the double bond with an electron-withdrawing group (the BODIPY) the LUMO energy is lowered favoring the interaction with the trienamine HOMO. This HOMO-LUMO interaction results in an energetically favorable bond formation. Thus, the closest the LUMO energy to the trienamine HOMO energy the more favorable the reaction.

Table S1: HOMO energy for trienamine **1b** and BODIPYs **2a**. The energies for LUMO orbitals of nitrostyrene **9**, styrene, and alkene are also shown for comparison.

	Frontier Orbitals Energies [eV]		
**Reactant**	**HOMO**	**LUMO**	**GAP**
Trienamine **1b**	-5.49		
Bodipy **2a**		-2.30	3.19
Nitrostyrene **9**		-1.58	3.91
Styrene		0.15	5.64
Alkene		1.70	7.19

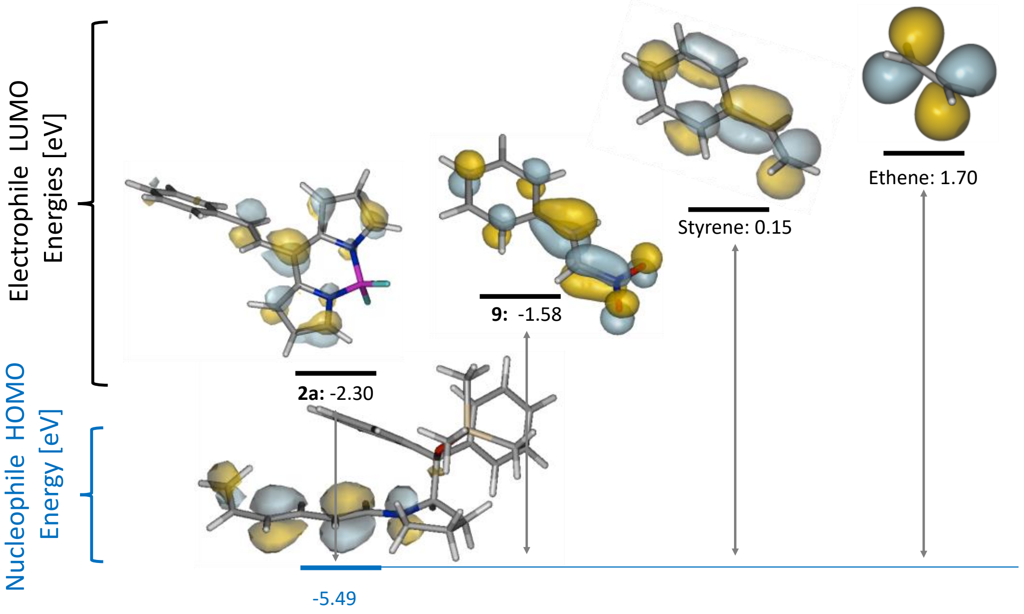

Figure S4: Orbitals and orbital energies for trienamine **1b** HOMO and BODIPYs **2a** LUMO. The energies for LUMO orbitals of nitrostyrene **9**, styrene, and alkene are also shown for comparison.

13. References

1. (a) Y. Wang, M. Gu *Anal. Chem*. **2010**, *82*, 7055; (b) Y. Wang *Methods for Operating MS Instrument Systems*, United States Patent No. 6,983,213, **2006**; (c) N. Ochiai, K. Sasamoto, K. MacNamara *Journal of Chromatography A*, **2012**, *1270*, 296; (d) H. -P. Ho, R. -Y. Lee, C. -Y. Chen, S. -R. Wang, Z. -G. Li, M. -R. Lee *Rapid Commun. Mass Spectrom.*, **2011**, *25*, 25.
2. G. Battistuzzi, S. Cacchi, G. Fabrizi, *Org. Lett*., **2003**, *5*, 777.
3. Z. Jia, Q. Zhou, Q. Zhou, P. Chen, Y. Chen *Angew. Chem. Int. Ed.*, **2011**, *50*, 8638.
4. L. Prieto, G. Talavera, U. Uria, E. Reyes, J. L. Vicario, L. Carrillo *Chem. Eur. J.*, **2014**, *20*, 2145.
5. Y. Liu, M. Nappi, E. Arceo, S. Vera, P. Melchiorre *J. Am. Chem. Soc.*, **2011**, *133*, 15212.
6. M. J. Plater, S. Aiken, G. Bourhill *Tetrahedron*, **2002**, *58,* 2405.
7. V. Leen, P. Yuan, L. Wang, N. Boens, W. Dehaen *Org. Lett*., **2012**, *14*, 6150.
8. A. Orte, E. Debroye, M. J. Ruedas-Rama, E. García-Fernandez, D. Robinson, L. Crovetto, E. M. Talavera, J. M. Alvarez-Pez, V. Leen, B. Verbelen, L. Cunha Dias de Rezende, W. Dehaen, J. Hofkens, M. Van der Auweraer, N. Boens *RSC Adv.,* **2016**, *6*, 102899.
9. I. Arroyo, R. Hu, B. Z. Tang, F. Lopez, E. Peña-Cabrera *Tetrahedron,* **2011**, *67*, 7244.
10. T. V. Goud, A. Tutar, J. -F. Biellmann *Tetrahedron*, **2006**, *62*, 5084.
11. Ł. Albrecht, F. Cruz Acosta, A. Fraile, A. Albrecht, J. Christensen, K. A. Jorgensen *Angew. Chem. Int. Ed.*, **2012**, *51*, 9088.
12. Y. Zhao, D.G. Truhlar *Theor. Chem. Acc.,* **2008**, 120, 215.
13. Y. Zhao, D. G. Truhlar *Acc. Chem. Res.,* **2008**, 41, 157.
14. A. V. Marenich, C. J. Crame, D. G. Truhlar *J. Phys. Chem. B,* **2009**, *113*, 6378.
15. M. J. Frisch, G. W. Trucks, H. B. Schlegel, G. E. Scuseria, M. A. Robb, J. R. Cheeseman, G. Scalmani, V. Barone, G. A. Petersson, H. Nakatsuji, X. Li, M. Caricato, A. Marenich, J. Bloino, B. G. Janesko, R. Gomperts, B. Mennucci, H. P. Hratchian, J. V. Ortiz, A. F. Izmaylov, J. L. Sonnenberg, D. Williams-Young, F. Ding, F. Lipparini, F. Egidi, J. Goings, B. Peng, A. Petrone, T. Henderson, D. Ranasinghe, V. G. Zakrzewski, J. Gao, N. Rega, G. Zheng, W. Liang, M. Hada, M. Ehara, K. Toyota, R. Fukuda, J. Hasegawa, M. Ishida, T. Nakajima, Y. Honda, O. Kitao, H. Nakai, T. Vreven, K. Throssell, J. A. Montgomery, Jr., J. E. Peralta, F. Ogliaro, M. Bearpark, J. J. Heyd, E. Brothers, K. N. Kudin, V. N. Staroverov,

T. Keith, R. Kobayashi, J. Normand, K. Raghavachari, A. Rendell, J. C. Burant, S. S. Iyengar, J. Tomasi, M. Cossi, J. M. Millam, M. Klene, C. Adamo, R. Cammi, J. W. Ochterski, R. L. Martin, K. Morokuma, O. Farkas, J. B. Foresman, D. J. Fox *Gaussian 09, Revision B.01*, Gaussian, Inc., Wallingford CT, **2010**.

16. I. Fleming, *Frontier Orbitals and Organic Chemical Reactions*, Wiley, London, **1978**.
